# Understanding Small Molecule Activation Promoted by Heavier Benzene 1,4‐diides: Interplay Between Diradical Character and Aromaticity

**DOI:** 10.1002/chem.202501933

**Published:** 2025-06-23

**Authors:** Daniel González‐Pinardo, Rajendra S. Ghadwal, Israel Fernández

**Affiliations:** ^1^ Departmento de Química Orgánica and Centro de Innovación en Química Avanzada (ORFEO‐CINQA) Facultad de Ciencias Químicas Universidad Complutense de Madrid Madrid 28040 Spain; ^2^ Molecular Inorganic Chemistry and Catalysis Inorganic and Structural Chemistry Center for Molecular Materials Faculty of Chemistry Universität Bielefeld D‐33615 Bielefeld Germany

**Keywords:** aromaticity, bond activation, DFT/ab‐initio calculations, diradicals, main‐group

## Abstract

The intricate relationship between diradical character, aromaticity, and reactivity in annulated heavier Group 14 benzene‐1,4‐diides, that is, [(ADC)E]_2_ (E = Si, Ge, Sn), based on an anionic dicarbene framework, (ADC = PhC{N(Ar)C}_2_: Ar = aryl), has been investigated through Density Functional Theory and ab initio calculations. The diradical character of both homo‐ [(ADC)E]_2_ and heteroleptic [(ADC)_2_EE'] systems (E ≠ E') has been accurately computed, while the aromaticity of their corresponding closed‐shell (**CS**) and open‐shell (**OS**) singlet states has been evaluated using magnetic descriptors. Additionally, the key factors governing dihydrogen activation and cycloaddition with acetylene have been quantitatively analyzed in detail by applying the combination of the Activation Strain Model (ASM) of reactivity and Energy Decomposition Analysis (EDA) methods. The findings reveal a direct correlation between reactivity and diradical character, both of which increase down Group 14.

## Graphical abstract

The intricate relationship between diradical character, aromaticity, and reactivity in annulated heavier Group 14 benzene‐1,4‐diides (see picture) has been investigated through Density Functional Theory and ab initio calculations. A direct correlation between reactivity and diradical character, both of which increase down Group 14, is found.

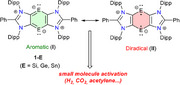

## 1 Introduction

The activation of strong bonds in small molecules (such as H₂, O₂, CO₂, and N₂) has traditionally relied on transition metal complexes, which excel at this task due to their ability to adopt multiple oxidation states and facilitate electron transfer.^[^ ^1^ ^]^ Over the past years, main‐group compounds have emerged as viable alternatives for the activation and functionalization of small molecules, primarily due to their relatively lower toxicity and cost.^[^ ^2^ ^]^ Singlet carbenes,^[^ ^3^ ^]^ and their Group 13 and 14 analogues^[^ ^4^ ^]^ and frustrated lewis pairs (FLPs)^[^ ^5^ ^]^ represent key examples that have demonstrated significant potential in this field, including catalytic transformations. As a result, these species have paved the way for metal‐free catalysis, enabling more environmentally sustainable chemical transformations.

**Scheme 1:**
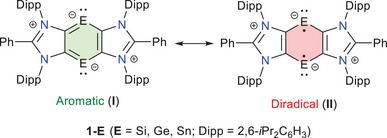
Main resonance structures contributing to the description of **1‐E** species.

In this context, Ghadwal and co‐workers recently reported a series of annulated heavier benzene‐1,4‐diides (**1‐E**, E = Si,^[^ ^6^ ^]^ Ge,^[^ ^7^ ^]^ Sn^[^ ^8^ ^]^) based on an anionic dicarbene (ADC = PhC{N(Dipp)C}_2_; Dipp = 2,6‐*i*Pr_2_C_6_H_3_) framework.^[^ ^9^ ^]^ These species feature a planar C_4_E_2_ ring with comparable C− E bond lengths, suggesting the presence of a 6π electron Hückel‐aromatic system (form **I** in Scheme 1). However, as is well‐known, the overlap between the p orbitals of carbon and heavier Group 14 elements becomes less efficient as one moves down the group.^[^ ^10^ ^]^ Thus, the diradical form **II** of **1‐E** becomes more significant in the order Si < Ge < Sn < Pb. Indeed, previous calculations suggest that the diradical character of these compounds steadily increases in the order Si < Ge < Sn. Nevertheless, the reported values for diradical character (as well as other calculated parameters) for **1‐E** should be treated with caution, as they were calculated using different computational methods.^[^ ^9^ ^]^ Therefore, a consistent evaluation of their diradical character remains lacking.

**Scheme 2:**
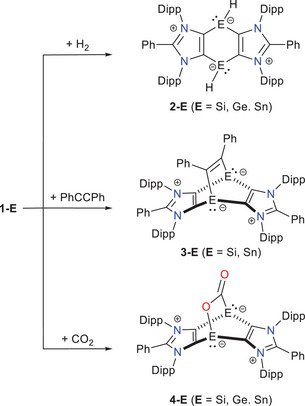
Representative small molecule activation reactions mediated by **1‐E** species.

Interestingly, these annulated heavy benzene‐1,4‐diides (**1**‐**E**) readily activate small molecules such as H_2_, PhC≡CPh,^[^ ^6^, ^7^, ^8^, ^9^ ^]^ or CO_2_^[^ ^11^ ^]^ at room temperature (Scheme 2). These mild reaction conditions arise mainly from their (formally) diradical open‐shell (**OS**) singlet nature. As the diradical character increases on moving down the Group 14, the reactivity trend Si < Ge < Sn is expected, which has been recently confirmed by us in the activation of CO_2_.^[^ ^11^ ^]^ In addition, the tin compound **1‐Sn** reacts immediately with diphenylacetylene at room temperature to quantitatively yield the 1,4‐distannabarrelene compound **3‐Sn** (Scheme 2). However, the analogous reaction of **1‐Si** with diphenylacetylene needs to be heated at 70 °C for five days in yielding **3‐Si**. Despite considerable research, the factors that control the bond activation by these heavy benzene‐1,4‐diides, as well as the influence of the diradical character and aromaticity on their reactivity, remain poorly understood. This gap in knowledge prompted us to undertake a comprehensive computational study with two main objectives: (i) to consistently evaluate the aromaticity and diradical character of the homoleptic species **1‐E**, as well as their (hitherto unknown) heteroleptic counterparts **1‐EE'** (**E** ≠ **E'**; **E** or **E'** = Si, Ge, Sn), and (ii) to quantitatively elucidate the factors that govern the reactivity of these species. To achieve this, we will employ the Activation Strain Model (ASM)^[^ ^12^ ^]^ of reactivity in combination with the Energy Decomposition Analysis (EDA)^[^ ^13^ ^]^ method. This combined approach has been pivotal in advancing our current understanding of fundamental processes in both organic^[^ ^14^ ^]^ and organometallic chemistry,^[^ ^15^ ^]^ particularly in reactions mediated by main‐group compounds.^[^ ^16^ ^]^

## 2 Results and Discussion

### 2.1 Diradical Character and Aromaticity

We initially focused on evaluating the diradical character of the title compounds. To accomplish this, we employed slightly modified model systems in which the bulky isopropyl substituents of the Dipp groups were replaced by methyl groups (*i.e*., 2,6‐dimethylphenyl groups = 2,6‐xylyl).

Previous studies on these systems have shown that the B3LYP‐D3 functional yields results consistent with experimental findings, particularly for singlet‐triplet energy gaps and reactivity trends.^[^ ^6^, ^7^, ^8^, ^9^, ^11^ ^]^ However, to ensure that our results and conclusions are not biased by the choice of functional, we performed an initial benchmark study on **1‐E** species using three widely used functionals: B3LYP‐D3, M06‐2X, and ωB97X‐D. These functionals differ in, among others, the rate of Hartree‐Fock exchange, which is crucial to the present study. We then performed calculations on **1‐Si**, **1‐Ge**, and **1‐Sn** in both their closed‐shell (**CS**) (aromatic) and **OS** (diradical) singlet states. The optimized geometries for these species do not differ significantly and align closely with the experimental (X‐ray) geometries^[^ ^9^ ^]^ (see Table S1 in the Supporting Information). The diradical character of the annulated heavier Group 14 benzene‐1,4‐diides progressively increases as one goes down in the Group 14. In all cases, the calculations involving **1‐Si** converged to the **CS** singlet state, likely due to its modest diradical character. In all cases, the energy difference between **CS** and **OS** singlet solutions for **1‐Si** is not applicable (N/A). In contrast, **1‐Sn** is consistently found to be a diradical in its ground state across all three functionals. Meanwhile, **1‐Ge** represents an intermediate case (Table 1). The data in Table 1 further indicate that the M06‐2X functional results in significantly larger energy differences between the **CS** and **OS** species compared to the B3LYP‐D3 or ωB97X‐D functionals, which provide similar values. This disparity is likely due to the high percentage of Fock exchange (54%) in the former (M06‐2X) functional, which tends to overestimate the stability of the diradical species, a known issue in multireference systems.^[^ ^17^ ^]^ As anticipated, the same issue is also evident in the computed Natural Orbital Occupations (n_LUNO_), which provide a direct estimate of the diradical character.^[^ ^18^ ^]^

**Table 1:** Computed free energy difference (ΔG, in kcal/mol) between the **CS** and **OS** singlet states of **1‐E** and their corresponding n_LUNO_ (in electrons) occupations.

		1‐Si	1‐Ge	1‐Sn
**ΔG(OS‐CS)**	M06‐2X	N/A^[a]^	−4.6	−15.3
	(ωB97X‐D)	N/A	(‐0.4)	(‐5.4)
	[B3LYP‐D3]	N/A	[0.0]	[‐2.2]
**n_LUNO_**	M06‐2X	N/A	0.40	0.71
	(ωB97X‐D)	N/A	(0.09)	(0.53)
	[B3LYP‐D3]	N/A	[0.00]	[0.43]

^[a]^ N.A. = not applicable

Although the results above are in qualitative agreement with the expectations,^[^ ^19^ ^]^ regardless of the functional used, we carried out (state‐specific) Complete Active Space Self‐Consistent Field (SS‐CASSCF) calculations using the (u)B3LYP‐D3 geometries to accurately assess the diradical character of the homoleptic and heteroleptic heavier benzene‐1,4‐diides. For this purpose, we selected a large active space of 10 electrons/10 orbitals (CASSCF[10,10]/def2‐TZVPP) to allow a significant degree of delocalization within the molecules. The computed natural orbital occupations of the respective highest occupied natural orbital (HONO) and lowest unoccupied natural orbital (LUNO) (Table 2) confirm the diradical character in all compounds, with the diradical character steadily increasing from **1‐Si** to **1‐Sn** (n_L__UNO_ values of 0.16 and 0.55 electrons, respectively). Moreover, our SS‐CASSCF calculations for the heteroleptic derivatives reveal that these compounds exhibit a diradical character that is intermediate between their corresponding homoleptic counterparts. For instance, the LUNO occupation for **1‐SiGe** is 0.19e, which lies between that of **1‐Si** (0.16e) and **1‐Ge** (0.23e). As anticipated, the diradical character increases in **1‐SiSn** (0.28e) and **1‐GeSn** (0.35e) due to the presence of the tin atom, ultimately reaching the highest diradical character in **1‐Sn** (0.55e). Based on the LUNO occupations, it is evident that the M06‐2X functional significantly overestimates the diradical character of these molecules, making it unsuitable for this type of calculation. In contrast, both ωB97X‐D and B3LYP‐D3 functionals provide reasonably accurate estimates of the diradical character of **1‐Sn**. However, they both struggle to accurately predict the occupations of **1‐Si** and **1‐Ge**, with ωB97X‐D showing slightly better reliability.

**Table 2:** HONO and LUNO occupations (in electrons) computed for **1‐E**/**1‐EE’** at theCASSCF[10,10]/def2‐TZVPP//PCM‐(u)B3LYP‐D3/def2‐SVP level.

	1‐Si	1‐SiGe	1‐Ge	1‐SiSn	1‐GeSn	1‐Sn
**n_HONO_**	1.85	1.81	1.78	1.73	1.65	1.45
**n_LUNO_**	0.16	0.19	0.23	0.28	0.35	0.55

Upon closer inspection of the computed configuration coefficients, two primary configurations emerge for the ground state of all species: the dominant doubly occupied [2222200000] configuration, alongside a smaller contribution from the double excitation [2222020000] (see Supporting Information). This double excitation is commonly encountered in **OS** singlet diradicals,^[^ ^19^ ^]^ further reinforcing the **OS** nature of these species. Notably, the contribution of the double excited configuration increases from **1‐Si** to **1‐Sn**, mirroring the trend previously discussed for the LUNO occupations.

To gain a deeper understanding of the aromaticity in both the **CS** (aromatic) and **OS** (diradical) singlet species, we first computed the Nuclear Independent Chemical Shift (NICS)^[^ ^20^ ^]^ values at the (3,+1) critical point of the C_4_E_2_ (or C_4_EE’) rings,^[^ ^21^ ^]^ using the corresponding out‐of‐plane component computed 1 Å above this point (NICS(1)_zz_), which is considered a reliable measure of magnetic aromaticity.^[^ ^22^ ^]^ Our results indicate that all **CS** singlet species exhibit aromaticity (Table 3). However, we observed that the strength of aromaticity steadily decreases as we move down the Group 14, ranging from a value of ‐20.4 ppm in **1‐Si** to a significantly lower value of ‐9.8 ppm in **1‐Sn**. This trend is consistent with the progressively weaker overlap between the p orbitals of C and E atoms as one moves down Group 14, leading to more restricted delocalization of the π‐electrons within the six‐membered ring. In the case of the located diradical species, specifically those containing tin atoms, we found that in the heteroleptic **1‐SiSn** and **1‐GeSn** systems, π‐aromaticity is still preserved despite their diradical character. This contrasts with the situation observed in **1‐Sn**, which can be considered as a nonaromatic compound (NICS(1)_zz_ = 1.1 ppm).

**Table 3:** Computed NICS(1)_zz_ values (in ppm) for **1‐E**/**1‐EE’** in their **CS** and **OS** singlet states computed at the GIAO‐(u)B3LYP‐D3/def2‐SVP//PCM(benzene)‐(u)B3LYP‐D3/def2‐SVP level.

	1‐Si	1‐SiGe	1‐Ge	1‐SiSn	1‐GeSn	1‐Sn
**CS**	−20.4	−18.9	−17.2	−15.1	−13.4	−9.8
**OS**	−	−	−	−14.0	−8.0	1.1

**Figure 1:**
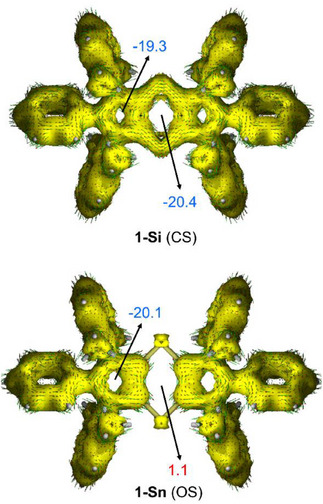
Computed ACID plots (isosurface value of 0.03 a.u.) and NICS(1)_zz_ values (in ppm) for **CS** singlet **1‐Si** (top) and **OS** singlet (diradical) **1‐Sn** (bottom).

Further evidence supporting the NICS‐based aromatic nature of title compounds is provided by the Anisotropy of the Induced Current Density (AICD)^[^ ^23^ ^]^ method. By applying this approach to the extreme cases represented by **1‐Si** and **1‐Sn** (Figure 1), it becomes clear that **1‐Si** exhibits a clear diatropic (*i.e*., aromatic) current, resulting from the delocalization of 14‐π electrons over the cyclic system. This system includes not only the C_4_Si_2_ moiety but also the adjacent heterocyclic rings. In stark contrast, the ring current is disrupted at the tin atoms in diradical **1‐Sn**, causing electron delocalization to be confined solely to the isolated 6π‐aromatic heterocycles. This distinction is reflected in the computed NICS values mentioned earlier, as well as in the corresponding values for the heterocycles, which are highly negative in both cases (see Figure 1).

### 2.2 Reactivity

After assessing the diradical character and aromaticity, we turned our attention to the reactivity of these annulated heavy benzene‐1,4‐diides. In particular, we examined their reactions with dihydrogen and acetylene (used as a model of diphenylacetylene) as representative small molecule activations.

**Scheme 3:**
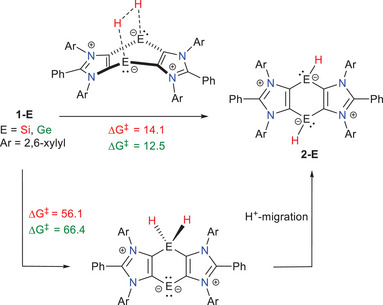
Alternative concerted/stepwise pathways for the H_2_‐activation reaction mediated by **1‐** **Si** (green) and **1‐Ge** (red). Relative activation free energies (ΔG^‡^, at 298 K) were computed at the CPCM(benzene)‐DLPNO‐CCSD(T)/def2‐TZVPP//PCM(benzene)‐(u)B3LYP‐D3/def2‐SVP level.

The activation of dihydrogen was initially investigated. As previously mentioned, **1‐E** species readily react with H_2_ at room temperature, yielding stable cyclic bis‐hydridometallynes **2‐E** (Scheme 2).^[^ ^6^, ^7^, ^8^, ^9^ ^]^ Two possible pathways for dihydrogen splitting can be envisaged: (i) a concerted process involving the cooperative action of both E atoms or (ii) a stepwise mechanism, where dihydrogen splitting occurs at one E center (via an oxidative addition‐like process), followed by an H‐migration step (Scheme 3). Our calculations for processes involving **1‐Si** and **1‐Ge**, using the highly accurate conductor‐like polarizable continuum (CPCM) (benzene)‐DLPNO‐CCSD(T)/def2‐TZVPP//PCM(benzene)‐(u)B3LYP‐D3/def2‐SVP method, clearly show that the stepwise pathway is kinetically unrealistic (ΔG^‡^ > 55 kcal/mol) and can thus be ruled out. In contrast, the concerted pathway requires a relatively low activation barrier (up to ca. 14 kcal/mol for **1‐Si**), which aligns with the observed reaction occurring at room temperature.

**Figure 2:**
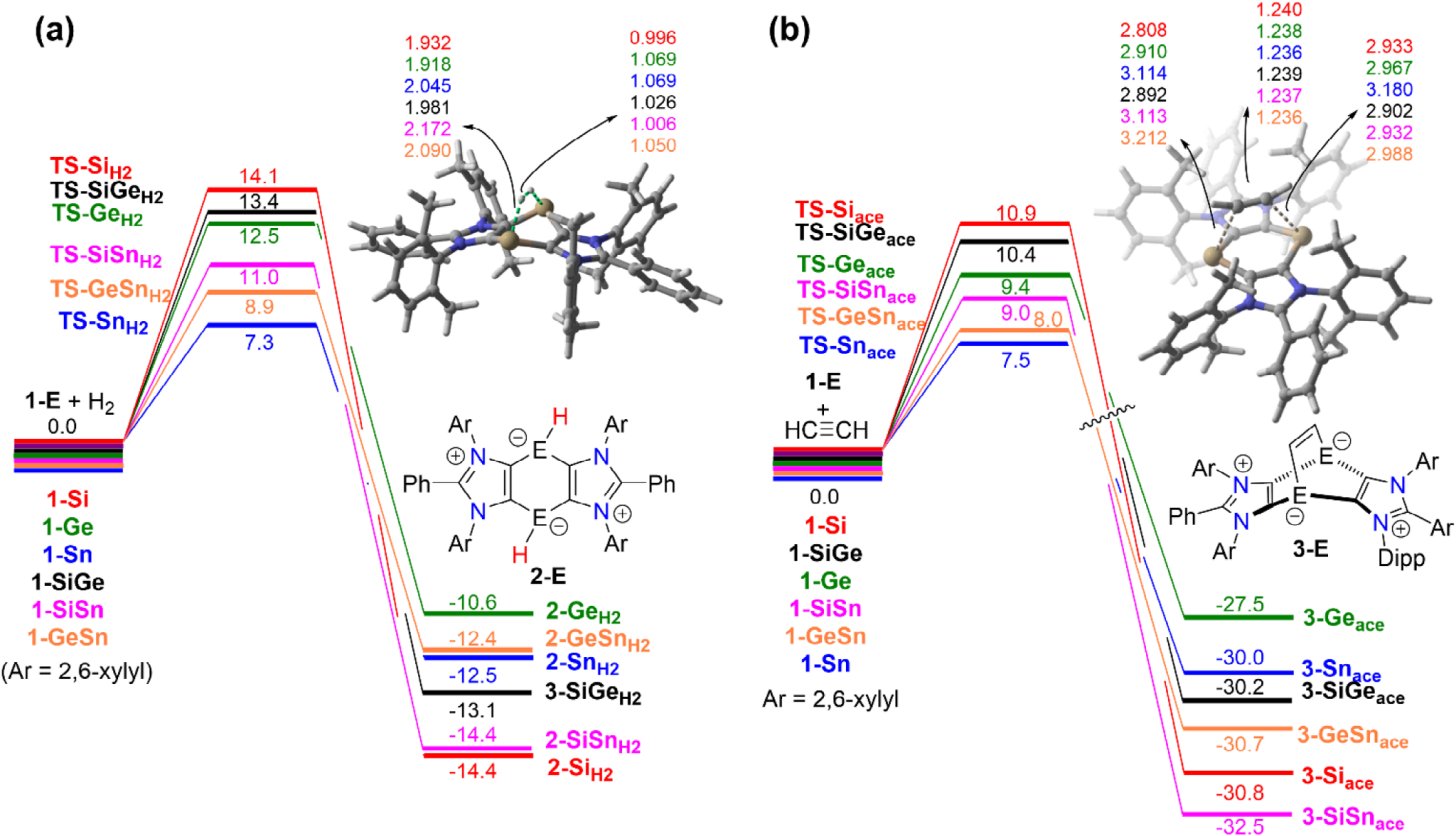
Computed reaction profiles for the H_2_‐activation reaction (a) and [4 + 2]‐cycloaddition involving acetylene mediated by **1‐E** and **1‐EE’**. Relative free energies (ΔG, at 298 K) and bond distances are given in kcal/mol and angstroms, respectively. All data were computed at the CPCM(benzene)‐DLPNO‐CCSD(T)/def2‐TZVPP//PCM(benzene)‐(u)B3LYP‐D3/def2‐SVP level.

Figure 2a presents the computed reaction profiles for the dihydrogen activation reactions for all **1‐E**/**1‐EE’** species. In each case, the dihydrogen splitting occurs in a concerted manner via the transition state **TS‐E_H2_**, which is associated with the cleavage of the H─H bond and the simultaneous formation of two new E─H bonds. This process leads to the exergonic formation of the corresponding cyclic bis‐hydridometallynes **2‐E**/**2‐EE’**, with Gibbs free energy changes (ΔG_R_) ranging from ‐10.6 to ‐14.4 kcal/mol. Interestingly, a clear reactivity trend emerges from the computed activation barriers shown in Figure 2. Specifically, the activation barrier for the concerted dihydrogen activation systematically decreases as one moves down the Group 14. Thus, while the lightest system, **1‐Si,** exhibits the highest barrier (ΔG^‡^ = 14.1 kcal/mol) in the series, the analogous process involving the **OS** singlet **1‐Sn** proceeds with the lowest barrier (ΔG^‡^ = 7.3 kcal/mol). As expected, the **1‐Ge** system falls between these extremes, with an intermediate barrier of ΔG^‡^ = 12.5 kcal/mol. Similarly, the heteroleptic systems present barrier heights that are also intermediate between their respective homoleptic counterparts. These findings suggest a direct correlation between increased diradical character and enhanced reactivity toward dihydrogen activation. We will revisit this important conclusion later.

**Figure 3:**
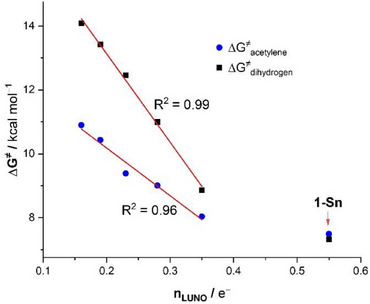
Correlation between the computed activation barriers for H_2_ activation (blue circles) and acetylene cycloaddition (black squares) and the CASSCF‐computed LUNO occupations.

We then investigated the [4 + 2]‐cycloaddition reaction between **1‐E**/**1** **‐EE’** and acetylene, using it as a model of diphenylacetylene employed in the experiments. As mentioned earlier, this process leads to the formation of the barrelene‐type compounds **3‐E** (Scheme 2).^[^ ^9^ ^]^ Our calculations indicate that this reaction also proceeds in a concerted manner through a highly synchronous six‐membered transition state (**TS‐E_ace_**), ultimately yielding the corresponding cycloadduct in a highly exergonic manner (Figure 2b). Compared to the H_2_‐activation, this transformation is significantly more exergonic, which is a predictable outcome considering the greater E−C bond strength of the newly formed bonds relative to E−H bonds. For instance, the bond dissociation energy of the Si−C bond (435 kJ/mol) is substantially higher than that of the Si−H bond (298 kJ/mol).^[^ ^24^ ^]^ A similar reactivity trend to that observed for H_2_ activation is also evident in this process. Specifically, the activation barrier steadily decreases as going down in the Group 14, with **1‐Si** exhibiting the highest barrier, while the diradical **1‐Sn** becomes the most reactive species. Thus, the computed barrier heights for these two distinct reactions, H_2_ splitting and [4 + 2]‐cycloaddition, clearly demonstrate that the reactivity of these annulated heavy benzene‐1,4‐diides increases with their relative diradical character. This is further supported by the strong linear correlations observed when plotting the different activation barriers (ΔG^‡^) against the CASSCF‐computed LUNO occupations (R^2^ = 0.96 and 0.99 for H_2_ activation and acetylene cycloaddition, respectively, see Figure 3). Notably, in this plot, the **1‐Sn** deviates from the correlation, likely due to the B3LYP‐D3 functional's limited accuracy in describing the diradical character of this species.

**Figure 4:**
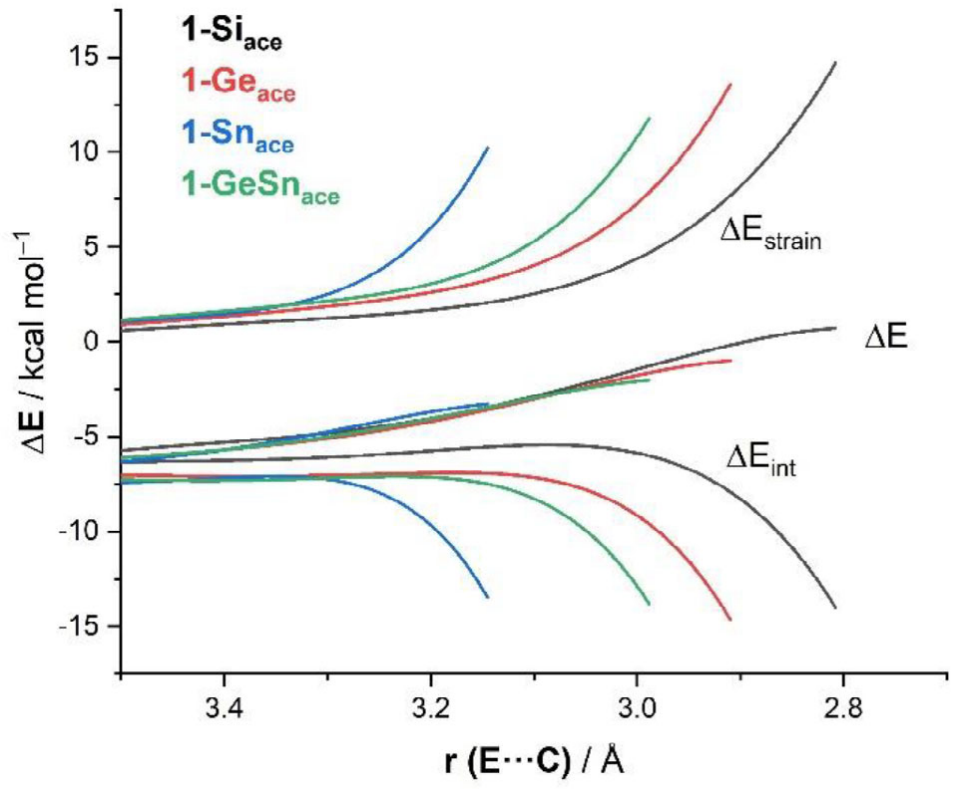
Comparative ASDs for the reactions of acetylene with **1‐E** (E = Si, Ge, Sn) and **1‐GeSn** projected onto the shortest C···E bond‐forming distance. All data have been computed at the ZORA‐B3LYP‐D3/TZ2P//PCM(benzene)‐B3LYP‐D3/def2‐SVP level.

The Activation Strain Model of reactivity^[^ ^12^ ^]^ was applied to gain a detailed and quantitative understanding of the observed reactivity trend. This approach decomposes the electronic energy (ΔE) into two key contributions: the strain energy (ΔE_strain_), which arises from the structural deformation of the individual reactants, and the interaction energy (ΔE_int_), which describes the stabilizing interactions between increasingly deformed reactants along the reaction coordinate. Figure 4 shows the Activation Strain Diagrams (ASDs) for the acetylene cycloaddition reaction involving the homoleptic species **1‐E**, along with compound **1‐GeSn** as a representative heteroleptic case. These diagrams trace the reaction progress from the initial stages of the reaction up to the respective transition states, projected onto the E···C bond‐forming distance (E = Si, Ge, Sn for **1‐E** and Sn for **1‐GeSn**). From the data given in Figure 4, it becomes clear that the strain energy does not dictate the reactivity trend. In fact, the least reactive system, **1‐Si,** exhibits the lowest (*i.e*., less destabilizing) ΔE_strain_, while **1‐Sn**, the most reactive system, displays the highest deformation energy. This behavior closely mirrors our previous findings for the analogous reaction of **1‐E** with CO_2_.^[^ ^11^ ^]^ In contrast, the interaction energy (ΔE_int_) emerges as the primary factor governing the observed reactivity trend, showing a steady increase in stabilizing interactions in the order of **1‐Si** < **1‐Ge** < **1‐GeSn** < **1‐Sn**. For instance, at a consistent E···C bond‐forming distance of 3.14 Å,^[^ ^25^ ^]^ ΔE_int_ follows the trend: **1‐Si** (‐5.5 kcal/mol) < **1‐Ge** (‐6.9 kcal/mol) < **1‐GeSn** (‐7.6 kcal/mol) < **1‐Sn** (‐13.5 kcal/mol). A similar trend was also observed in the ASDs for the analogous H_2_ activation reaction (see Supporting Information), further reinforcing the mechanistic similarity between these transformations.

**Figure 5:**
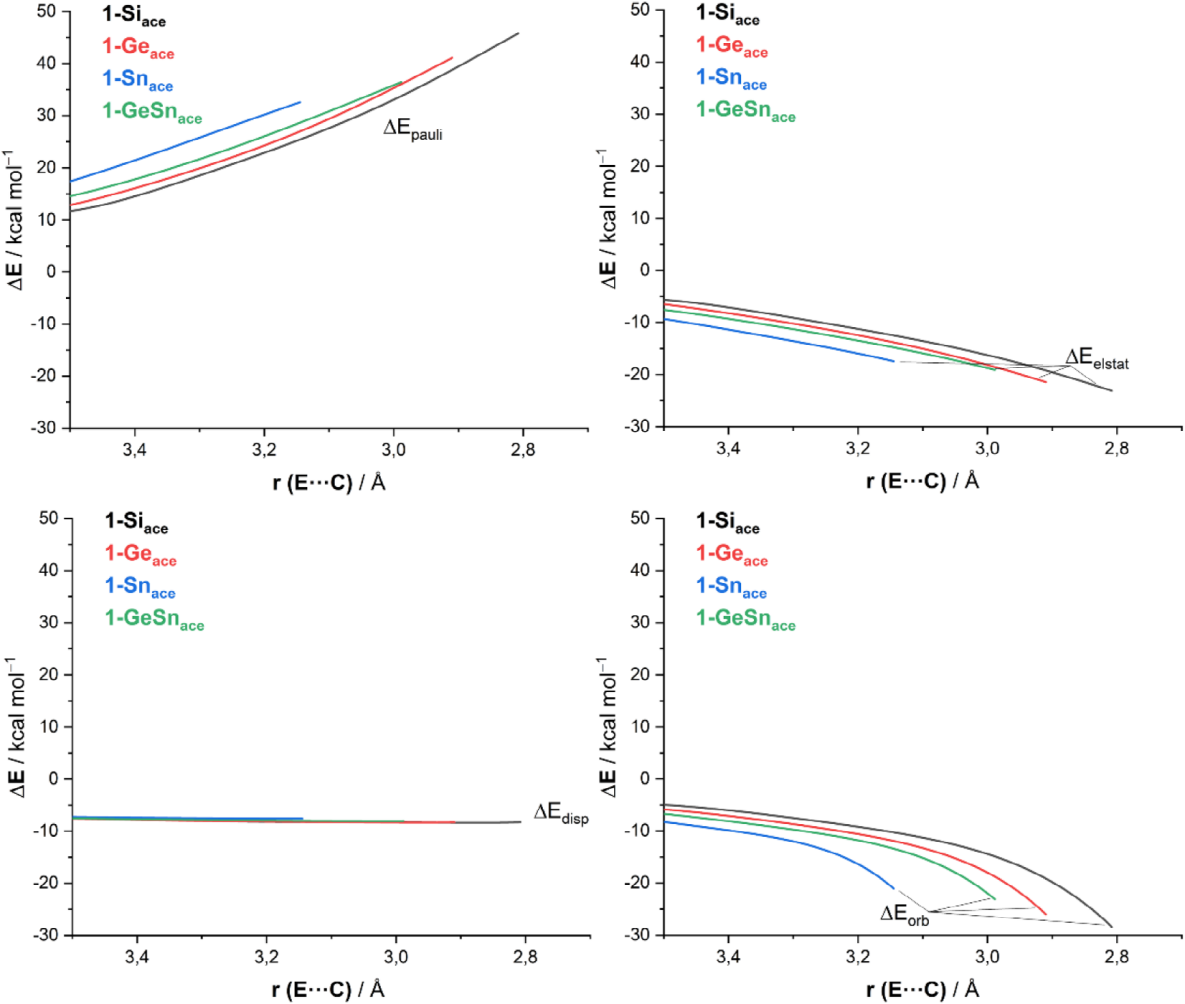
Evolution of the EDA terms along the reaction coordinate for the **r**eactions of acetylene with **1‐E** (E = Si, Ge, Sn) and **1‐GeSn** and projected onto the shortest C···E bond‐forming distance. All data have been computed at the ZORA‐B3LYP‐D3/TZ2P//PCM(benzene)‐B3LYP‐D3/def2‐SVP level.

The interaction energy plays a crucial role in this process and warrants further analysis. To better understand the factors contributing to the stronger ΔE_int_ observed in reactions involving the heaviest **1**‐**E** systems, we applied the EDA method.^[^ ^13^ ^]^ This approach breaks down the interaction energy (ΔE_int_) between reactants into distinct components: the classical electrostatic interaction (ΔE_elstat_), the Pauli repulsion (ΔE_Pauli_) arising from the repulsion between occupied orbitals of both deformed reactants, the orbital interaction (ΔE_orb_) that accounts for charge transfer and polarization, and the dispersion interactions (ΔE_disp_). Figure 5 illustrates the evolution of these EDA terms for reactions involving **1‐E** and **1‐GeSn**, tracking their progression from the initial stages to the corresponding transition states, mapped onto the E···C bond‐forming distance. The data in Figure 5 reveal that the observed trend in ΔE_int_ is not driven by the Pauli repulsion term, which, as expected, follows the opposite trend due to the increasing atom size when going down in Group. Likewise, dispersion interactions do not significantly contribute, as the ΔE_disp_ term remains relatively small and nearly identical across all processes. Instead, the trend in ΔE_int_ primarily originates from orbital interactions between the deformed reactants throughout the reaction coordinate. These interactions become progressively stronger in going down in the Group 14, driving the overall increase in interaction energy. A similar trend is observed for the electrostatic attraction term ΔE_elstat_, although its contribution is considerably smaller compared to the orbital interactions.

**Figure 6:**
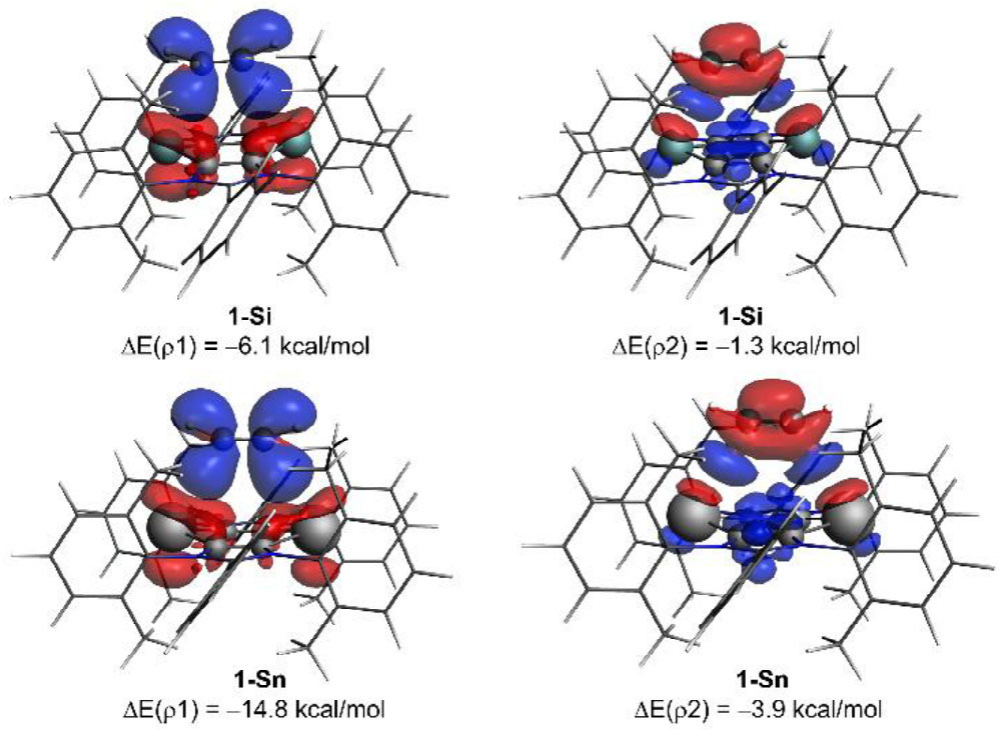
Contour plots of the NOCV deformation densities ρ (isosurface value of 0.001 a.u.) and the associated energies ΔE(ρ) for the main orbital interactions between acetylene and **1‐Si** (top) and **1‐Sn** (bottom) at the same consistent E···C bond‐forming distance of 3.14 Å. The electronic charge flows from red to blue. All data were computed at the ZORA‐B3LYP‐D3/TZ2P//PCM(benzene)‐B3LYP‐D3/def2‐SVP level.

The nature of the main orbital interactions responsible for the ΔE_orb_ can be further analyzed using the Natural Orbital for Chemical Valence (NOCV)^[^ ^26^ ^]^ extension of the EDA method. This approach allows for both the visualization and quantification of these orbital interactions. According to the NOCV(EDA) method, two primary orbital interactions dominate the ΔE_orb_ term: the π‐HOMO(**1‐E**)→π*‐LUMO(C≡C) donation (denoted as ρ1 in Figure 6) and the backdonation involving the reverse π‐HOMO(C≡C)→π*‐LUMO(**1‐E**) molecular orbital interaction (denoted as ρ2). The data in Figure 6, which present the corresponding stabilizing energies (ΔE(ρ)) for the extreme cases of **1‐Si** and **1‐Sn** computed at a consistent C···E bond‐forming distance of 3.14 Å, show that the strength of ρ1 is greater than ρ2 in both cases. This suggests that the **1‐E** species are better electron donors than acceptors. Interestingly, the key π‐HOMO(**1‐E**)→π*‐LUMO(C≡C) molecular orbital interaction ρ1 becomes progressively stronger as one moves down the Group 14: ΔE(ρ1) = ‐6.1 kcal/mol (**1‐Si**) < ‐7.3 kcal/mol (**1‐Ge**) < ‐8.6 kcal/mol (**1‐GeSn**) < ‐14.8 kcal/mol (**1‐Sn**). This trend parallels the total ΔE_orb_ term, as well as ΔE_int_, and ultimately the activation barriers. Based on our ASM‐EDA(NOCV) calculations, we conclude that the increased reactivity of the heavier **1‐E** systems down Group 14 is primarily due to an enhanced π‐donor ability. This increases the strength of the key π‐HOMO(**1‐E**)→π*‐LUMO(C≡C) molecular orbital interaction, which is a consequence of their higher diradical character.

## 3 Conclusion

From the computational study presented herein, the following conclusions can be drawn:

- The diradical character of the annulated heavier Group 14 benzene‐1,4‐diides progressively increases as one goes down in the Group 14. Specifically, the homoleptic **1‐Si** shows a negligible diradical character (n_LUNO_ = 0.16e), while **1‐Sn** exhibits a significant **OS** singlet nature (n_LUNO_ = 0.55e).
- In contrast, the aromaticity of the system follows the opposite trend, as confirmed by NICS(1)_zz_ and induced ring‐current calculations.
- Both properties are driven by decreasing overlap between the p_π_ orbitals of carbon and the heavier Group 14 elements as one moves down the Group.
- As a result, the heavier systems demonstrate enhanced reactivity compared to their lighter counterparts in both H_2_‐splitting and acetylene cycloaddition reactions.
- According to the ASM‐EDA(NOCV) method, this increased reactivity is primarily attributed to a stronger interaction between the deformed reactants in the heaviest systems. This is mainly driven by more stabilizing π‐HOMO(**1‐E**)→π*‐LUMO(C≡C) molecular orbital interactions, together with stronger electrostatic attractions (albeit to a lesser extent).

Through accurate, state‐of‐the‐art calculations, our work provides a consistent evaluation of the diradical character of these main‐group compounds and its impact on their poorly understood reactivity in small molecule activation.

### 3.1 Computational Details

Geometry optimizations of the molecules were performed without symmetry constraints using the Gaussian16 (RevB.01) suite of program^[^ ^27^ ^]^ at the dispersion‐corrected (u)B3LYP^[^ ^28^ ^]^‐D3^[^ ^29^ ^]^/def2‐SVP^[^ ^30^ ^]^ level considering solvent effects with the Polarization Continuum Model (PCM)^[^ ^31^ ^]^ method. Reactants and products were characterized by frequency calculations and have positive definite Hessian matrices. Transition states show only one negative eigenvalue in their diagonalized force constant matrices, and their associated eigenvectors were confirmed to correspond to the motion along the reaction coordinate under consideration using the Intrinsic Reaction Coordinate (IRC) method.^[^ ^32^ ^]^ Energy refinements were carried out by means of single‐point calculations at the Domain Based Local Pair‐Natural Coupled‐Cluster (DLPNO‐CCSD(T), using NormalPNO)^[^ ^33^ ^]^ with the ORCA 5.0.3^[^ ^34^ ^]^ program using the def2‐TZVPP^[^ ^29^ ^]^ basis set on the PCM‐B3LYP‐D3/def2‐SVP geometries. Solvent effects were also considered during the single‐point calculations by means of the CPCM for benzene as implemented in ORCA. This level is denoted CPCM(benzene)‐DLPNO‐CCSD(T)/def2‐TZVPP//PCM(benzene)‐B3LYP‐D3/def2‐SVP. The computed thermochemistry data were corrected following Grimme's quasi‐harmonic (QHA) model for entropy^[^ ^35^ ^]^ with a frequency cutoff value of 100.0 cm^−1^ using the GoodVibes^[^ ^36^ ^]^ program at 298.15 K and a standard concentration of 1 M.

SS‐CASSCF calculations were performed in ORCA 5.0.3. Guess orbitals were generated at the (u)B3LYP‐D3‐def2‐SVP, and a singlet ground state wavefunction with a [10,10] active space was obtained at the CASSCF/def2‐TZVPP level.

#### 3.1.1 ASM of Reactivity and EDA

Within the ASM method,^[^ ^12^ ^]^ also known as the distortion/interaction model,^[^ ^12b^ ^]^ the potential energy surface ∆E(ζ) is decomposed along the reaction coordinate, ζ, into two contributions, namely the strain ∆E_strain_(ζ) associated with the deformation (or distortion) required by the individual reactants during the process and the interaction ∆E_int_(ζ) between these increasingly deformed reactants:

$$\Delta E\left(\zeta \right)=\Delta E_{\mathrm{strain}}\left(\zeta \right)+\Delta E_{\mathrm{int}}\left(\zeta \right)$$

Within the EDA method,^[^ ^13^ ^]^ the interaction energy can be further decomposed into the following chemically meaningful terms:

$$\Delta E_{\mathrm{int}}\left(\zeta \right)=\Delta V_{\mathrm{elstat}}\left(\zeta \right)+\Delta E_{\mathrm{Pauli}}\left(\zeta \right)+\Delta E_{\mathrm{orb}}\left(\zeta \right)+\Delta E_{\text{disp}}\left(\zeta \right)$$

The term ∆V_elstat_ corresponds to the classical electrostatic interaction between the unperturbed charge distributions of the deformed reactants and is usually attractive. The Pauli repulsion ∆E_Pauli_ comprises the destabilizing interactions between occupied orbitals and is responsible for any steric repulsion. The orbital interaction ∆E_orb_ accounts for bond pair formation, charge transfer (interaction between occupied orbitals on one moiety with unoccupied orbitals on the other, including HOMO‐LUMO interactions), and polarization (empty‐occupied orbital mixing on one fragment due to the presence of another fragment). Finally, the ∆E_disp_ term accounts for the interactions coming from dispersion forces. Moreover, the NOCV ^[^ ^26^ ^]^ extension of the EDA method has also been used to further partition the ∆E_orb_ term. The EDA‐NOCV approach provides pairwise energy contributions for each pair of interacting orbitals to the total bond energy.

The program package ADF^[^ ^37^ ^]^ was used for EDA calculations using the optimized PCM‐(u)B3LYP‐D3/def2‐SVP geometries at the same DFT level in conjunction with a triple‐ζ‐quality basis set using uncontracted Slater‐type orbitals (STOs) augmented by two sets of polarization functions with a frozen‐core approximation for the core electrons.^[^ ^38^ ^]^ Auxiliary sets of s, p, d, f, and g STOs were used to fit the molecular densities and to represent the Coulomb and exchange potentials accurately in each SCF cycle.^[^ ^39^ ^]^ Scalar relativistic effects were incorporated by applying the zeroth‐order regular approximation (ZORA).^[^ ^40^ ^]^ This level of theory is denoted ZORA‐B3LYP‐D3/TZ2P//PCM(benzene)‐B3LYP‐D3/def2‐SVP.

## Conflict of Interest

The authors declare no conflict of interest.

## Supporting information



Supporting Information

## Data Availability

The data that support the findings of this study are available from the corresponding author upon reasonable request.
