# Supplementary material for: Understanding Small Molecule Activation Promoted by Heavier Benzene 1,4‐diides: Interplay Between Diradical Character and Aromaticity
Source: Chemistry. 2025 Jun 23;31(45):e202501933. doi: 10.1002/chem.202501933 (PMC12351437; doi:10.1002/chem.202501933)
Supplement: Supplementary file 1 — Supporting Information [file CHEM-31-e202501933-s001.pdf]

Understanding Small Molecule Activation Promoted by Heavier Benzene 1,4-diides: Interplay between Diradical Character and Aromaticity

Daniel González-Pinardo,[a] Rajendra S. Ghadwal, [b] and Israel Fernández*[a]

[a] Departamento de Química Orgánica I and Centro de Innovación en Química Avanzada (ORFEO-CINQA), Facultad de Ciencias Químicas, Universidad Complutense de Madrid, 28040-Madrid, Spain

e-mail: israel@quim.ucm.es

[b] Molecular Inorganic Chemistry and Catalysis, Inorganic and Structural Chemistry, Center for Molecular Materials, Faculty of Chemistry, Universität Bielefeld, D-33615-Bielefeld (Germany).

e-mail: rghadwal@uni-bielefeld.de

Contents:

1. X-Ray—DFT benchmark……………………………………………………………….S2
2. CASSCF electron density matrix and configuration coefficients……………………….S3
3. ASM and EDA—Dihydrogen activation…………………………………………………S9
4. Cartesian coordinates and energies…………………………….……………………...S10

1. X-Ray—DFT benchmark

Table S1. Comparison between X-Ray geometries and computed distances and angles with different functionals of **1-E** (**E** = Si, Ge, Sn). All values are represented in angstroms and degrees, respectively. Open-shell geometry values are represented between parentheses.

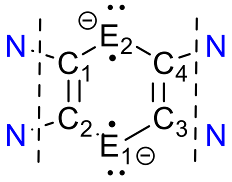

1-E (**E** = Si, Ge, Sn)

Coordinates	X-Ray	B3LYP-D3	M062X	wB97X-D
**1-Si**				
Si1-C2	1.851	1.869	1.862	1.852
Si1-C3	1.861	1.869	1.862	1.852
C1-C2	1.408	1.426	1.422	1.421
C2-Si1-C3	92.8	93.4	92.6	93.5
**1-Ge**				
Ge1-C1	1.963	1.965	1.953 (1.988)	1.938 (1.945)
Ge1-C2’	1.960	1.965	1.953 (1.988)	1.938 (1.945)
C1-C2	1.394	1.420	1.416 (1.404)	1.416 (1.413)
C2-Ge1-C3	91.4	92.1	92.0 (91.7)	92.2 (92.2)
**1-Sn**				
Sn1-C1	2.202	2.212 (2.241)	2.194 (2.253)	2.169 (2.219)
Sn1-C2	2.205	2.212 (2.241)	2.194 (2.253)	2.169 (2.219)
C1-C2	1.380	1.413 (1.404)	1.409 (1.393)	1.409 (1.396)
C2-Sn1-C3	90.8	90.6 (90.8)	90.5 (89.9)	90.8 (90.6)

2. CASSCF electron density matrix and configuration coefficients

Table S2. CAS-SCF electron density matrix and configuration coefficients of **1-E/1-EE'**, corresponding to their ground state (S_0). All data was computed at the CAS-SCF/def2TZVPP // PCM(benzene)-(u)B3LYP-D3/def2-SVP level of theory.

**Si**	1	2	3	4	5	6	7	8	9	10
1	1.98	0.00	0.00	0.00	0.00	0.00	0.00	0.00	0.00	0.00
2	0.00	1.98	0.00	0.00	0.00	0.00	0.00	0.00	0.00	0.00
3	0.00	0.00	1.95	0.00	0.00	0.00	0.00	0.00	0.00	0.00
4	0.00	0.00	0.00	1.93	0.00	0.00	0.00	0.00	0.00	0.00
5	0.00	0.00	0.00	0.00	1.85	0.00	0.00	0.00	0.00	0.00
6	0.00	0.00	0.00	0.00	0.00	0.16	0.00	0.00	0.00	0.00
7	0.00	0.00	0.00	0.00	0.00	0.00	0.08	0.00	0.00	0.00
8	0.00	0.00	0.00	0.00	0.00	0.00	0.00	0.05	0.00	0.00
9	0.00	0.00	0.00	0.00	0.00	0.00	0.00	0.00	0.01	0.00
10	0.00	0.00	0.00	0.00	0.00	0.00	0.00	0.00	0.00	0.01

Si

ROOT 0: E= -2716.5596545777 Eh

0.85669 [0]: 2222200000

0.04922 [6]: 2222020000

0.01818 [27]: 2221111000

0.01402 [217]: 2211201100

0.01012 [76]: 2220202000

0.00965 [168]: 2212110100

0.00435 [3925]: 1122210100

0.00363 [432]: 2202202000

0.00254 [1800]: 2022220000

**Ge**	1	2	3	4	5	6	7	8	9	10
1	1.99	0.00	0.00	0.00	0.00	0.00	0.00	0.00	0.00	0.00
2	0.00	1.99	0.00	0.00	0.00	0.00	0.00	0.00	0.00	0.00
3	0.00	0.00	1.95	0.00	0.00	0.00	0.00	0.00	0.00	0.00
4	0.00	0.00	0.00	1.92	0.00	0.00	0.00	0.00	0.00	0.00
5	0.00	0.00	0.00	0.00	1.78	0.00	0.00	0.00	0.00	0.00
6	0.00	0.00	0.00	0.00	0.00	0.23	0.00	0.00	0.00	0.00
7	0.00	0.00	0.00	0.00	0.00	0.00	0.07	0.00	0.00	0.00
8	0.00	0.00	0.00	0.00	0.00	0.00	0.00	0.05	0.00	0.00
9	0.00	0.00	0.00	0.00	0.00	0.00	0.00	0.00	0.01	0.00
10	0.00	0.00	0.00	0.00	0.00	0.00	0.00	0.00	0.00	0.01

Ge

ROOT 0: E= -6289.4358666336 Eh

0.82568 [0]: 2222200000

0.08801 [6]: 2222020000

0.01553 [217]: 2211201100

0.01521 [27]: 2221111000

0.00978 [168]: 2212110100

0.00883 [76]: 2220202000

0.00385 [432]: 2202202000

0.00264 [80]: 2220200200

**Sn**	1	2	3	4	5	6	7	8	9	10
1	1.99	0.00	0.00	0.00	0.00	0.00	0.00	0.00	0.00	0.00
2	0.00	1.99	0.00	0.00	0.00	0.00	0.00	0.00	0.00	0.00
3	0.00	0.00	1.94	0.00	0.00	0.00	0.00	0.00	0.00	0.00
4	0.00	0.00	0.00	1.93	0.00	0.00	0.00	0.00	0.00	0.00
5	0.00	0.00	0.00	0.00	1.45	0.00	0.00	0.00	0.00	0.00
6	0.00	0.00	0.00	0.00	0.00	0.55	0.00	0.00	0.00	0.00
7	0.00	0.00	0.00	0.00	0.00	0.00	0.06	0.00	0.00	0.00
8	0.00	0.00	0.00	0.00	0.00	0.00	0.00	0.05	0.00	0.00
9	0.00	0.00	0.00	0.00	0.00	0.00	0.00	0.00	0.02	0.00
10	0.00	0.00	0.00	0.00	0.00	0.00	0.00	0.00	0.00	0.02

Sn

ROOT 0: E= -2565.4160174080 Eh

0.67169 [0]: 2222200000

0.25027 [6]: 2222020000

0.01488 [217]: 2211201100

0.00573 [257]: 2211021100

0.00543 [76]: 2220202000

0.00506 [27]: 2221111000

0.00502 [168]: 2212110100

0.00368 [432]: 2202202000

0.00354 [224]: 2211200011

0.00324 [80]: 2220200200

0.00291 [436]: 2202200200

SiGe	1	2	3	4	5	6	7	8	9	10
1	1.99	0.00	0.00	0.00	0.00	0.00	0.00	0.00	0.00	0.00
2	0.00	1.98	0.00	0.00	0.00	0.00	0.00	0.00	0.00	0.00
3	0.00	0.00	1.95	0.00	0.00	0.00	0.00	0.00	0.00	0.00
4	0.00	0.00	0.00	1.93	0.00	0.00	0.00	0.00	0.00	0.00
5	0.00	0.00	0.00	0.00	1.81	0.00	0.00	0.00	0.00	0.00
6	0.00	0.00	0.00	0.00	0.00	0.19	0.00	0.00	0.00	0.00
7	0.00	0.00	0.00	0.00	0.00	0.00	0.07	0.00	0.00	0.00
8	0.00	0.00	0.00	0.00	0.00	0.00	0.00	0.05	0.00	0.00
9	0.00	0.00	0.00	0.00	0.00	0.00	0.00	0.00	0.01	0.00
10	0.00	0.00	0.00	0.00	0.00	0.00	0.00	0.00	0.00	0.01

SiGe

ROOT 0: E= -4502.9958109455 Eh

0.84180 [0]: 2222200000

0.06991 [6]: 2222020000

0.01639 [27]: 2221111000

0.01491 [217]: 2211201100

0.00942 [168]: 2212110100

0.00930 [76]: 2220202000

0.00389 [432]: 2202202000

**SiSn**	1	2	3	4	5	6	7	8	9	10
1	1.99	0.00	0.00	0.00	0.00	0.00	0.00	0.00	0.00	0.00
2	0.00	1.99	0.00	0.00	0.00	0.00	0.00	0.00	0.00	0.00
3	0.00	0.00	1.95	0.00	0.00	0.00	0.00	0.00	0.00	0.00
4	0.00	0.00	0.00	1.93	0.00	0.00	0.00	0.00	0.00	0.00
5	0.00	0.00	0.00	0.00	1.73	0.00	0.00	0.00	0.00	0.00
6	0.00	0.00	0.00	0.00	0.00	0.28	0.00	0.00	0.00	0.00
7	0.00	0.00	0.00	0.00	0.00	0.00	0.07	0.00	0.00	0.00
8	0.00	0.00	0.00	0.00	0.00	0.00	0.00	0.05	0.00	0.00
9	0.00	0.00	0.00	0.00	0.00	0.00	0.00	0.00	0.01	0.00
10	0.00	0.00	0.00	0.00	0.00	0.00	0.00	0.00	0.00	0.01

GeSn

ROOT 0: E= -2640.9800257670 Eh

0.80143 [0]: 2222200000

0.11321 [6]: 2222020000

0.01558 [217]: 2211201100

0.01334 [27]: 2221111000

0.00902 [168]: 2212110100

0.00821 [76]: 2220202000

0.00412 [432]: 2202202000

0.00270 [80]: 2220200200

0.00253 [257]: 2211021100

**GeSn**	1	2	3	4	5	6	7	8	9	10
1	1.99	0.00	0.00	0.00	0.00	0.00	0.00	0.00	0.00	0.00
2	0.00	1.99	0.00	0.00	0.00	0.00	0.00	0.00	0.00	0.00
3	0.00	0.00	1.94	0.00	0.00	0.00	0.00	0.00	0.00	0.00
4	0.00	0.00	0.00	1.93	0.00	0.00	0.00	0.00	0.00	0.00
5	0.00	0.00	0.00	0.00	1.65	0.00	0.00	0.00	0.00	0.00
6	0.00	0.00	0.00	0.00	0.00	0.35	0.00	0.00	0.00	0.00
7	0.00	0.00	0.00	0.00	0.00	0.00	0.07	0.00	0.00	0.00
8	0.00	0.00	0.00	0.00	0.00	0.00	0.00	0.05	0.00	0.00
9	0.00	0.00	0.00	0.00	0.00	0.00	0.00	0.00	0.01	0.00
10	0.00	0.00	0.00	0.00	0.00	0.00	0.00	0.00	0.00	0.01

GeSn

ROOT 0: E= -4427.4214671097 Eh

0.76786 [0]: 2222200000
0.15129 [6]: 2222020000
0.01595 [217]: 2211201100
0.01076 [27]: 2221111000
0.00849 [168]: 2212110100
0.00732 [76]: 2220202000
0.00398 [432]: 2202202000
0.00345 [257]: 2211021100
0.00309 [80]: 2220200200
0.00283 [224]: 2211200011
0.00271 [436]: 2202200200

Table S3. CAS(2,2)-SCF and CAS(10,10)-SCF orbital occupations and configuration coefficients of **1-E**/**1-EE'**, corresponding to their ground state (S_0). All data was computed at the CAS(n,m)-SCF/def2TZVPP // PCM(benzene)-(u)B3LYP-D3/def2-SVP level of theory.

	n_{HONO}	n_{LUNO}	C_1	C_2	β
Si(2,2)	1.85	0.15	0.92	0.08	0.15
Si(10,10)	1.85	0.16	0.86	0.05	0.10
Ge(2,2)	1.76	0.24	0.88	0.12	0.24
Ge(10,10)	1.78	0.23	0.83	0.09	0.18
Sn(2,2)	1.45	0.55	0.73	0.27	0.55
Sn(10,10)	1.45	0.55	0.67	0.25	0.50
SiGe(2,2)	1.81	0.19	0.90	0.10	0.19
SiGe(10,10)	1.81	0.19	0.84	0.07	0.14
SiSn(2,2)	1.71	0.29	0.85	0.15	0.29
SiSn(10,10)	1.73	0.28	0.80	0.11	0.23
GeSn(2,2)	1.62	0.38	0.81	0.19	0.38
GeSn(10,10)	1.65	0.35	0.77	0.15	0.30

3. ASM and EDA—Dihydrogen activation

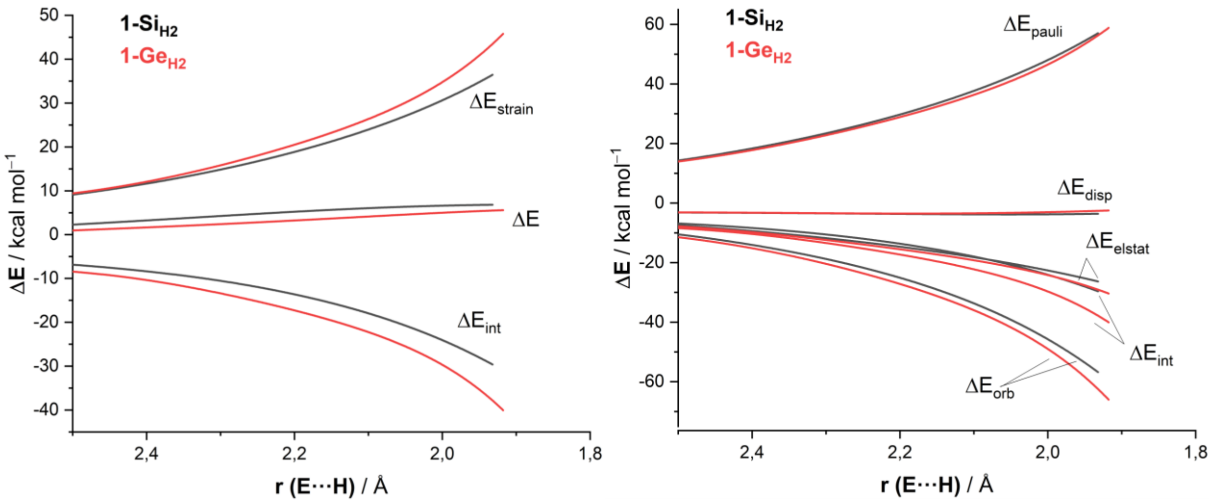

Figure S1. Comparative ASDs (left) and EDAs (right) for the reactions of dihydrogen with **1-E** (E = Si, Ge) projected onto the shortest C···E bond-forming distance. All data have been computed at the ZORA-B3LYP-D3/TZ2P//PCM(benzene)-B3LYP-D3/def2-SVP level.

4. Cartesian coordinates of the DFT optimized structures

Cartesian coordinates (in Å) and total energies (in a.u., noncorrected ZVPE included) of all the stationary points discussed in the text. Unless otherwise mentioned, all calculations have been performed at the PCM(benzene)-B3LYP-D3/def2-SVP level.
G_{qh} denotes free energy values computed at the PCM(benzene)-B3LYP-D3/def2-SVP with quasi-harmonic and concentration corrections, used during the DFT benchmark.
SP denotes values computed at the CPCM(benzene)-DLPNO-CCSD(T)/def2-TZVPP//PCM(benzene)-B3LYP-D3/def2-SVP.
G_{sp} denotes free energy values computed at the CPCM(benzene)-DLPNO-CCSD(T)/def2-TZVPP//PCM(benzene)-B3LYP-D3/def2-SVP with quasi-harmonic and concentration corrections.

1-Si_{CS} (B3LYP)
E **=** -2728.619154
H **=** -2728.567566
G **=** -2728.703422
G_{qh} **=** -2728.684110
N_{imag} **=** 0
SP **=** -2726.205884
G_{sp} **=** -2725.445195

Si	-0.000010000	1.990491000	0.116045000
N	2.735054000	1.086411000	0.001324000
N	2.735097000	-1.086447000	-0.001360000
C	3.527881000	-0.000003000	-0.000037000
C	1.360276000	0.712808000	0.020005000
C	1.360294000	-0.712888000	-0.020063000
C	3.215843000	2.438793000	0.081714000
C	3.250739000	3.221873000	-1.087058000
C	3.738170000	4.531035000	-0.974801000
H	3.779893000	5.161247000	-1.866930000
C	4.163787000	5.034759000	0.255940000
H	4.539027000	6.059097000	0.323504000
C	4.106513000	4.238903000	1.401476000
H	4.430988000	4.641251000	2.364571000
C	3.627027000	2.923997000	1.336213000
C	3.215989000	-2.438803000	-0.081573000
C	3.627226000	-2.924117000	-1.336004000
C	4.106964000	-4.238944000	-1.401087000
H	4.431521000	-4.641367000	-2.364123000
C	4.164447000	-5.034608000	-0.255435000
H	4.539899000	-6.058878000	-0.322861000
C	3.738788000	-4.530775000	0.975251000
H	3.780653000	-5.160848000	1.867471000
C	3.251100000	-3.221700000	1.087334000
Si	0.000004000	-1.990578000	-0.116208000
N	-2.735090000	-1.086464000	-0.001396000
N	-2.735068000	1.086395000	0.001212000
C	-3.527887000	-0.000028000	-0.000108000
C	-1.360292000	-0.712892000	-0.020130000
C	-1.360288000	0.712803000	0.019946000

C	-3.215959000	-2.438830000	-0.081551000
C	-3.250957000	-3.221694000	1.087378000
C	-3.738584000	-4.530799000	0.975364000
H	-3.780359000	-5.160848000	1.867605000
C	-4.164292000	-5.034688000	-0.255281000
H	-4.539697000	-6.058979000	-0.322655000
C	-4.106917000	-4.239055000	-1.400961000
H	-4.431508000	-4.641524000	-2.363967000
C	-3.627241000	-2.924202000	-1.335947000
C	-3.215877000	2.438770000	0.081596000
C	-3.627133000	2.923934000	1.336084000
C	-4.106664000	4.238823000	1.401356000
H	-4.431199000	4.641138000	2.364444000
C	-4.163912000	5.034702000	0.255835000
H	-4.539191000	6.059026000	0.323402000
C	-3.738225000	4.531017000	-0.974899000
H	-3.779929000	5.161248000	-1.867015000
C	-3.250747000	3.221874000	-1.087163000
C	2.763221000	2.678997000	-2.404793000
H	1.661358000	2.617780000	-2.401218000
H	3.076195000	3.321175000	-3.240258000
H	3.127097000	1.656328000	-2.592639000
C	3.549029000	2.047310000	2.559671000
H	3.716941000	2.630619000	3.475844000
H	2.563125000	1.561063000	2.630757000
H	4.308284000	1.247700000	2.525151000
C	-2.763168000	2.679039000	-2.404894000
H	-3.127081000	1.656396000	-2.592813000
H	-3.076067000	3.321270000	-3.240346000
H	-1.661309000	2.617779000	-2.401263000
C	-3.549185000	2.047212000	2.559521000
H	-3.717131000	2.630495000	3.475704000
H	-4.308445000	1.247608000	2.524947000
H	-2.563289000	1.560956000	2.630632000
C	3.548999000	-2.047610000	-2.559578000
H	3.717464000	-2.630918000	-3.475649000
H	2.562801000	-1.561994000	-2.630913000
H	4.307733000	-1.247512000	-2.524994000
C	2.763578000	-2.678675000	2.405007000
H	3.128218000	-1.656323000	2.593151000
H	1.661763000	-2.616554000	2.401062000
H	3.075778000	-3.321249000	3.240444000
C	-3.549117000	-2.047735000	-2.559555000
H	-3.717616000	-2.631082000	-3.475596000
H	-4.307878000	-1.247663000	-2.524956000
H	-2.562941000	-1.562085000	-2.630965000
C	-2.763363000	-2.678600000	2.404997000
H	-1.661553000	-2.616395000	2.400953000
H	-3.128061000	-1.656269000	2.593142000
H	-3.075445000	-3.321173000	3.240479000
C	5.001547000	0.000033000	-0.000157000
C	5.716009000	0.796234000	-0.914291000
C	5.716217000	-0.796168000	0.913817000
C	7.111598000	0.794444000	-0.912273000
C	7.111805000	-0.794327000	0.911524000
C	7.813785000	0.000075000	-0.000440000
H	5.180061000	1.411704000	-1.635032000
H	5.180435000	-1.411688000	1.634637000

H	7.651725000	1.415958000	-1.630242000
H	7.652098000	-1.415834000	1.629374000
H	8.906546000	0.000091000	-0.000549000
C	-5.001552000	-0.000009000	-0.000167000
C	-5.716168000	-0.796171000	0.913883000
C	-5.716071000	0.796134000	-0.914307000
C	-7.111756000	-0.794350000	0.911659000
C	-7.111660000	0.794324000	-0.912221000
C	-7.813792000	-0.000006000	-0.000311000
H	-5.180344000	-1.411645000	1.634712000
H	-5.180170000	1.411571000	-1.635110000
H	-7.652005000	-1.415828000	1.629569000
H	-7.651830000	1.415792000	-1.630197000
H	-8.906553000	-0.000005000	-0.000368000

1-Si$_{CS}$ (M062X)
***E* =** -2727.504062
***H* =** -2727.453592
***G* =** -2727.585770
N_{imag} = 0
G_{qh} = -2727.573276

Si	0.000000000	1.992377000	0.106149000
N	2.716281000	1.079824000	0.000082000
N	2.717793000	-1.082086000	0.017211000
C	3.504093000	-0.000552000	0.001993000
C	1.346287000	0.708732000	0.028218000
C	1.346717000	-0.712900000	0.008976000
C	3.185838000	2.431797000	0.084748000
C	3.188246000	3.224100000	-1.071669000
C	3.647849000	4.538192000	-0.950901000
H	3.665341000	5.176844000	-1.836037000
C	4.075466000	5.035630000	0.278118000
H	4.427571000	6.065568000	0.353330000
C	4.050323000	4.227530000	1.411972000
H	4.375818000	4.623635000	2.375623000
C	3.599682000	2.907132000	1.336014000
C	3.188991000	-2.433133000	-0.073819000
C	3.584727000	-2.910765000	-1.329642000
C	4.037358000	-4.230572000	-1.408160000
H	4.349260000	-4.628927000	-2.375376000
C	4.080839000	-5.035303000	-0.272583000
H	4.433578000	-6.064845000	-0.350233000
C	3.669732000	-4.535556000	0.961400000
H	3.700158000	-5.171905000	1.847793000
C	3.210088000	-3.221995000	1.084676000
Si	0.000001000	-1.998006000	-0.048052000
N	-2.717793000	-1.082087000	0.017213000
N	-2.716280000	1.079824000	0.000084000
C	-3.504093000	-0.000553000	0.001995000
C	-1.346716000	-0.712900000	0.008976000
C	-1.346286000	0.708732000	0.028219000
C	-3.188990000	-2.433134000	-0.073817000
C	-3.210088000	-3.221995000	1.084678000
C	-3.669731000	-4.535556000	0.961403000
H	-3.700156000	-5.171904000	1.847796000
C	-4.080837000	-5.035303000	-0.272580000
H	-4.433576000	-6.064846000	-0.350229000

C	-4.037356000	-4.230573000	-1.408158000
H	-4.349258000	-4.628929000	-2.375373000
C	-3.584726000	-2.910766000	-1.329640000
C	-3.185838000	2.431796000	0.084750000
C	-3.599682000	2.907131000	1.336016000
C	-4.050326000	4.227529000	1.411973000
H	-4.375820000	4.623634000	2.375624000
C	-4.075471000	5.035628000	0.278119000
H	-4.427579000	6.065565000	0.353330000
C	-3.647854000	4.538189000	-0.950900000
H	-3.665348000	5.176841000	-1.836036000
C	-3.188249000	3.224098000	-1.071667000
C	2.705020000	2.680275000	-2.387761000
H	1.604216000	2.628120000	-2.389853000
H	3.029294000	3.321335000	-3.217107000
H	3.067816000	1.656216000	-2.565681000
C	3.549842000	2.012969000	2.545037000
H	3.693253000	2.594704000	3.463781000
H	2.580527000	1.494631000	2.602595000
H	4.339736000	1.245381000	2.501891000

1-Si (wB97XD)
***E* =** -2727.784427
***H* =** -2727.733438
***G* =** -2727.867503
N_{imag} = 0
G_{qh} = -2727.854552

Si	0.000004000	1.976621000	0.104473000
N	2.719229000	1.081209000	0.007344000
N	2.719239000	-1.081244000	-0.007196000
C	3.503169000	-0.000011000	0.000050000
C	1.349422000	0.710135000	0.020348000
C	1.349424000	-0.710177000	-0.020130000
C	3.201884000	2.427616000	0.082983000
C	3.238277000	3.201665000	-1.085644000
C	3.726925000	4.506296000	-0.979697000
H	3.770637000	5.132489000	-1.873948000
C	4.150617000	5.013128000	0.245885000
H	4.527270000	6.036315000	0.309259000
C	4.089911000	4.225388000	1.391980000
H	4.412698000	4.632162000	2.353178000
C	3.609238000	2.915193000	1.332132000
C	3.201927000	-2.427642000	-0.082835000
C	3.609148000	-2.915264000	-1.332002000
C	4.089937000	-4.225422000	-1.391824000
H	4.412623000	-4.632233000	-2.353040000
C	4.150883000	-5.013073000	-0.245684000
H	4.527621000	-6.036230000	-0.309041000
C	3.727308000	-4.506199000	0.979925000
H	3.771179000	-5.132336000	1.874208000
C	3.238543000	-3.201614000	1.085846000
Si	0.000009000	-1.976667000	-0.104240000
N	-2.719224000	-1.081248000	-0.007319000
N	-2.719222000	1.081205000	0.007401000
C	-3.503156000	-0.000019000	0.000027000
C	-1.349409000	-0.710178000	-0.020169000
C	-1.349412000	0.710135000	0.020313000

C	-3.201912000	-2.427641000	-0.083073000
C	-3.238528000	-3.201707000	1.085544000
C	-3.727300000	-4.506281000	0.979520000
H	-3.771168000	-5.132491000	1.873752000
C	-4.150881000	-5.013054000	-0.246129000
H	-4.527623000	-6.036205000	-0.309567000
C	-4.089929000	-4.225313000	-1.392206000
H	-4.412617000	-4.632046000	-2.353455000
C	-3.609134000	-2.915162000	-1.332280000
C	-3.201891000	2.427598000	0.083202000
C	-3.609179000	2.915035000	1.332428000
C	-4.089893000	4.225207000	1.392443000
H	-4.412629000	4.631872000	2.353705000
C	-4.150707000	5.013062000	0.246432000
H	-4.527392000	6.036229000	0.309936000
C	-3.727083000	4.506369000	-0.979230000
H	-3.770879000	5.132651000	-1.873414000
C	-3.238392000	3.201767000	-1.085344000
C	2.751310000	2.655581000	-2.399130000
H	1.649545000	2.622966000	-2.405441000
H	3.088119000	3.281766000	-3.235962000
H	3.093011000	1.623629000	-2.572431000
C	3.526146000	2.047553000	2.558274000
H	3.674065000	2.642212000	3.469177000
H	2.545448000	1.551660000	2.621622000
H	4.298649000	1.261493000	2.541195000
C	-2.751500000	2.655842000	-2.398923000
H	-3.093152000	1.623886000	-2.572300000
H	-3.088413000	3.282091000	-3.235665000
H	-1.649733000	2.623299000	-2.405323000
C	-3.525974000	2.047273000	2.558475000
H	-3.673852000	2.641834000	3.469450000
H	-4.298450000	1.261187000	2.541369000
H	-2.545253000	1.551409000	2.621706000
C	3.525762000	-2.047717000	-2.558187000
H	3.674027000	-2.642341000	-3.469056000
H	2.544831000	-1.552290000	-2.621616000
H	4.297888000	-1.261289000	-2.541085000
C	2.751732000	-2.655427000	2.399348000
H	3.094694000	-1.623976000	2.573189000
H	1.650009000	-2.621444000	2.405282000
H	3.087456000	-3.282342000	3.236065000
C	-3.525739000	-2.047522000	-2.558398000
H	-3.674006000	-2.642077000	-3.469313000
H	-4.297861000	-1.261091000	-2.541240000
H	-2.544805000	-1.552096000	-2.621789000
C	-2.751688000	-2.655633000	2.399080000
H	-1.649965000	-2.621664000	2.404990000
H	-3.094636000	-1.624192000	2.573012000
H	-3.087400000	-3.282613000	3.235754000
C	4.978441000	0.000014000	-0.000132000
C	5.686472000	0.766703000	-0.935046000
C	5.686789000	-0.766655000	0.934555000
C	7.078391000	0.764814000	-0.933607000
C	7.078709000	-0.764702000	0.932692000
C	7.777136000	0.000075000	-0.000560000
H	5.148983000	1.362817000	-1.672322000
H	5.149557000	-1.362823000	1.671972000

H	7.618637000	1.364493000	-1.668446000
H	7.619203000	-1.364367000	1.667359000
H	8.869101000	0.000103000	-0.000723000
C	-4.978428000	-0.000002000	-0.000127000
C	-5.686750000	-0.766778000	0.934491000
C	-5.686481000	0.766782000	-0.934946000
C	-7.078670000	-0.764836000	0.932655000
C	-7.078400000	0.764881000	-0.933479000
C	-7.777121000	0.000037000	-0.000501000
H	-5.149498000	-1.363022000	1.671832000
H	-5.149009000	1.362980000	-1.672167000
H	-7.619146000	-1.364584000	1.667268000
H	-7.618664000	1.364635000	-1.668243000
H	-8.869086000	0.000057000	-0.000643000

1-Ge$_{CS}$ (B3LYP-D3)
***E* =** -6303.386108
***H* =** -6303.333043
***G* =** -6303.474166
***G_{qh}* =** -6303.459505
***N_{imag}* =** 0
***SP* =** -6299.339841
***G_{sp}* =** *-6298.584764*

Ge	0.000027000	2.069591000	0.121674000
N	2.786775000	1.085184000	-0.004786000
N	2.786730000	-1.085292000	0.004983000
C	3.582788000	-0.000069000	0.000131000
C	1.414930000	0.709882000	0.016074000
C	1.414904000	-0.709933000	-0.015971000
C	3.264488000	2.438714000	0.069032000
C	3.300369000	3.215768000	-1.103704000
C	3.778458000	4.528794000	-0.995687000
H	3.821083000	5.154525000	-1.890919000
C	4.193973000	5.041818000	0.234698000
H	4.561638000	6.069111000	0.298878000
C	4.136849000	4.251323000	1.383934000
H	4.454322000	4.660571000	2.346447000
C	3.666736000	2.932746000	1.322916000
C	3.264370000	-2.438847000	-0.068845000
C	3.667062000	-2.932732000	-1.322640000
C	4.137109000	-4.251333000	-1.383657000
H	4.454943000	-4.660465000	-2.346100000
C	4.193718000	-5.041995000	-0.234509000
H	4.561331000	-6.069306000	-0.298689000
C	3.777735000	-4.529121000	0.995780000
H	3.819925000	-5.154993000	1.890934000
C	3.299699000	-3.216075000	1.103791000
Ge	-0.000036000	-2.069600000	-0.121598000
N	-2.786783000	-1.085190000	0.004819000
N	-2.786738000	1.085284000	-0.004942000
C	-3.582797000	0.000062000	-0.000104000
C	-1.414938000	-0.709888000	-0.016022000
C	-1.414913000	0.709926000	0.016031000
C	-3.264486000	-2.438723000	-0.069015000
C	-3.300235000	-3.215828000	1.103690000
C	-3.778322000	-4.528854000	0.995666000
H	-3.820841000	-5.154627000	1.890874000

C	-4.193956000	-5.041829000	-0.234699000
H	-4.561616000	-6.069122000	-0.298887000
C	-4.136950000	-4.251285000	-1.383908000
H	-4.454515000	-4.660495000	-2.346406000
C	-3.666843000	-2.932707000	-1.322881000
C	-3.264375000	2.438840000	0.068883000
C	-3.667119000	2.932708000	1.322669000
C	-4.137157000	4.251311000	1.383689000
H	-4.455032000	4.660429000	2.346124000
C	-4.193708000	5.041994000	0.234552000
H	-4.561314000	6.069307000	0.298734000
C	-3.777674000	4.529138000	-0.995727000
H	-3.819819000	5.155026000	-1.890872000
C	-3.299647000	3.216089000	-1.103740000
C	2.826170000	2.662397000	-2.422316000
H	1.724781000	2.598184000	-2.430997000
H	3.145978000	3.299538000	-3.259040000
H	3.194380000	1.639460000	-2.599414000
C	3.591673000	2.060147000	2.549548000
H	3.745433000	2.649266000	3.464477000
H	2.612266000	1.560370000	2.615813000
H	4.362169000	1.270983000	2.522809000
C	-2.824784000	2.662922000	-2.422200000
H	-3.192956000	1.640038000	-2.599679000
H	-3.144108000	3.300230000	-3.258981000
H	-1.723394000	2.598644000	-2.430308000
C	-3.592707000	2.059885000	2.549182000
H	-3.746853000	2.648852000	3.464143000
H	-4.363255000	1.270787000	2.521933000
H	-2.613372000	1.560016000	2.615820000
C	3.592581000	-2.059933000	-2.549165000
H	3.746726000	-2.648909000	-3.464121000
H	2.613224000	-1.560106000	-2.615784000
H	4.363097000	-1.270803000	-2.521950000
C	2.824903000	-2.662879000	2.422262000
H	3.193215000	-1.640048000	2.599768000
H	1.723523000	-2.598454000	2.430372000
H	3.144143000	-3.300248000	3.259029000
C	-3.591917000	-2.060047000	-2.549478000
H	-3.745798000	-2.649116000	-3.464418000
H	-4.362396000	-1.270872000	-2.522601000
H	-2.612509000	-1.560282000	-2.615836000
C	-2.825882000	-2.662507000	2.422268000
H	-1.724501000	-2.598138000	2.430765000
H	-3.194207000	-1.639637000	2.599522000
H	-3.145455000	-3.299763000	3.258993000
C	5.056040000	-0.000074000	0.000080000
C	5.771272000	0.796309000	-0.913663000
C	5.771378000	-0.796435000	0.913760000
C	7.166854000	0.794756000	-0.911371000
C	7.166960000	-0.794847000	0.911333000
C	7.869213000	-0.000038000	-0.000053000
H	5.235838000	1.411882000	-1.634612000
H	5.236036000	-1.412012000	1.634769000
H	7.706924000	1.416652000	-1.629084000
H	7.707114000	-1.416726000	1.628997000
H	8.961973000	-0.000024000	-0.000105000
C	-5.056050000	0.000080000	-0.000140000

C	-5.771358000	-0.796275000	0.913568000
C	-5.771315000	0.796436000	-0.913882000
C	-7.166940000	-0.794700000	0.911181000
C	-7.166897000	0.794868000	-0.911552000
C	-7.869225000	0.000087000	-0.000199000
H	-5.235987000	-1.411842000	1.634567000
H	-5.235914000	1.411991000	-1.634866000
H	-7.707069000	-1.416575000	1.628868000
H	-7.706993000	1.416743000	-1.629264000
H	-8.961985000	0.000090000	-0.000222000

1-Ge$_{CS}$ (M062X)
***E* =** -6302.249149
***H* =** -6302.196385
***G* =** -6302.336548
***N*$_{imag}$ =** 0
***G*$_{qh}$ =** -6302.322018

Ge	-0.000005000	2.062493000	-0.091517000
N	-2.771446000	1.079942000	0.009979000
N	-2.771479000	-1.080001000	-0.010381000
C	-3.561242000	-0.000015000	0.000003000
C	-1.405057000	0.707864000	-0.009529000
C	-1.405068000	-0.707963000	0.008701000
C	-3.236386000	2.433780000	-0.070074000
C	-3.242480000	3.218560000	1.091437000
C	-3.689067000	4.537480000	0.974223000
H	-3.709119000	5.171072000	1.862901000
C	-4.100526000	5.045906000	-0.255813000
H	-4.441984000	6.079613000	-0.328187000
C	-4.073335000	4.244181000	-1.394136000
H	-4.387043000	4.648837000	-2.358110000
C	-3.635587000	2.919184000	-1.322105000
C	-3.236465000	-2.433824000	0.069760000
C	-3.635023000	-2.919371000	1.321921000
C	-4.072941000	-4.244319000	1.393967000
H	-4.386176000	-4.649094000	2.358043000
C	-4.100913000	-5.045832000	0.255520000
H	-4.442501000	-6.079495000	0.327903000
C	-3.690075000	-4.537251000	-0.974668000
H	-3.710733000	-5.170682000	-1.863446000
C	-3.243365000	-3.218380000	-1.091901000
Ge	-0.000005000	-2.062629000	0.090432000
N	2.771471000	-1.080000000	-0.010288000
N	2.771440000	1.079945000	0.009931000
C	3.561234000	-0.000014000	0.000001000
C	1.405058000	-0.707962000	0.008721000
C	1.405049000	0.707866000	-0.009506000
C	3.236460000	-2.433817000	0.069949000
C	3.243384000	-3.218436000	-1.091668000
C	3.690107000	-4.537296000	-0.974359000
H	3.710780000	-5.170776000	-1.863101000
C	4.100930000	-5.045808000	0.255863000
H	4.442525000	-6.079465000	0.328306000
C	4.072929000	-4.244235000	1.394267000
H	4.386150000	-4.648958000	2.358371000
C	3.635000000	-2.919295000	1.322144000
C	3.236386000	2.433775000	-0.070232000

C	3.635493000	2.919103000	-1.322323000
C	4.073258000	4.244087000	-1.394466000
H	4.386891000	4.648684000	-2.358488000
C	4.100560000	5.045876000	-0.256189000
H	4.442034000	6.079571000	-0.328649000
C	3.689203000	4.537523000	0.973911000
H	3.709350000	5.171162000	1.862554000
C	3.242601000	3.218617000	1.091238000
C	-2.778601000	2.661851000	2.409374000
H	-1.678545000	2.599044000	2.425269000
H	-3.106238000	3.300980000	3.238843000
H	-3.153695000	1.640511000	2.577021000
C	-3.586137000	2.030422000	-2.535171000
H	-3.714929000	2.618348000	-3.452107000
H	-2.622793000	1.500480000	-2.587901000
H	-4.385881000	1.272556000	-2.501839000
C	2.778855000	2.661987000	2.409253000
H	3.153946000	1.640648000	2.576913000
H	3.106597000	3.301152000	3.238653000
H	1.678800000	2.599207000	2.425271000
C	3.585927000	2.030275000	-2.535336000
H	3.714649000	2.618149000	-3.452314000
H	4.385660000	1.272398000	-2.502031000
H	2.622570000	1.500345000	-2.587955000
C	-3.584790000	-2.030797000	2.535092000
H	-3.713207000	-2.618838000	3.452006000
H	-2.621325000	-1.501034000	2.587408000
H	-4.384427000	-1.272794000	2.502277000
C	-2.780374000	-2.661360000	-2.410014000
H	-3.157232000	-1.640714000	-2.578017000
H	-1.680437000	-2.596693000	-2.425984000
H	-3.106971000	-3.301301000	-3.239260000
C	3.584729000	-2.030667000	2.535274000
H	3.713129000	-2.618665000	3.452218000
H	4.384357000	-1.272657000	2.502444000
H	2.621256000	-1.500912000	2.587542000
C	2.780388000	-2.661494000	-2.409811000
H	1.680451000	-2.596829000	-2.425777000
H	3.157246000	-1.640858000	-2.577876000
H	3.106981000	-3.301483000	-3.239021000
C	-5.035821000	0.000019000	0.000520000
C	-5.743293000	0.839453000	0.873228000
C	-5.743985000	-0.839393000	-0.871644000
C	-7.135714000	0.836354000	0.871677000
C	-7.136406000	-0.836254000	-0.869028000
C	-7.834580000	0.000059000	0.001590000
H	-5.203991000	1.490276000	1.561329000
H	-5.205245000	-1.490248000	-1.560150000
H	-7.675758000	1.490399000	1.557040000
H	-7.676992000	-1.490287000	-1.553975000
H	-8.925474000	0.000077000	0.002008000
C	5.035812000	0.000014000	0.000487000
C	5.743951000	-0.839504000	-0.871595000
C	5.743306000	0.839535000	0.873093000
C	7.136372000	-0.836377000	-0.869005000
C	7.135727000	0.836424000	0.871516000
C	7.834569000	0.000028000	0.001507000
H	5.205191000	-1.490436000	-1.560013000

H	5.204021000	1.490436000	1.561133000
H	7.676940000	-1.490493000	-1.553887000
H	7.675790000	1.490540000	1.556797000
H	8.925463000	0.000036000	0.001905000

1-Ge$_{OS}$ (M062X)
E **=** -6302.255901
H **=** -6302.202840
G **=** -6302.344038
$\boldsymbol{N_{imag}}$ **=** 0
$\boldsymbol{G_{qh}}$ **=** -6302.329291

Ge	-0.000001000	2.082339000	0.117868000
N	2.782048000	1.077857000	-0.008334000
N	2.782046000	-1.077870000	0.008333000
C	3.581310000	-0.000006000	0.000009000
C	1.426268000	0.701739000	0.008400000
C	1.426267000	-0.701750000	-0.008446000
C	3.239424000	2.435310000	0.066026000
C	3.242627000	3.212235000	-1.100443000
C	3.680074000	4.534785000	-0.990938000
H	3.697420000	5.163092000	-1.883387000
C	4.085782000	5.053263000	0.236870000
H	4.420257000	6.089613000	0.303625000
C	4.061633000	4.258505000	1.380165000
H	4.370766000	4.671321000	2.342124000
C	3.632975000	2.930099000	1.315778000
C	3.239429000	-2.435323000	-0.065994000
C	3.633038000	-2.930125000	-1.315720000
C	4.061726000	-4.258525000	-1.380064000
H	4.370909000	-4.671353000	-2.342003000
C	4.085845000	-5.053261000	-0.236754000
H	4.420343000	-6.089606000	-0.303476000
C	3.680071000	-4.534770000	0.991028000
H	3.697384000	-5.163062000	1.883487000
C	3.242594000	-3.212227000	1.100489000
Ge	-0.000002000	-2.082349000	-0.117948000
N	-2.782048000	-1.077869000	0.008333000
N	-2.782049000	1.077858000	-0.008330000
C	-3.581313000	-0.000005000	0.000011000
C	-1.426269000	-0.701749000	-0.008446000
C	-1.426270000	0.701739000	0.008403000
C	-3.239431000	-2.435322000	-0.065998000
C	-3.242573000	-3.212237000	1.100479000
C	-3.680050000	-4.534779000	0.991014000
H	-3.697345000	-5.163079000	1.883468000
C	-4.085845000	-5.053260000	-0.236765000
H	-4.420343000	-6.089605000	-0.303490000
C	-4.061747000	-4.258515000	-1.380069000
H	-4.370946000	-4.671335000	-2.342006000
C	-3.633060000	-2.930115000	-1.315722000
C	-3.239423000	2.435312000	0.066030000
C	-3.632996000	2.930093000	1.315778000
C	-4.061650000	4.258500000	1.380166000
H	-4.370802000	4.671311000	2.342122000
C	-4.085774000	5.053266000	0.236876000
H	-4.420246000	6.089618000	0.303632000
C	-3.680043000	4.534796000	-0.990927000

H	-3.697367000	5.163109000	-1.883371000
C	-3.242599000	3.212245000	-1.100433000
C	2.784850000	2.641589000	-2.414642000
H	3.093153000	3.286174000	-3.247204000
H	3.183887000	1.629230000	-2.581951000
H	1.686330000	2.553923000	-2.426126000
C	3.584437000	2.048699000	2.534062000
H	3.730175000	2.639639000	3.446455000
H	2.614612000	1.531889000	2.599449000
H	4.372644000	1.279142000	2.496029000
C	-2.784791000	2.641608000	-2.414625000
H	-3.183817000	1.629247000	-2.581947000
H	-3.093082000	3.286195000	-3.247190000
H	-1.686270000	2.553950000	-2.426087000
C	-3.584488000	2.048683000	2.534056000
H	-3.730245000	2.639615000	3.446450000
H	-4.372695000	1.279128000	2.495999000
H	-2.614665000	1.531870000	2.599460000
C	3.584541000	-2.048745000	-2.534020000
H	3.730369000	-2.639690000	-3.446395000
H	2.614695000	-1.531984000	-2.599479000
H	4.372707000	-1.279149000	-2.495943000
C	2.784752000	-2.641549000	2.414651000
H	3.184055000	-1.629316000	2.582104000
H	1.686256000	-2.553566000	2.425961000
H	3.092734000	-3.286280000	3.247218000
C	-3.584586000	-2.048723000	-2.534014000
H	-3.730432000	-2.639660000	-3.446392000
H	-4.372753000	-1.279128000	-2.495915000
H	-2.614742000	-1.531961000	-2.599487000
C	-2.784703000	-2.641569000	2.414636000
H	-1.686207000	-2.553585000	2.425921000
H	-3.184004000	-1.629340000	2.582107000
H	-3.092665000	-3.286309000	3.247204000
C	5.054095000	0.000000000	-0.000013000
C	5.764267000	0.852136000	-0.859493000
C	5.764319000	-0.852126000	0.859434000
C	7.156588000	0.850223000	-0.856190000
C	7.156640000	-0.850191000	0.856068000
C	7.855991000	0.000022000	-0.000077000
H	5.226501000	1.512299000	-1.539731000
H	5.226598000	-1.512301000	1.539694000
H	7.696428000	1.515391000	-1.531031000
H	7.696521000	-1.515351000	1.530883000
H	8.946862000	0.000031000	-0.000101000
C	-5.054097000	0.000001000	-0.000012000
C	-5.764323000	-0.852119000	0.859438000
C	-5.764269000	0.852133000	-0.859498000
C	-7.156644000	-0.850183000	0.856071000
C	-7.156590000	0.850220000	-0.856196000
C	-7.855994000	0.000025000	-0.000078000
H	-5.226603000	-1.512291000	1.539703000
H	-5.226503000	1.512292000	-1.539739000
H	-7.696525000	-1.515340000	1.530890000
H	-7.696429000	1.515385000	-1.531041000
H	-8.946865000	0.000034000	-0.000104000

1-Ge$_{CS}$ (wB97XD)
***E* =** -6302.629703
***H* =** -6302.577271
***G* =** -6302.716666
***N*$_{imag}$ =** 0
***G*$_{qh}$ =** -6302.702317

Ge	0.000032000	2.047820000	0.111160000
N	2.764346000	1.080110000	0.002648000
N	2.764325000	-1.080192000	-0.002474000
C	3.550917000	-0.000047000	0.000095000
C	1.396855000	0.707801000	0.017922000
C	1.396842000	-0.707859000	-0.017764000
C	3.243155000	2.427732000	0.071869000
C	3.277023000	3.196732000	-1.100217000
C	3.755667000	4.505433000	-0.998700000
H	3.797410000	5.127957000	-1.895610000
C	4.171829000	5.020935000	0.225886000
H	4.540299000	6.047311000	0.285734000
C	4.114246000	4.237796000	1.375290000
H	4.431539000	4.651198000	2.335490000
C	3.643766000	2.923661000	1.319848000
C	3.243122000	-2.427818000	-0.071710000
C	3.643903000	-2.923682000	-1.319656000
C	4.114410000	-4.237810000	-1.375094000
H	4.431845000	-4.651161000	-2.335269000
C	4.171843000	-5.021001000	-0.225720000
H	4.540333000	-6.047371000	-0.285564000
C	3.755494000	-4.505567000	0.998833000
H	3.797099000	-5.128140000	1.895715000
C	3.276821000	-3.196877000	1.100344000
Ge	-0.000009000	-2.047853000	-0.111007000
N	-2.764334000	-1.080128000	-0.002580000
N	-2.764298000	1.080175000	0.002533000
C	-3.550894000	0.000037000	-0.000073000
C	-1.396838000	-0.707831000	-0.017780000
C	-1.396819000	0.707830000	0.017879000
C	-3.243187000	-2.427736000	-0.071812000
C	-3.277525000	-3.196588000	1.100361000
C	-3.756242000	-4.505261000	0.998841000
H	-3.798354000	-5.127667000	1.895815000
C	-4.172025000	-5.020880000	-0.225826000
H	-4.540560000	-6.047233000	-0.285672000
C	-4.113990000	-4.237887000	-1.375304000
H	-4.430984000	-4.651382000	-2.335563000
C	-3.643426000	-2.923780000	-1.319863000
C	-3.243080000	2.427807000	0.071751000
C	-3.643765000	2.923715000	1.319714000
C	-4.114224000	4.237858000	1.375153000
H	-4.431573000	4.651245000	2.335341000
C	-4.171715000	5.021022000	0.225762000
H	-4.540170000	6.047404000	0.285607000
C	-3.755480000	4.505540000	-0.998807000
H	-3.797149000	5.128085000	-1.895706000
C	-3.276853000	3.196832000	-1.100321000
C	2.798292000	2.641256000	-2.413093000
H	1.696747000	2.605119000	-2.426668000
H	3.137962000	3.263373000	-3.251788000
H	3.143831000	1.609297000	-2.578041000

C	3.565166000	2.059521000	2.548796000
H	3.701254000	2.659047000	3.458346000
H	2.590516000	1.551380000	2.608828000
H	4.347520000	1.283116000	2.537639000
C	-2.798036000	2.641378000	-2.413174000
H	-3.143582000	1.609429000	-2.578172000
H	-3.137633000	3.263522000	-3.251880000
H	-1.696490000	2.605220000	-2.426669000
C	-3.565260000	2.059546000	2.548648000
H	-3.701403000	2.659054000	3.458202000
H	-4.347622000	1.283151000	2.537419000
H	-2.590620000	1.551392000	2.608735000
C	3.565486000	-2.059474000	-2.548567000
H	3.701761000	-2.658942000	-3.458128000
H	2.590827000	-1.551369000	-2.608744000
H	4.347808000	-1.283039000	-2.537222000
C	2.797866000	-2.641456000	2.413163000
H	3.143843000	-1.609697000	2.578462000
H	1.696336000	-2.604804000	2.426361000
H	3.136948000	-3.263906000	3.251848000
C	-3.564317000	-2.059808000	-2.548897000
H	-3.700173000	-2.659437000	-3.458414000
H	-4.346584000	-1.283312000	-2.538100000
H	-2.589587000	-1.551790000	-2.608671000
C	-2.799301000	-2.640938000	2.413348000
H	-1.697777000	-2.604335000	2.427181000
H	-3.145334000	-1.609133000	2.578247000
H	-3.138898000	-3.263213000	3.251955000
C	5.025838000	-0.000047000	0.000014000
C	5.734418000	0.766507000	-0.934804000
C	5.734557000	-0.766598000	0.934729000
C	7.126356000	0.764856000	-0.933068000
C	7.126495000	-0.764935000	0.932794000
C	7.825140000	-0.000037000	-0.000186000
H	5.197129000	1.362361000	-1.672410000
H	5.197381000	-1.362460000	1.672408000
H	7.666653000	1.364611000	-1.667843000
H	7.666902000	-1.364688000	1.667490000
H	8.917113000	-0.000033000	-0.000264000
C	-5.025811000	0.000051000	-0.000151000
C	-5.734510000	-0.766568000	0.934520000
C	-5.734403000	0.766662000	-0.934913000
C	-7.126448000	-0.764915000	0.932603000
C	-7.126341000	0.765004000	-0.933157000
C	-7.825108000	0.000045000	-0.000317000
H	-5.197313000	-1.362487000	1.672142000
H	-5.197126000	1.362567000	-1.672486000
H	-7.666843000	-1.364723000	1.667263000
H	-7.666652000	1.364805000	-1.667885000
H	-8.917082000	0.000043000	-0.000381000

1-Ge$_{OS}$ (wB97XD)
***E* =** -6302.630171
***H* =** -6302.577692
***G* =** -6302.717213
N_{imag} = 0
G_{qh} = -6302.702888

Ge	0.000005000	2.051545000	0.114754000
N	2.766330000	1.079737000	0.002965000
N	2.766326000	-1.079739000	-0.002958000
C	3.555061000	-0.000003000	0.000003000
C	1.401518000	0.706407000	0.017531000
C	1.401515000	-0.706405000	-0.017521000
C	3.243356000	2.428233000	0.071684000
C	3.274351000	3.196878000	-1.100641000
C	3.751144000	4.506304000	-1.000105000
H	3.790507000	5.128765000	-1.897151000
C	4.168417000	5.022555000	0.223815000
H	4.535446000	6.049479000	0.282967000
C	4.113685000	4.239551000	1.373457000
H	4.431776000	4.653608000	2.333098000
C	3.645012000	2.924714000	1.319009000
C	3.243355000	-2.428233000	-0.071672000
C	3.645093000	-2.924695000	-1.318978000
C	4.113768000	-4.239531000	-1.373415000
H	4.431925000	-4.653573000	-2.333042000
C	4.168419000	-5.022554000	-0.223782000
H	4.535447000	-6.049480000	-0.282925000
C	3.751060000	-4.506323000	1.000117000
H	3.790351000	-5.128802000	1.897154000
C	3.274267000	-3.196897000	1.100642000
Ge	0.000001000	-2.051544000	-0.114708000
N	-2.766324000	-1.079734000	-0.002945000
N	-2.766323000	1.079743000	0.002954000
C	-3.555056000	0.000005000	-0.000004000
C	-1.401512000	-0.706403000	-0.017511000
C	-1.401511000	0.706410000	0.017532000
C	-3.243361000	-2.428226000	-0.071648000
C	-3.274364000	-3.196856000	1.100687000
C	-3.751168000	-4.506278000	1.000166000
H	-3.790532000	-5.128730000	1.897219000
C	-4.168448000	-5.022540000	-0.223748000
H	-4.535487000	-6.049462000	-0.282885000
C	-4.113711000	-4.239549000	-1.373398000
H	-4.431810000	-4.653613000	-2.333035000
C	-3.645026000	-2.924717000	-1.318966000
C	-3.243347000	2.428240000	0.071655000
C	-3.644983000	2.924746000	1.318978000
C	-4.113652000	4.239584000	1.373407000
H	-4.431727000	4.653661000	2.333046000
C	-4.168400000	5.022567000	0.223752000
H	-4.535426000	6.049493000	0.282890000
C	-3.751148000	4.506291000	-1.000165000
H	-3.790526000	5.128735000	-1.897223000
C	-3.274360000	3.196863000	-1.100684000
C	2.794166000	2.639865000	-2.412292000
H	3.126822000	3.264928000	-3.251583000
H	3.145684000	1.610263000	-2.579616000
H	1.692690000	2.597300000	-2.421386000
C	3.568948000	2.060808000	2.548237000
H	3.708905000	2.660165000	3.457299000
H	2.593613000	1.554358000	2.611518000
H	4.349763000	1.282912000	2.534576000
C	-2.794207000	2.639820000	-2.412334000

H	-3.145738000	1.610218000	-2.579630000
H	-3.126877000	3.264869000	-3.251630000
H	-1.692732000	2.597245000	-2.421451000
C	-3.568898000	2.060865000	2.548222000
H	-3.708832000	2.660243000	3.457274000
H	-4.349717000	1.282973000	2.534595000
H	-2.593564000	1.554411000	2.611493000
C	3.569120000	-2.060765000	-2.548195000
H	3.709201000	-2.660097000	-3.457255000
H	2.593768000	-1.554359000	-2.611566000
H	4.349895000	-1.282832000	-2.534438000
C	2.793957000	-2.639908000	2.412258000
H	3.145537000	-1.610342000	2.579678000
H	1.692482000	-2.597254000	2.421209000
H	3.126453000	-3.265037000	3.251562000
C	-3.568957000	-2.060824000	-2.548203000
H	-3.708979000	-2.660181000	-3.457256000
H	-4.349724000	-1.282882000	-2.534524000
H	-2.593594000	-1.554430000	-2.611519000
C	-2.794159000	-2.639831000	2.412325000
H	-1.692686000	-2.597189000	2.421371000
H	-3.145744000	-1.610256000	2.579683000
H	-3.126735000	-3.264930000	3.251620000
C	5.029517000	-0.000009000	-0.000007000
C	5.738618000	0.772020000	-0.930170000
C	5.738627000	-0.772059000	0.930131000
C	7.130542000	0.770652000	-0.928094000
C	7.130551000	-0.770705000	0.928029000
C	7.829424000	-0.000028000	-0.000037000
H	5.201638000	1.372108000	-1.664573000
H	5.201654000	-1.372153000	1.664534000
H	7.670777000	1.375001000	-1.659154000
H	7.670794000	-1.375066000	1.659074000
H	8.921391000	-0.000034000	-0.000048000
C	-5.029511000	0.000002000	-0.000023000
C	-5.738627000	-0.772051000	0.930109000
C	-5.738607000	0.772036000	-0.930186000
C	-7.130550000	-0.770694000	0.928001000
C	-7.130530000	0.770670000	-0.928116000
C	-7.829417000	-0.000012000	-0.000065000
H	-5.201658000	-1.372149000	1.664512000
H	-5.201621000	1.372126000	-1.664584000
H	-7.670797000	-1.375057000	1.659041000
H	-7.670761000	1.375024000	-1.659176000
H	-8.921384000	-0.000016000	-0.000080000

1-Sn$_{CS}$ (B3LYP-D3)
E **=** -2578.548694
H **=** -2578.494673
G **=** -2578.639805
G$_{qh}$ **=** -2578.624092
N$_{imag}$ **=** 0

Sn	0.000009000	2.255563000	0.153812000
N	2.941219000	1.082640000	-0.013857000
N	2.941201000	-1.082667000	0.013883000
C	3.742728000	-0.000019000	0.000061000
C	1.571652000	0.706333000	0.009446000

C	1.571642000	-0.706337000	-0.009596000
C	3.417402000	2.437224000	0.047638000
C	3.457559000	3.202674000	-1.133140000
C	3.924676000	4.520577000	-1.034687000
H	3.969577000	5.137466000	-1.935897000
C	4.327882000	5.048976000	0.193149000
H	4.686392000	6.079923000	0.249678000
C	4.272927000	4.268232000	1.349137000
H	4.584574000	4.688263000	2.308869000
C	3.814184000	2.945208000	1.298411000
C	3.417365000	-2.437261000	-0.047551000
C	3.814340000	-2.945236000	-1.298265000
C	4.273071000	-4.268267000	-1.348932000
H	4.584869000	-4.688290000	-2.308618000
C	4.327826000	-5.049026000	-0.192945000
H	4.686329000	-6.079977000	-0.249428000
C	3.924424000	-4.520637000	1.034832000
H	3.969164000	-5.137539000	1.936041000
C	3.457311000	-3.202727000	1.133225000
Sn	-0.000005000	-2.255541000	-0.154196000
N	-2.941207000	-1.082644000	0.013848000
N	-2.941207000	1.082663000	-0.013930000
C	-3.742724000	0.000010000	-0.000004000
C	-1.571644000	-0.706326000	-0.009623000
C	-1.571644000	0.706344000	0.009420000
C	-3.417387000	-2.437234000	-0.047555000
C	-3.457464000	-3.202631000	1.133261000
C	-3.924588000	-4.520539000	1.034899000
H	-3.969431000	-5.137386000	1.936140000
C	-4.327876000	-5.048993000	-0.192888000
H	-4.686388000	-6.079943000	-0.249346000
C	-4.272997000	-4.268301000	-1.348914000
H	-4.584706000	-4.688375000	-2.308606000
C	-3.814253000	-2.945273000	-1.298278000
C	-3.417378000	2.437252000	0.047548000
C	-3.814235000	2.945221000	1.298302000
C	-4.272970000	4.268248000	1.349018000
H	-4.584676000	4.688267000	2.308736000
C	-4.327845000	5.049010000	0.193038000
H	-4.686350000	6.079959000	0.249559000
C	-3.924564000	4.520625000	-1.034780000
H	-3.969401000	5.137528000	-1.935984000
C	-3.457452000	3.202719000	-1.133222000
C	3.002791000	2.633939000	-2.452666000
H	1.900727000	2.594561000	-2.491206000
H	3.357381000	3.248430000	-3.292389000
H	3.351640000	1.600155000	-2.601612000
C	3.752434000	2.080943000	2.531769000
H	3.894783000	2.679741000	3.442214000
H	2.783034000	1.562898000	2.602340000
H	4.537830000	1.306353000	2.510624000
C	-3.002595000	2.634005000	-2.452727000
H	-3.351433000	1.600224000	-2.601715000
H	-3.357127000	3.248510000	-3.292464000
H	-1.900528000	2.594627000	-2.491192000
C	-3.752577000	2.080933000	2.531648000
H	-3.894985000	2.679715000	3.442095000
H	-4.537978000	1.306350000	2.510433000

H	-2.783187000	1.562879000	2.602277000
C	3.752804000	-2.080951000	-2.531618000
H	3.895333000	-2.679730000	-3.442048000
H	2.783407000	-1.562923000	-2.602359000
H	4.538181000	-1.306346000	-2.510313000
C	3.002329000	-2.633998000	2.452680000
H	3.351319000	-1.600281000	2.601771000
H	1.900263000	-2.594434000	2.490965000
H	3.356621000	-3.248597000	3.292448000
C	-3.752575000	-2.081065000	-2.531678000
H	-3.895017000	-2.679899000	-3.442086000
H	-4.537943000	-1.306448000	-2.510504000
H	-2.783163000	-1.563056000	-2.602350000
C	-3.002631000	-2.633824000	2.452734000
H	-1.900569000	-2.594264000	2.491145000
H	-3.351633000	-1.600096000	2.601720000
H	-3.357025000	-3.248369000	3.292499000
C	5.215478000	-0.000019000	0.000109000
C	5.931932000	0.790560000	-0.918240000
C	5.931893000	-0.790586000	0.918498000
C	7.327507000	0.789418000	-0.915551000
C	7.327468000	-0.789428000	0.915882000
C	8.030180000	-0.000001000	0.000184000
H	5.397253000	1.401474000	-1.643640000
H	5.397185000	-1.401504000	1.643873000
H	7.867393000	1.407235000	-1.636970000
H	7.867323000	-1.407236000	1.637331000
H	9.122929000	0.000005000	0.000213000
C	-5.215474000	0.000011000	0.000062000
C	-5.931869000	-0.790546000	0.918476000
C	-5.931949000	0.790568000	-0.918289000
C	-7.327444000	-0.789397000	0.915883000
C	-7.327523000	0.789418000	-0.915577000
C	-8.030176000	0.000010000	0.000183000
H	-5.397144000	-1.401451000	1.643850000
H	-5.397286000	1.401471000	-1.643711000
H	-7.867284000	-1.407197000	1.637351000
H	-7.867425000	1.407217000	-1.636999000
H	-9.122926000	0.000010000	0.000230000

1-Sn$_{OS}$ (B3LYP-D3)
***E* =** -2578.551811
***H* =** -2578.497614
***G* =** -2578.643446
G_{qh} = -2578.627547
N_{imag} = 0
***SP* =** -2575.450963
G_{sp} = -2574.700468

Sn	0.000015000	2.265923000	0.186166000
N	2.956208000	1.081718000	-0.016912000
N	2.956201000	-1.081728000	0.016907000
C	3.766145000	-0.000007000	0.000017000
C	1.595212000	0.702054000	0.005558000
C	1.595209000	-0.702056000	-0.005637000
C	3.422275000	2.440294000	0.040639000
C	3.446194000	3.203168000	-1.142099000
C	3.900336000	4.525953000	-1.050261000

H	3.931931000	5.141399000	-1.952994000
C	4.307663000	5.060672000	0.173574000
H	4.656150000	6.095282000	0.225459000
C	4.269328000	4.281831000	1.331534000
H	4.583937000	4.707091000	2.287969000
C	3.823416000	2.954082000	1.287295000
C	3.422265000	-2.440307000	-0.040596000
C	3.823539000	-2.954101000	-1.287204000
C	4.269468000	-4.281847000	-1.331383000
H	4.584186000	-4.707111000	-2.287781000
C	4.307685000	-5.060678000	-0.173414000
H	4.656186000	-6.095285000	-0.225253000
C	3.900215000	-4.525955000	1.050374000
H	3.931706000	-5.141398000	1.953112000
C	3.446053000	-3.203174000	1.142151000
Sn	0.000014000	-2.265921000	-0.186316000
N	-2.956180000	-1.081727000	0.016916000
N	-2.956186000	1.081720000	-0.016922000
C	-3.766118000	-0.000005000	0.000015000
C	-1.595186000	-0.702056000	-0.005620000
C	-1.595189000	0.702056000	0.005541000
C	-3.422264000	-2.440301000	-0.040572000
C	-3.446393000	-3.203044000	1.142249000
C	-3.900569000	-4.525821000	1.050487000
H	-3.932329000	-5.141166000	1.953283000
C	-4.307728000	-5.060660000	-0.173353000
H	-4.656245000	-6.095263000	-0.225177000
C	-4.269196000	-4.281946000	-1.331390000
H	-4.583674000	-4.707299000	-2.287827000
C	-3.823247000	-2.954206000	-1.287229000
C	-3.422271000	2.440291000	0.040615000
C	-3.823130000	2.954187000	1.287319000
C	-4.269057000	4.281931000	1.331541000
H	-4.583434000	4.707276000	2.288015000
C	-4.307694000	5.060661000	0.173517000
H	-4.656194000	6.095267000	0.225389000
C	-3.900668000	4.525831000	-1.050370000
H	-3.932521000	5.141183000	-1.953157000
C	-3.446517000	3.203048000	-1.142194000
C	2.988572000	2.622838000	-2.455497000
H	3.308636000	3.250600000	-3.299159000
H	3.370758000	1.601899000	-2.612398000
H	1.887849000	2.547125000	-2.477064000
C	3.776372000	2.092615000	2.523086000
H	3.935016000	2.692445000	3.430119000
H	2.805751000	1.579096000	2.609388000
H	4.556642000	1.313390000	2.491186000
C	-2.989289000	2.622580000	-2.455668000
H	-3.371505000	1.601616000	-2.612333000
H	-3.309628000	3.250242000	-3.299301000
H	-1.888571000	2.546888000	-2.477567000
C	-3.775737000	2.092852000	2.523188000
H	-3.934157000	2.692775000	3.430199000
H	-4.555994000	1.313603000	2.491581000
H	-2.805079000	1.579368000	2.609289000
C	3.776631000	-2.092637000	-2.523002000
H	3.935396000	-2.692466000	-3.430015000
H	2.806011000	-1.579137000	-2.609423000

H	4.556883000	-1.313398000	-2.491008000
C	2.988278000	-2.622816000	2.455483000
H	3.370805000	-1.602034000	2.612593000
H	1.887579000	-2.546695000	2.476761000
H	3.307886000	-3.250777000	3.299169000
C	-3.775974000	-2.092882000	-2.523111000
H	-3.934508000	-2.692808000	-3.430100000
H	-4.556210000	-1.313615000	-2.491421000
H	-2.805314000	-1.579421000	-2.609323000
C	-2.989025000	-2.622543000	2.455658000
H	-1.888333000	-2.546422000	2.477271000
H	-3.371601000	-1.601741000	2.612533000
H	-3.308899000	-3.250409000	3.299314000
C	5.236232000	-0.000004000	0.000009000
C	5.955638000	0.822278000	-0.890014000
C	5.955671000	-0.822277000	0.890012000
C	7.350910000	0.821953000	-0.886110000
C	7.350944000	-0.821935000	0.886070000
C	8.054739000	0.000013000	-0.000029000
H	5.422713000	1.457201000	-1.595674000
H	5.422775000	-1.457209000	1.595685000
H	7.890317000	1.465251000	-1.585402000
H	7.890376000	-1.465228000	1.585347000
H	9.147465000	0.000019000	-0.000044000
C	-5.236200000	-0.000001000	0.000005000
C	-5.955635000	-0.822335000	0.889953000
C	-5.955599000	0.822341000	-0.889965000
C	-7.350908000	-0.821994000	0.886007000
C	-7.350871000	0.822017000	-0.886062000
C	-8.054700000	0.000016000	-0.000037000
H	-5.422733000	-1.457320000	1.595578000
H	-5.422666000	1.457316000	-1.595576000
H	-7.890342000	-1.465336000	1.585238000
H	-7.890277000	1.465364000	-1.585310000
H	-9.147427000	0.000023000	-0.000054000

1-Sn$_{CS}$ (M062X)
***E* =** -2577.202212
***H* =** -2577.148480
***G* =** -2577.292003
G_{qh} = -2577.276896
N_{imag} = 0

Sn	-0.000002000	2.245154000	-0.108084000
N	-2.922753000	1.077053000	0.019877000
N	-2.922752000	-1.077070000	-0.019699000
C	-3.718365000	-0.000007000	0.000062000
C	-1.558839000	0.704550000	-0.001493000
C	-1.558839000	-0.704581000	0.001880000
C	-3.380814000	2.433509000	-0.052331000
C	-3.392569000	3.209998000	1.115258000
C	-3.814821000	4.537432000	1.001099000
H	-3.837532000	5.164865000	1.894050000
C	-4.200090000	5.061354000	-0.230658000
H	-4.521655000	6.101561000	-0.300543000
C	-4.174983000	4.265966000	-1.373571000
H	-4.472421000	4.681610000	-2.337992000
C	-3.761575000	2.933014000	-1.305434000

C	-3.380894000	-2.433523000	0.052098000
C	-3.761847000	-2.933377000	1.304986000
C	-4.175520000	-4.266281000	1.372619000
H	-4.473123000	-4.682206000	2.336868000
C	-4.200701000	-5.061253000	0.229427000
H	-4.522481000	-6.101420000	0.298924000
C	-3.815208000	-4.536984000	-1.002125000
H	-3.837941000	-5.164100000	-1.895295000
C	-3.392679000	-3.209603000	-1.115772000
Sn	-0.000002000	-2.245180000	0.108498000
N	2.922749000	-1.077071000	-0.019699000
N	2.922751000	1.077052000	0.019885000
C	3.718361000	-0.000008000	0.000062000
C	1.558836000	-0.704581000	0.001883000
C	1.558836000	0.704551000	-0.001483000
C	3.380893000	-2.433523000	0.052090000
C	3.392716000	-3.209584000	-1.115793000
C	3.815245000	-4.536966000	-1.002153000
H	3.838008000	-5.164068000	-1.895333000
C	4.200702000	-5.061254000	0.229402000
H	4.522482000	-6.101421000	0.298892000
C	4.175485000	-4.266300000	1.372606000
H	4.473061000	-4.682239000	2.336857000
C	3.761812000	-2.933395000	1.304981000
C	3.380815000	2.433508000	-0.052322000
C	3.761521000	2.933032000	-1.305434000
C	4.174932000	4.265983000	-1.373568000
H	4.472326000	4.681642000	-2.337996000
C	4.200095000	5.061352000	-0.230643000
H	4.521662000	6.101559000	-0.300526000
C	3.814883000	4.537411000	1.001123000
H	3.837643000	5.164827000	1.894085000
C	3.392631000	3.209977000	1.115281000
C	-2.963800000	2.637823000	2.439236000
H	-1.864153000	2.597523000	2.498534000
H	-3.333823000	3.256038000	3.266869000
H	-3.323205000	1.606673000	2.575637000
C	-3.725539000	2.048857000	-2.522464000
H	-3.836164000	2.643713000	-3.437259000
H	-2.775289000	1.495744000	-2.574072000
H	-4.543460000	1.310324000	-2.495165000
C	2.963939000	2.637778000	2.439272000
H	3.323352000	1.606625000	2.575633000
H	3.334011000	3.255976000	3.266895000
H	1.864295000	2.597477000	2.498636000
C	3.725420000	2.048898000	-2.522478000
H	3.836002000	2.643770000	-3.437268000
H	4.543338000	1.310359000	-2.495234000
H	2.775164000	1.495791000	-2.574050000
C	-3.725771000	-2.049621000	2.522305000
H	-3.836632000	-2.644742000	3.436900000
H	-2.775407000	-1.496734000	2.574208000
H	-4.543528000	-1.310901000	2.495134000
C	-2.963648000	-2.636910000	-2.439431000
H	-3.325390000	-1.606670000	-2.576646000
H	-1.864030000	-2.593964000	-2.497390000
H	-3.331209000	-3.256272000	-3.267290000
C	3.725696000	-2.049659000	2.522314000

H	3.836531000	-2.644795000	3.436902000
H	4.543452000	-1.310936000	2.495180000
H	2.775329000	-1.496776000	2.574196000
C	2.963734000	-2.636869000	-2.439457000
H	1.864118000	-2.593922000	-2.497456000
H	3.325481000	-1.606627000	-2.576641000
H	3.331326000	-3.256216000	-3.267313000
C	-5.192446000	-0.000014000	0.000208000
C	-5.901326000	0.838597000	0.873090000
C	-5.901575000	-0.838681000	-0.872410000
C	-7.293766000	0.835947000	0.870834000
C	-7.294016000	-0.836088000	-0.869696000
C	-7.992807000	-0.000081000	0.000678000
H	-5.363067000	1.488616000	1.562657000
H	-5.363533000	-1.488739000	-1.562110000
H	-7.833754000	1.489904000	1.556375000
H	-7.834200000	-1.490088000	-1.555041000
H	-9.083689000	-0.000108000	0.000861000
C	5.192442000	-0.000016000	0.000204000
C	5.901567000	-0.838688000	-0.872413000
C	5.901325000	0.838598000	0.873079000
C	7.294007000	-0.836096000	-0.869704000
C	7.293764000	0.835948000	0.870818000
C	7.992802000	-0.000085000	0.000664000
H	5.363520000	-1.488750000	-1.562107000
H	5.363067000	1.488621000	1.562645000
H	7.834188000	-1.490100000	-1.555047000
H	7.833755000	1.489908000	1.556354000
H	9.083683000	-0.000113000	0.000843000

1-Sn_{OS} (M062X)
***E* =** -2577.225691
***H* =** -2577.171585
***G* =** -2577.317466
G_{qh} = -2577.301343
N_{imag} = 0

Sn	-0.000003000	2.279421000	-0.188165000
N	-2.942175000	1.074298000	0.018613000
N	-2.942154000	-1.074340000	-0.018593000
C	-3.750145000	-0.000023000	-0.000098000
C	-1.592105000	0.696646000	0.000935000
C	-1.592093000	-0.696666000	-0.000640000
C	-3.386453000	2.436782000	-0.035392000
C	-3.373778000	3.195902000	1.143267000
C	-3.779719000	4.530028000	1.053820000
H	-3.782739000	5.145235000	1.955474000
C	-4.172699000	5.075898000	-0.166237000
H	-4.481582000	6.120981000	-0.217906000
C	-4.169658000	4.297226000	-1.320854000
H	-4.470956000	4.731197000	-2.275927000
C	-3.772776000	2.957970000	-1.277081000
C	-3.386447000	-2.436825000	0.035313000
C	-3.772929000	-2.958076000	1.276918000
C	-4.169909000	-4.297314000	1.320547000
H	-4.471334000	-4.731334000	2.275558000
C	-4.172888000	-5.075895000	0.165874000
H	-4.481853000	-6.120959000	0.217429000
C	-3.779729000	-4.529964000	-1.054104000
H	-3.782680000	-5.145105000	-1.955801000

C	-3.373675000	-3.195867000	-1.143402000
Sn	0.000000000	-2.279420000	0.188843000
N	2.942153000	-1.074335000	-0.018598000
N	2.942171000	1.074302000	0.018614000
C	3.750141000	-0.000018000	-0.000100000
C	1.592091000	-0.696663000	-0.000643000
C	1.592101000	0.696650000	0.000938000
C	3.386451000	-2.436819000	0.035295000
C	3.373723000	-3.195837000	-1.143435000
C	3.779783000	-4.529933000	-1.054151000
H	3.782769000	-5.145056000	-1.955861000
C	4.172907000	-5.075887000	0.165829000
H	4.481878000	-6.120949000	0.217372000
C	4.169888000	-4.297328000	1.320517000
H	4.471285000	-4.731365000	2.275529000
C	3.772901000	-2.958091000	1.276902000
C	3.386449000	2.436787000	-0.035385000
C	3.772735000	2.957992000	-1.277079000
C	4.169611000	4.297250000	-1.320846000
H	4.470878000	4.731234000	-2.275923000
C	4.172686000	5.075907000	-0.166219000
H	4.481564000	6.120991000	-0.217885000
C	3.779747000	4.530019000	1.053843000
H	3.782798000	5.145212000	1.955505000
C	3.373813000	3.195891000	1.143285000
C	-2.933707000	2.592912000	2.449672000
H	-3.229674000	3.231670000	3.291091000
H	-3.358235000	1.588215000	2.598689000
H	-1.837597000	2.478459000	2.468011000
C	-3.751144000	2.092872000	-2.507776000
H	-3.896900000	2.698565000	-3.410382000
H	-2.791297000	1.560179000	-2.591915000
H	-4.551014000	1.335457000	-2.470265000
C	2.933795000	2.592883000	2.449699000
H	3.358283000	1.588161000	2.598662000
H	3.229844000	3.231603000	3.291119000
H	1.837680000	2.478482000	2.468103000
C	3.751061000	2.092911000	-2.507786000
H	3.896808000	2.698615000	-3.410386000
H	4.550917000	1.335481000	-2.470302000
H	2.791203000	1.560238000	-2.591911000
C	-3.751377000	-2.093077000	2.507685000
H	-3.897243000	-2.698837000	3.410228000
H	-2.791517000	-1.560430000	2.591958000
H	-4.551215000	-1.335629000	2.470160000
C	-2.933494000	-2.592732000	-2.449699000
H	-3.359143000	-1.588579000	-2.599255000
H	-1.837515000	-2.476979000	-2.467415000
H	-3.228211000	-3.232054000	-3.291125000
C	3.751301000	-2.093117000	2.507686000
H	3.897156000	-2.698893000	3.410220000
H	4.551125000	-1.335653000	2.470196000
H	2.791429000	-1.560491000	2.591945000
C	2.933588000	-2.592676000	-2.449736000
H	1.837611000	-2.476905000	-2.467481000
H	3.359259000	-1.588529000	-2.599266000
H	3.228318000	-3.231991000	-3.291163000
C	-5.221746000	-0.000011000	-0.000161000
C	-5.933617000	0.859599000	0.851399000
C	-5.933615000	-0.859584000	-0.851758000
C	-7.325930000	0.858419000	0.847249000

C	-7.325929000	-0.858348000	-0.847664000
C	-8.025904000	0.000048000	-0.000222000
H	-5.396749000	1.525295000	1.526941000
H	-5.396754000	-1.525307000	-1.527277000
H	-7.865538000	1.530178000	1.515800000
H	-7.865536000	-1.530085000	-1.516238000
H	-9.116756000	0.000072000	-0.000247000
C	5.221742000	-0.000005000	-0.000158000
C	5.933614000	-0.859580000	-0.851749000
C	5.933608000	0.859604000	0.851407000
C	7.325928000	-0.858346000	-0.847647000
C	7.325920000	0.858424000	0.847264000
C	8.025898000	0.000052000	-0.000202000
H	5.396756000	-1.525305000	-1.527268000
H	5.396735000	1.525300000	1.526946000
H	7.865539000	-1.530085000	-1.516217000
H	7.865526000	1.530183000	1.515818000
H	9.116750000	0.000074000	-0.000221000

1-Sn$_{CS}$ (wB97XD)
***E* =** -2577.744660
***H* =** -2577.691372
***G* =** -2577.833567
***G$_{qh}$* =** *-2577.818763*
***N*$_{imag}$ =** 0

Sn	0.000002000	2.222936000	0.125429000
N	2.909915000	1.077622000	-0.007383000
N	2.909917000	-1.077635000	0.007434000
C	3.701359000	-0.000005000	0.000002000
C	1.545025000	0.704643000	0.010281000
C	1.545026000	-0.704659000	-0.010166000
C	3.384799000	2.426908000	0.052714000
C	3.426966000	3.185893000	-1.125888000
C	3.887810000	4.501546000	-1.028834000
H	3.934563000	5.116851000	-1.930441000
C	4.280810000	5.032527000	0.196533000
H	4.634552000	6.064250000	0.252618000
C	4.221028000	4.257327000	1.351316000
H	4.523265000	4.681848000	2.311483000
C	3.768588000	2.936820000	1.301045000
C	3.384809000	-2.426917000	-0.052684000
C	3.768542000	-2.936826000	-1.301032000
C	4.221006000	-4.257326000	-1.351320000
H	4.523203000	-4.681844000	-2.311501000
C	4.280864000	-5.032519000	-0.196537000
H	4.634624000	-6.064235000	-0.252635000
C	3.887915000	-4.501541000	1.028848000
H	3.934723000	-5.116841000	1.930455000
C	3.427049000	-3.185897000	1.125919000
Sn	-0.000001000	-2.222953000	-0.125250000
N	-2.909918000	-1.077632000	0.007432000
N	-2.909911000	1.077625000	-0.007367000
C	-3.701357000	-0.000001000	-0.000001000
C	-1.545024000	-0.704658000	-0.010147000
C	-1.545022000	0.704644000	0.010295000
C	-3.384813000	-2.426912000	-0.052701000
C	-3.427098000	-3.185891000	1.125902000
C	-3.887972000	-4.501531000	1.028816000

H	-3.934817000	-5.116830000	1.930423000
C	-4.280883000	-5.032508000	-0.196581000
H	-4.634650000	-6.064220000	-0.252691000
C	-4.220981000	-4.257316000	-1.351363000
H	-4.523148000	-4.681833000	-2.311553000
C	-3.768509000	-2.936820000	-1.301061000
C	-3.384794000	2.426911000	0.052726000
C	-3.768603000	2.936824000	1.301051000
C	-4.221043000	4.257332000	1.351313000
H	-4.523296000	4.681854000	2.311474000
C	-4.280804000	5.032532000	0.196528000
H	-4.634545000	6.064255000	0.252607000
C	-3.887781000	4.501551000	-1.028831000
H	-3.934515000	5.116856000	-1.930439000
C	-3.426937000	3.185897000	-1.125876000
C	2.975371000	2.616760000	-2.443063000
H	1.874457000	2.618550000	-2.498688000
H	3.365470000	3.208946000	-3.281592000
H	3.289362000	1.570419000	-2.573990000
C	3.694816000	2.079414000	2.535042000
H	3.813469000	2.687007000	3.441622000
H	2.729169000	1.554003000	2.591775000
H	4.491145000	1.317246000	2.533804000
C	-2.975312000	2.616765000	-2.443041000
H	-3.289299000	1.570423000	-2.573974000
H	-3.365393000	3.208949000	-3.281579000
H	-1.874396000	2.618556000	-2.498640000
C	-3.694853000	2.079421000	2.535050000
H	-3.813518000	2.687017000	3.441627000
H	-4.491184000	1.317255000	2.533802000
H	-2.729208000	1.554007000	2.591799000
C	3.694694000	-2.079425000	-2.535027000
H	3.813328000	-2.687016000	-3.441611000
H	2.729027000	-1.554044000	-2.591721000
H	4.490999000	-1.317232000	-2.533823000
C	2.975511000	-2.616757000	2.443111000
H	3.289694000	-1.570481000	2.574108000
H	1.874595000	-2.618348000	2.498698000
H	3.365474000	-3.209060000	3.281620000
C	-3.694613000	-2.079420000	-2.535054000
H	-3.813217000	-2.687012000	-3.441641000
H	-4.490914000	-1.317223000	-2.533880000
H	-2.728942000	-1.554044000	-2.591714000
C	-2.975609000	-2.616753000	2.443110000
H	-1.874694000	-2.618348000	2.498739000
H	-3.289792000	-1.570475000	2.574096000
H	-3.365607000	-3.209053000	3.281605000
C	5.175893000	-0.000001000	-0.000062000
C	5.885070000	0.750247000	-0.947784000
C	5.885165000	-0.750247000	0.947591000
C	7.277061000	0.749031000	-0.945749000
C	7.277156000	-0.749021000	0.945423000
C	7.975999000	0.000008000	-0.000195000
H	5.348237000	1.332074000	-1.696861000
H	5.348408000	-1.332081000	1.696716000
H	7.817328000	1.336190000	-1.690676000
H	7.817497000	-1.336178000	1.690298000
H	9.067964000	0.000011000	-0.000247000

C	-5.175891000	0.000006000	-0.000082000
C	-5.885175000	-0.750228000	0.947571000
C	-5.885055000	0.750245000	-0.947821000
C	-7.277167000	-0.748999000	0.945387000
C	-7.277046000	0.749031000	-0.945802000
C	-7.975997000	0.000020000	-0.000248000
H	-5.348428000	-1.332054000	1.696709000
H	-5.348212000	1.332062000	-1.696898000
H	-7.817519000	-1.336147000	1.690262000
H	-7.817304000	1.336183000	-1.690742000
H	-9.067962000	0.000026000	-0.000313000

1-Sn$_{OS}$ (wB97XD)
***E* =** -2577.752334
***H* =** -2577.698683
***G* =** -2577.842849
***G$_{qh}$* =** -2577.827379
***N$_{imag}$* =** 0

Sn	0.000015000	2.250569000	0.162219000
N	2.930325000	1.075636000	-0.007540000
N	2.930314000	-1.075674000	0.007602000
C	3.731866000	-0.000024000	0.000020000
C	1.577370000	0.697876000	0.007315000
C	1.577363000	-0.697899000	-0.007204000
C	3.393441000	2.430091000	0.046088000
C	3.413500000	3.184620000	-1.135726000
C	3.859550000	4.505850000	-1.048631000
H	3.888230000	5.118765000	-1.952556000
C	4.260183000	5.045189000	0.170817000
H	4.602267000	6.081179000	0.219732000
C	4.222015000	4.273569000	1.328877000
H	4.529348000	4.705227000	2.284193000
C	3.784314000	2.947592000	1.288546000
C	3.393407000	-2.430135000	-0.046045000
C	3.784379000	-2.947593000	-1.288490000
C	4.222058000	-4.273578000	-1.328835000
H	4.529475000	-4.705200000	-2.284140000
C	4.260101000	-5.045246000	-0.170804000
H	4.602166000	-6.081242000	-0.219731000
C	3.859357000	-4.505953000	1.048628000
H	3.887924000	-5.118911000	1.952527000
C	3.413327000	-3.184717000	1.135736000
Sn	-0.000012000	-2.250585000	-0.161978000
N	-2.930331000	-1.075631000	0.007547000
N	-2.930305000	1.075680000	-0.007608000
C	-3.731864000	0.000033000	-0.000081000
C	-1.577372000	-0.697880000	-0.007211000
C	-1.577358000	0.697896000	0.007306000
C	-3.393454000	-2.430082000	-0.046091000
C	-3.413627000	-3.184579000	1.135741000
C	-3.859679000	-4.505808000	1.048641000
H	-3.888445000	-5.118699000	1.952579000
C	-4.260205000	-5.045176000	-0.170829000
H	-4.602292000	-6.081165000	-0.219748000
C	-4.221927000	-4.273588000	-1.328906000
H	-4.529178000	-4.705268000	-2.284239000
C	-3.784221000	-2.947612000	-1.288571000

C	-3.393392000	2.430144000	0.046010000
C	-3.784448000	2.947595000	1.288431000
C	-4.222123000	4.273582000	1.328756000
H	-4.529604000	4.705199000	2.284043000
C	-4.260083000	5.045259000	0.170727000
H	-4.602147000	6.081256000	0.219638000
C	-3.859259000	4.505972000	-1.048681000
H	-3.887764000	5.118936000	-1.952578000
C	-3.413229000	3.184735000	-1.135768000
C	2.953052000	2.600431000	-2.443063000
H	3.286958000	3.216570000	-3.288391000
H	3.318588000	1.572768000	-2.590113000
H	1.851810000	2.546518000	-2.466014000
C	3.729704000	2.094644000	2.526406000
H	3.871351000	2.704021000	3.428418000
H	2.761483000	1.576782000	2.604974000
H	4.518861000	1.325323000	2.510884000
C	-2.952538000	2.600612000	-2.443049000
H	-3.318056000	1.572960000	-2.590225000
H	-3.286276000	3.216800000	-3.288407000
H	-1.851293000	2.546688000	-2.465792000
C	-3.730072000	2.094580000	2.526254000
H	-3.871859000	2.703911000	3.428275000
H	-4.519245000	1.325278000	2.510553000
H	-2.761877000	1.576689000	2.604960000
C	3.729912000	-2.094589000	-2.526317000
H	3.871644000	-2.703926000	-3.428342000
H	2.761709000	-1.576706000	-2.604963000
H	4.519081000	-1.325281000	-2.510677000
C	2.952710000	-2.600588000	2.443040000
H	3.318232000	-1.572934000	2.590188000
H	1.851466000	-2.546669000	2.465846000
H	3.286498000	-3.216770000	3.288382000
C	-3.729490000	-2.094700000	-2.526451000
H	-3.871064000	-2.704101000	-3.428458000
H	-4.518641000	-1.325370000	-2.511021000
H	-2.761257000	-1.576851000	-2.604948000
C	-2.953291000	-2.600357000	2.443102000
H	-1.852052000	-2.546441000	2.466146000
H	-3.318843000	-1.572691000	2.590094000
H	-3.287269000	-3.216474000	3.288416000
C	5.204511000	-0.000029000	-0.000020000
C	5.916049000	0.787517000	-0.916535000
C	5.916100000	-0.787580000	0.916452000
C	7.307950000	0.787560000	-0.913250000
C	7.308001000	-0.787630000	0.913083000
C	8.007634000	-0.000036000	-0.000104000
H	5.380478000	1.399066000	-1.642370000
H	5.380570000	-1.399126000	1.642318000
H	7.847783000	1.405333000	-1.633406000
H	7.847874000	-1.405406000	1.633205000
H	9.099591000	-0.000039000	-0.000137000
C	-5.204509000	0.000036000	-0.000128000
C	-5.916093000	-0.787517000	0.916345000
C	-5.916052000	0.787573000	-0.916648000
C	-7.307993000	-0.787573000	0.912980000
C	-7.307953000	0.787609000	-0.913359000
C	-8.007632000	0.000014000	-0.000208000

H	-5.380556000	-1.399063000	1.642208000
H	-5.380488000	1.399116000	-1.642491000
H	-7.847862000	-1.405351000	1.633105000
H	-7.847791000	1.405374000	-1.633518000
H	-9.099589000	0.000006000	-0.000240000

1-SiGe$_{CS}$

E **=** -4516.002298
H **=** -4515.950053
G **=** -4516.088071
***N*~imag~** **=** 0
SP **=** -4512.772750
***G*~SP~** **=** -4512.014490

Si	0.000000000	2.033463000	0.115823000
N	2.742660000	1.164493000	0.006187000
N	2.777683000	-1.006907000	0.011340000
C	3.554540000	0.092453000	0.009640000
C	1.372859000	0.765326000	0.023901000
C	1.401189000	-0.657270000	-0.010795000
C	3.201182000	2.524874000	0.081031000
C	3.228558000	3.301881000	-1.092011000
C	3.695684000	4.618861000	-0.985608000
H	3.730846000	5.244636000	-1.881144000
C	4.109428000	5.135864000	0.243694000
H	4.468799000	6.166164000	0.306768000
C	4.060713000	4.345464000	1.393393000
H	4.376141000	4.757924000	2.355218000
C	3.601591000	3.023038000	1.333902000
C	3.277977000	-2.351840000	-0.069444000
C	3.689726000	-2.831744000	-1.325799000
C	4.180710000	-4.142329000	-1.394075000
H	4.506049000	-4.540628000	-2.358547000
C	4.248471000	-4.938869000	-0.249597000
H	4.632430000	-5.959805000	-0.319464000
C	3.822837000	-4.440125000	0.983165000
H	3.873936000	-5.070618000	1.874590000
C	3.323925000	-3.135440000	1.098490000
Ge	0.000000000	-2.027229000	-0.115850000
N	-2.777684000	-1.006907000	0.011340000
N	-2.742660000	1.164493000	0.006187000
C	-3.554540000	0.092453000	0.009641000
C	-1.401189000	-0.657270000	-0.010795000
C	-1.372859000	0.765326000	0.023901000
C	-3.277977000	-2.351840000	-0.069444000
C	-3.323925000	-3.135441000	1.098490000
C	-3.822837000	-4.440125000	0.983164000
H	-3.873935000	-5.070619000	1.874589000
C	-4.248471000	-4.938869000	-0.249598000
H	-4.632430000	-5.959805000	-0.319464000
C	-4.180710000	-4.142329000	-1.394076000
H	-4.506049000	-4.540627000	-2.358547000
C	-3.689727000	-2.831743000	-1.325800000
C	-3.201182000	2.524874000	0.081031000
C	-3.601591000	3.023038000	1.333902000
C	-4.060713000	4.345464000	1.393392000
H	-4.376140000	4.757925000	2.355217000
C	-4.109428000	5.135865000	0.243693000
H	-4.468798000	6.166164000	0.306766000
C	-3.695683000	4.618860000	-0.985609000
H	-3.730846000	5.244636000	-1.881145000

C	-3.228558000	3.301880000	-1.092011000
C	2.753545000	2.743998000	-2.407928000
H	3.055630000	3.387968000	-3.246005000
H	3.136473000	1.727329000	-2.590595000
H	1.652984000	2.662385000	-2.405151000
C	3.533578000	2.151640000	2.561627000
H	3.693535000	2.741428000	3.475074000
H	2.553681000	1.653607000	2.634244000
H	4.302611000	1.361230000	2.531466000
C	-2.753545000	2.743997000	-2.407928000
H	-3.136474000	1.727328000	-2.590595000
H	-3.055630000	3.387967000	-3.246006000
H	-1.652985000	2.662384000	-2.405151000
C	-3.533577000	2.151641000	2.561627000
H	-3.693535000	2.741429000	3.475074000
H	-4.302610000	1.361230000	2.531466000
H	-2.553680000	1.653608000	2.634244000
C	3.603008000	-1.953145000	-2.547415000
H	3.763236000	-2.535193000	-3.465744000
H	2.617655000	-1.464770000	-2.610465000
H	4.364029000	-1.154957000	-2.516937000
C	2.839498000	-2.598034000	2.420051000
H	3.187734000	-1.569037000	2.602187000
H	1.737174000	-2.555154000	2.430103000
H	3.172184000	-3.233015000	3.253393000
C	-3.603009000	-1.953145000	-2.547415000
H	-3.763236000	-2.535192000	-3.465744000
H	-4.364031000	-1.154957000	-2.516937000
H	-2.617657000	-1.464768000	-2.610465000
C	-2.839497000	-2.598035000	2.420051000
H	-1.737174000	-2.555155000	2.430103000
H	-3.187733000	-1.569038000	2.602187000
H	-3.172184000	-3.233016000	3.253393000
C	5.027787000	0.114837000	0.010634000
C	5.731892000	0.921346000	-0.902754000
C	5.753661000	-0.671591000	0.924476000
C	7.127339000	0.939036000	-0.900130000
C	7.149084000	-0.650506000	0.922602000
C	7.840383000	0.153995000	0.011363000
H	5.188014000	1.529906000	-1.623324000
H	5.226631000	-1.294418000	1.645433000
H	7.658901000	1.568537000	-1.617551000
H	7.697616000	-1.264734000	1.640484000
H	8.933032000	0.169214000	0.011630000
C	-5.027787000	0.114837000	0.010635000
C	-5.753661000	-0.671592000	0.924476000
C	-5.731893000	0.921347000	-0.902752000
C	-7.149084000	-0.650507000	0.922603000
C	-7.127340000	0.939037000	-0.900129000
C	-7.840383000	0.153995000	0.011365000
H	-5.226631000	-1.294419000	1.645433000
H	-5.188015000	1.529907000	-1.623322000
H	-7.697616000	-1.264735000	1.640485000
H	-7.658902000	1.568538000	-1.617549000
H	-8.933032000	0.169214000	0.011632000

1-SiSn$_{OS}$

***E* =** -2653.582728
***H* =** -2653.529802
***G* =** -2653.671415

N_{imag} **=** 0
SP **=** -2650.827272
G_{SP} **=** -2650.072168

Si	0.000004000	2.033449000	0.115660000
N	2.742661000	1.164492000	0.006260000
N	2.777729000	-1.006899000	0.011270000
C	3.554552000	0.092469000	0.009665000
C	1.372844000	0.765304000	0.023868000
C	1.401204000	-0.657294000	-0.010937000
C	3.201096000	2.524916000	0.080934000
C	3.228384000	3.301739000	-1.092216000
C	3.695325000	4.618801000	-0.986007000
H	3.730408000	5.244451000	-1.881634000
C	4.108997000	5.136042000	0.243223000
H	4.468240000	6.166396000	0.306141000
C	4.060380000	4.345799000	1.393038000
H	4.375767000	4.758429000	2.354805000
C	3.601430000	3.023304000	1.333730000
C	3.277977000	-2.351857000	-0.069383000
C	3.689896000	-2.831769000	-1.325667000
C	4.180771000	-4.142393000	-1.393906000
H	4.506247000	-4.540688000	-2.358333000
C	4.248279000	-4.938969000	-0.249433000
H	4.632157000	-5.959939000	-0.319261000
C	3.822535000	-4.440193000	0.983276000
H	3.873471000	-5.070698000	1.874704000
C	3.323729000	-3.135458000	1.098557000
Ge	0.000013000	-2.027216000	-0.116177000
N	-2.777708000	-1.006908000	0.011170000
N	-2.742654000	1.164485000	0.006281000
C	-3.554536000	0.092456000	0.009624000
C	-1.401185000	-0.657296000	-0.010980000
C	-1.372832000	0.765304000	0.023821000
C	-3.277955000	-2.351864000	-0.069546000
C	-3.323779000	-3.135486000	1.098376000
C	-3.822578000	-4.440219000	0.983041000
H	-3.873571000	-5.070739000	1.874455000
C	-4.248244000	-4.938973000	-0.249705000
H	-4.632116000	-5.959942000	-0.319574000
C	-4.180661000	-4.142379000	-1.394159000
H	-4.506072000	-4.540658000	-2.358615000
C	-3.689792000	-2.831755000	-1.325867000
C	-3.201112000	2.524894000	0.081084000
C	-3.601350000	3.023179000	1.333952000
C	-4.060353000	4.345650000	1.393397000
H	-4.375663000	4.758199000	2.355223000
C	-4.109121000	5.135969000	0.243641000
H	-4.468405000	6.166302000	0.306665000
C	-3.695553000	4.618828000	-0.985666000
H	-3.730760000	5.244537000	-1.881247000
C	-3.228558000	3.301797000	-1.092012000
C	2.753409000	2.743589000	-2.408035000
H	3.055339000	3.387497000	-3.246219000
H	3.136510000	1.726964000	-2.590580000
H	1.652870000	2.661782000	-2.405168000
C	3.533462000	2.152087000	2.561589000
H	3.693938000	2.741933000	3.474910000
H	2.553367000	1.654483000	2.634554000
H	4.302147000	1.361350000	2.531293000
C	-2.753710000	2.743764000	-2.407926000
H	-3.136755000	1.727121000	-2.590485000

H	-3.055798000	3.387702000	-3.246029000
H	-1.653164000	2.662049000	-2.405205000
C	-3.533223000	2.151881000	2.561744000
H	-3.693633000	2.741657000	3.475122000
H	-4.301876000	1.361111000	2.531470000
H	-2.553099000	1.654315000	2.634578000
C	3.603480000	-1.953100000	-2.547257000
H	3.764119000	-2.535062000	-3.465571000
H	2.618069000	-1.464879000	-2.610611000
H	4.364357000	-1.154788000	-2.516441000
C	2.839184000	-2.598096000	2.420095000
H	3.186741000	-1.568831000	2.601958000
H	1.736836000	-2.555975000	2.430314000
H	3.172446000	-3.232696000	3.253503000
C	-3.603284000	-1.953074000	-2.547442000
H	-3.763855000	-2.535028000	-3.465773000
H	-4.364162000	-1.154762000	-2.516677000
H	-2.617867000	-1.464855000	-2.610718000
C	-2.839316000	-2.598144000	2.419951000
H	-1.736968000	-2.556017000	2.430234000
H	-3.186890000	-1.568883000	2.601809000
H	-3.172624000	-3.232757000	3.253329000
C	5.027793000	0.114849000	0.010783000
C	5.732012000	0.921397000	-0.902480000
C	5.753563000	-0.671638000	0.924659000
C	7.127460000	0.939076000	-0.899694000
C	7.148987000	-0.650562000	0.922947000
C	7.840402000	0.153983000	0.011836000
H	5.188258000	1.530014000	-1.623089000
H	5.226475000	-1.294508000	1.645534000
H	7.659106000	1.568617000	-1.617019000
H	7.697431000	-1.264838000	1.640857000
H	8.933053000	0.169197000	0.012230000
C	-5.027775000	0.114825000	0.010766000
C	-5.753522000	-0.671752000	0.924581000
C	-5.732012000	0.921450000	-0.902415000
C	-7.148946000	-0.650691000	0.922889000
C	-7.127460000	0.939115000	-0.899607000
C	-7.840382000	0.153931000	0.011860000
H	-5.226415000	-1.294686000	1.645388000
H	-5.188270000	1.530141000	-1.622973000
H	-7.697374000	-1.265039000	1.640749000
H	-7.659123000	1.568717000	-1.616867000
H	-8.933032000	0.169133000	0.012270000

1-GeSn$_{OS}$

E **=** -4440.967193
H **=** -4440.913659
G **=** -4441.056471
N_{imag} **=** 0
SP **=** -4437.394733
G_{SP} **=** -4436.641684

Ge	0.000007000	2.044960000	0.149730000
N	2.809194000	1.156819000	0.005084000
N	2.913834000	-1.007749000	0.024332000
C	3.663067000	0.114083000	0.013961000
C	1.459483000	0.708306000	0.026758000
C	1.532952000	-0.702431000	0.002474000
C	3.219086000	2.533249000	0.067789000
C	3.214392000	3.301349000	-1.111450000

C	3.629617000	4.636418000	-1.016350000
H	3.638894000	5.256007000	-1.916811000
C	4.024918000	5.178829000	0.208103000
H	4.343294000	6.223000000	0.262543000
C	4.010656000	4.395771000	1.363722000
H	4.312637000	4.827449000	2.321406000
C	3.604234000	3.055685000	1.315481000
C	3.449452000	-2.339624000	-0.046541000
C	3.869160000	-2.821166000	-1.300114000
C	4.380820000	-4.124359000	-1.359414000
H	4.711532000	-4.524154000	-2.321393000
C	4.463701000	-4.911355000	-0.209342000
H	4.863204000	-5.926698000	-0.272830000
C	4.036054000	-4.409377000	1.021334000
H	4.102910000	-5.031022000	1.917872000
C	3.516641000	-3.111880000	1.128425000
Sn	-0.000010000	-2.290159000	-0.150020000
N	-2.913843000	-1.007719000	0.024332000
N	-2.809185000	1.156847000	0.004950000
C	-3.663069000	0.114117000	0.013903000
C	-1.532961000	-0.702413000	0.002401000
C	-1.459480000	0.708322000	0.026709000
C	-3.449474000	-2.339594000	-0.046432000
C	-3.516601000	-3.111785000	1.128580000
C	-4.036028000	-4.409284000	1.021589000
H	-4.102836000	-5.030880000	1.918164000
C	-4.463751000	-4.911326000	-0.209035000
H	-4.863266000	-5.926669000	-0.272445000
C	-4.380933000	-4.124391000	-1.359153000
H	-4.711707000	-4.524234000	-2.321092000
C	-3.869259000	-2.821200000	-1.299954000
C	-3.219055000	2.533287000	0.067593000
C	-3.604287000	3.055744000	1.315248000
C	-4.010688000	4.395837000	1.363445000
H	-4.312738000	4.827530000	2.321101000
C	-4.024845000	5.178884000	0.207817000
H	-4.343207000	6.223061000	0.262222000
C	-3.629458000	4.636454000	-1.016599000
H	-3.638653000	5.256035000	-1.917067000
C	-3.214250000	3.301377000	-1.111655000
C	2.760322000	2.714671000	-2.422507000
H	3.044075000	3.359151000	-3.266567000
H	3.175653000	1.708834000	-2.593242000
H	1.662715000	2.599068000	-2.422939000
C	3.576775000	2.190728000	2.549294000
H	3.715439000	2.792828000	3.458154000
H	2.619054000	1.652419000	2.629407000
H	4.377930000	1.432818000	2.520498000
C	-2.760088000	2.714683000	-2.422675000
H	-3.175415000	1.708849000	-2.593436000
H	-3.043774000	3.359160000	-3.266760000
H	-1.662482000	2.599070000	-2.423029000
C	-3.576951000	2.190789000	2.549066000
H	-3.715686000	2.792893000	3.457913000
H	-4.378117000	1.432892000	2.520198000
H	-2.619247000	1.652464000	2.629263000
C	3.774893000	-1.951048000	-2.527261000
H	3.942524000	-2.537022000	-3.441725000
H	2.785684000	-1.471614000	-2.596014000
H	4.528426000	-1.145687000	-2.498922000
C	3.038171000	-2.569657000	2.450831000

H	3.357968000	-1.528193000	2.611713000
H	1.935485000	-2.560823000	2.484735000
H	3.405613000	-3.182213000	3.286371000
C	-3.775069000	-1.951141000	-2.527148000
H	-3.942768000	-2.537156000	-3.441573000
H	-4.528594000	-1.145772000	-2.498795000
H	-2.785861000	-1.471718000	-2.595992000
C	-3.038047000	-2.569494000	2.450928000
H	-1.935360000	-2.560647000	2.484759000
H	-3.357844000	-1.528025000	2.611781000
H	-3.405428000	-3.182013000	3.286522000
C	5.133156000	0.183216000	0.008734000
C	5.808988000	1.023083000	-0.897450000
C	5.890347000	-0.592277000	0.907794000
C	7.203037000	1.084263000	-0.901402000
C	7.284330000	-0.528966000	0.897796000
C	7.945983000	0.308989000	-0.005382000
H	5.243774000	1.623457000	-1.608262000
H	5.388286000	-1.239695000	1.624776000
H	7.710478000	1.739666000	-1.613169000
H	7.855340000	-1.135900000	1.604357000
H	9.037616000	0.357787000	-0.010896000
C	-5.133161000	0.183246000	0.008718000
C	-5.890311000	-0.592166000	0.907884000
C	-5.809040000	1.023000000	-0.897535000
C	-7.284295000	-0.528873000	0.897933000
C	-7.203090000	1.084161000	-0.901443000
C	-7.945994000	0.308977000	-0.005310000
H	-5.388217000	-1.239503000	1.624915000
H	-5.243867000	1.623297000	-1.608441000
H	-7.855271000	-1.135740000	1.604579000
H	-7.710566000	1.739477000	-1.613265000
H	-9.037627000	0.357759000	-0.010788000

TS-Si$_{ace}$

E **=** -2805.867051
H **=** -2805.812098
G **=** -2805.955596
N_{imag} **=** 1, 298.2 cm^{-1}
SP **=** -2803.392141
G_{SP} **=** -2802.607696

Si	0.000004000	-1.962962000	-0.324874000
N	-2.746661000	-1.093328000	0.018887000
N	-2.733741000	1.077345000	0.063069000
C	-3.536961000	-0.004229000	0.096476000
C	-1.382333000	-0.718457000	-0.057600000
C	-1.377009000	0.692048000	-0.048275000
C	-3.222418000	-2.444212000	0.149892000
C	-3.388914000	-3.233036000	-1.001760000
C	-3.872432000	-4.538291000	-0.828519000
H	-4.016496000	-5.172225000	-1.707411000
C	-4.165781000	-5.032201000	0.442768000
H	-4.540384000	-6.052645000	0.556894000
C	-3.978653000	-4.230165000	1.570977000
H	-4.201433000	-4.624045000	2.565929000
C	-3.501002000	-2.920010000	1.445182000
C	-3.205370000	2.433711000	-0.000153000
C	-3.707076000	2.902344000	-1.228026000
C	-4.179361000	4.220858000	-1.276160000
H	-4.573866000	4.611788000	-2.217602000

C	-4.140159000	5.035306000	-0.142971000
H	-4.511510000	6.061820000	-0.197888000
C	-3.620656000	4.548214000	1.058212000
H	-3.583890000	5.193875000	1.939478000
C	-3.137016000	3.236475000	1.152931000
Si	-0.000009000	1.929494000	-0.379938000
N	2.733734000	1.077357000	0.063022000
N	2.746652000	-1.093318000	0.018922000
C	3.536953000	-0.004215000	0.096458000
C	1.377001000	0.692054000	-0.048294000
C	1.382330000	-0.718450000	-0.057586000
C	3.205354000	2.433723000	-0.000267000
C	3.137030000	3.236531000	1.152786000
C	3.620634000	4.548279000	1.057992000
H	3.583890000	5.193975000	1.939233000
C	4.140074000	5.035334000	-0.143233000
H	4.511397000	6.061855000	-0.198208000
C	4.179252000	4.220839000	-1.276388000
H	4.573709000	4.611738000	-2.217863000
C	3.707002000	2.902315000	-1.228180000
C	3.222409000	-2.444184000	0.150086000
C	3.501052000	-2.919786000	1.445437000
C	3.978789000	-4.229893000	1.571408000
H	4.201614000	-4.623619000	2.566411000
C	4.165951000	-5.032072000	0.443309000
H	4.540632000	-6.052472000	0.557570000
C	3.872525000	-4.538366000	-0.828040000
H	4.016601000	-5.172423000	-1.706840000
C	3.388909000	-3.233170000	-1.001461000
C	-3.036309000	-2.717717000	-2.372909000
H	-3.624845000	-3.225678000	-3.150960000
H	-3.183332000	-1.632088000	-2.466344000
H	-1.967966000	-2.900393000	-2.575170000
C	-3.287563000	-2.035868000	2.646792000
H	-3.381095000	-2.607327000	3.580839000
H	-2.289113000	-1.570874000	2.618688000
H	-4.027862000	-1.218531000	2.675402000
C	3.036173000	-2.718112000	-2.372677000
H	3.183379000	-1.632530000	-2.466391000
H	3.624486000	-3.226367000	-3.150703000
H	1.967757000	-2.900634000	-2.574718000
C	3.287603000	-2.035491000	2.646933000
H	3.381001000	-2.606860000	3.581050000
H	4.027992000	-1.218234000	2.675518000
H	2.289207000	-1.570391000	2.618703000
C	-3.724769000	2.010107000	-2.443203000
H	-3.931950000	2.589268000	-3.354079000
H	-2.760905000	1.491641000	-2.569755000
H	-4.503280000	1.233452000	-2.355221000
C	-2.538645000	2.712942000	2.431876000
H	-2.874985000	1.689131000	2.659375000
H	-1.440148000	2.662509000	2.337887000
H	-2.787116000	3.362183000	3.283471000
C	3.724664000	2.010026000	-2.443319000
H	3.931825000	2.589146000	-3.354225000
H	4.503173000	1.233372000	-2.355321000
H	2.760795000	1.491557000	-2.569826000
C	2.538721000	2.713039000	2.431777000
H	1.440216000	2.662644000	2.337860000
H	2.875040000	1.689221000	2.659270000
H	2.787269000	3.362286000	3.283346000

C	-5.003346000	0.004163000	0.217924000
C	-5.797152000	-0.777643000	-0.642067000
C	-5.634503000	0.797651000	1.194124000
C	-7.187663000	-0.766583000	-0.524361000
C	-7.025506000	0.806699000	1.306069000
C	-7.806269000	0.024749000	0.448830000
H	-5.325639000	-1.386425000	-1.412176000
H	-5.035073000	1.402693000	1.873136000
H	-7.790284000	-1.376951000	-1.201033000
H	-7.500380000	1.426146000	2.070517000
H	-8.895316000	0.032638000	0.538503000
C	5.003343000	0.004181000	0.217845000
C	5.634554000	0.797694000	1.193987000
C	5.797096000	-0.777652000	-0.642169000
C	7.025564000	0.806740000	1.305854000
C	7.187614000	-0.766595000	-0.524543000
C	7.806277000	0.024763000	0.448593000
H	5.035164000	1.402756000	1.873017000
H	5.325531000	-1.386452000	-1.412233000
H	7.500484000	1.426206000	2.070257000
H	7.790195000	-1.376984000	-1.201231000
H	8.895328000	0.032650000	0.538204000
C	0.000011000	0.664827000	-2.886842000
C	0.000034000	-0.574943000	-2.909108000
H	0.000041000	-1.566561000	-3.325991000
H	0.000009000	1.652704000	-3.317769000

TS-Ge$_{ace}$

***E* =** -6380.635597
***H* =** -6380.579364
***G* =** -6380.727063
N_{imag} = 1, 250.2 cm^{-1}
***SP* =** -6376.529600
G_{SP} = -6375.749680

Ge	-0.000006000	-2.036463000	0.241427000
N	2.799478000	-1.086351000	-0.075343000
N	2.789317000	1.081934000	-0.115085000
C	3.594708000	0.000665000	-0.131354000
C	1.436399000	-0.709969000	-0.028388000
C	1.431633000	0.696102000	-0.032599000
C	3.270933000	-2.439530000	-0.190921000
C	3.409570000	-3.226662000	0.964568000
C	3.879760000	-4.539060000	0.802050000
H	4.002142000	-5.171484000	1.685507000
C	4.186803000	-5.040347000	-0.462023000
H	4.550076000	-6.065805000	-0.567654000
C	4.028205000	-4.238659000	-1.595814000
H	4.262713000	-4.638000000	-2.585876000
C	3.564430000	-2.923337000	-1.481088000
C	3.256912000	2.438906000	-0.045263000
C	3.721964000	2.916664000	1.193349000
C	4.182016000	4.239435000	1.248141000
H	4.548444000	4.637455000	2.197924000
C	4.165873000	5.048941000	0.110914000
H	4.526839000	6.078845000	0.171326000
C	3.682343000	4.552706000	-1.101650000
H	3.663214000	5.194525000	-1.986253000
C	3.212165000	3.236636000	-1.203128000
Ge	-0.000013000	2.011470000	0.317563000
N	-2.789338000	1.081922000	-0.115114000

N	-2.799485000	-1.086361000	-0.075360000
C	-3.594722000	0.000647000	-0.131390000
C	-1.431653000	0.696098000	-0.032606000
C	-1.436412000	-0.709971000	-0.028389000
C	-3.256947000	2.438889000	-0.045286000
C	-3.212207000	3.236630000	-1.203143000
C	-3.682408000	4.552691000	-1.101654000
H	-3.663286000	5.194519000	-1.986251000
C	-4.165951000	5.048906000	0.110913000
H	-4.526935000	6.078804000	0.171333000
C	-4.182081000	4.239391000	1.248135000
H	-4.548516000	4.637397000	2.197920000
C	-3.722006000	2.916628000	1.193331000
C	-3.270915000	-2.439554000	-0.190854000
C	-3.564355000	-2.923466000	-1.480993000
C	-4.028063000	-4.238819000	-1.595637000
H	-4.262526000	-4.638243000	-2.585675000
C	-4.186645000	-5.040435000	-0.461792000
H	-4.549859000	-6.065920000	-0.567358000
C	-3.879666000	-4.539040000	0.802255000
H	-4.002045000	-5.171405000	1.685754000
C	-3.409553000	-3.226603000	0.964691000
C	3.056714000	-2.711125000	2.337053000
H	3.798790000	-3.038713000	3.081783000
H	2.975887000	-1.616616000	2.370989000
H	2.075770000	-3.107260000	2.646643000
C	3.382826000	-2.038638000	-2.687514000
H	3.489095000	-2.611887000	-3.619084000
H	2.388855000	-1.563485000	-2.679150000
H	4.131527000	-1.228527000	-2.701629000
C	-3.056791000	-2.710933000	2.337152000
H	-2.975965000	-1.616420000	2.370984000
H	-3.798925000	-3.038447000	3.081857000
H	-2.075870000	-3.107036000	2.646860000
C	-3.382747000	-2.038843000	-2.687475000
H	-3.489068000	-2.612138000	-3.619010000
H	-4.131407000	-1.228695000	-2.701613000
H	-2.388753000	-1.563738000	-2.679161000
C	3.715720000	2.028978000	2.411939000
H	3.898239000	2.612380000	3.325376000
H	2.752055000	1.505518000	2.517621000
H	4.500140000	1.256362000	2.344489000
C	2.652920000	2.700134000	-2.494653000
H	3.031929000	1.691340000	-2.722995000
H	1.555974000	2.606688000	-2.419813000
H	2.890702000	3.363589000	-3.338202000
C	-3.715736000	2.028932000	2.411914000
H	-3.898296000	2.612316000	3.325353000
H	-4.500114000	1.256275000	2.344448000
H	-2.752046000	1.505520000	2.517602000
C	-2.652943000	2.700149000	-2.494668000
H	-1.555996000	2.606722000	-2.419820000
H	-3.031932000	1.691351000	-2.723023000
H	-2.890729000	3.363608000	-3.338213000
C	5.063504000	0.007174000	-0.207636000
C	5.827469000	-0.772281000	0.681291000
C	5.727403000	0.797503000	-1.164398000
C	7.221089000	-0.763967000	0.609028000
C	7.121426000	0.804353000	-1.230384000
C	7.872419000	0.023322000	-0.346058000
H	5.328643000	-1.374919000	1.439188000

H	5.151040000	1.402229000	-1.863571000
H	7.800486000	-1.372445000	1.307383000
H	7.622311000	1.421304000	-1.980142000
H	8.963836000	0.029364000	-0.400025000
C	-5.063517000	0.007147000	-0.207702000
C	-5.727397000	0.797451000	-1.164498000
C	-5.827502000	-0.772290000	0.681225000
C	-7.121418000	0.804295000	-1.230518000
C	-7.221120000	-0.763982000	0.608928000
C	-7.872430000	0.023282000	-0.346192000
H	-5.151020000	1.402164000	-1.863670000
H	-5.328694000	-1.374908000	1.439149000
H	-7.622287000	1.421227000	-1.980302000
H	-7.800531000	-1.372445000	1.307283000
H	-8.963845000	0.029318000	-0.400186000
C	0.000019000	0.605499000	2.865127000
C	0.000064000	-0.633055000	2.855781000
H	0.000122000	-1.636650000	3.242420000
H	-0.000001000	1.592607000	3.295397000

TS-Sn$_{ace}$

E **=** -2655.800386
H **=** -2655.743326
G **=** -2655.894193
N_{imag} **=** 1, 249.2 cm^{-1}
SP **=** -2652.645095
G_{SP} **=** -2651.868399

Sn	0.000005000	-2.217041000	-0.113934000
N	-2.959643000	-1.079025000	0.127991000
N	-2.951561000	1.084570000	0.173416000
C	-3.759997000	0.005108000	0.162602000
C	-1.595237000	-0.702809000	0.121027000
C	-1.590623000	0.699953000	0.125832000
C	-3.431881000	-2.433955000	0.209854000
C	-3.537427000	-3.204992000	-0.960177000
C	-3.993611000	-4.526143000	-0.826804000
H	-4.087436000	-5.146762000	-1.722127000
C	-4.323657000	-5.049473000	0.421878000
H	-4.675083000	-6.081137000	0.504528000
C	-4.203666000	-4.261669000	1.570540000
H	-4.457961000	-4.677784000	2.548707000
C	-3.752187000	-2.940193000	1.485881000
C	-3.419656000	2.440125000	0.085917000
C	-3.825351000	2.920442000	-1.173134000
C	-4.276641000	4.245210000	-1.248710000
H	-4.596050000	4.645316000	-2.214444000
C	-4.312916000	5.053337000	-0.110908000
H	-4.666210000	6.084796000	-0.187658000
C	-3.893864000	4.553176000	1.123792000
H	-3.918664000	5.193156000	2.009548000
C	-3.433395000	3.235265000	1.246630000
Sn	-0.000007000	2.195663000	-0.242050000
N	2.951596000	1.084576000	0.173233000
N	2.959700000	-1.079022000	0.127879000
C	3.760042000	0.005122000	0.162487000
C	1.590663000	0.699946000	0.125614000
C	1.595290000	-0.702815000	0.120842000
C	3.419681000	2.440136000	0.085756000
C	3.433442000	3.235247000	1.246489000
C	3.893912000	4.553160000	1.123671000

H	3.918733000	5.193120000	2.009441000
C	4.312939000	5.053349000	-0.111027000
H	4.666236000	6.084809000	-0.187759000
C	4.276635000	4.245251000	-1.248849000
H	4.596022000	4.645380000	-2.214580000
C	3.825344000	2.920483000	-1.173293000
C	3.431962000	-2.433937000	0.209881000
C	3.752200000	-2.940063000	1.485967000
C	4.203704000	-4.261523000	1.570759000
H	4.457942000	-4.677556000	2.548976000
C	4.323805000	-5.049409000	0.422166000
H	4.675261000	-6.081055000	0.504919000
C	3.993845000	-4.526180000	-0.826583000
H	4.087774000	-5.146859000	-1.721854000
C	3.537623000	-3.205058000	-0.960084000
C	-3.184828000	-2.664647000	-2.324645000
H	-4.009815000	-2.836308000	-3.035418000
H	-2.947266000	-1.593411000	-2.309572000
H	-2.298748000	-3.184636000	-2.722813000
C	-3.613891000	-2.069344000	2.708022000
H	-3.743210000	-2.654833000	3.628965000
H	-2.624466000	-1.585699000	2.735295000
H	-4.369614000	-1.265556000	2.708386000
C	3.185016000	-2.664818000	-2.324587000
H	2.948521000	-1.593341000	-2.309742000
H	4.009539000	-2.837520000	-3.035634000
H	2.298230000	-3.184021000	-2.722213000
C	3.613816000	-2.069118000	2.708030000
H	3.743024000	-2.654544000	3.629029000
H	4.369573000	-1.265361000	2.708407000
H	2.624408000	-1.585432000	2.735174000
C	-3.772075000	2.031168000	-2.389667000
H	-3.910447000	2.614557000	-3.310806000
H	-2.809118000	1.499603000	-2.453601000
H	-4.565173000	1.265249000	-2.354398000
C	-2.946864000	2.694398000	2.565967000
H	-3.336248000	1.684297000	2.768988000
H	-1.847539000	2.602756000	2.556578000
H	-3.233725000	3.354531000	3.396745000
C	3.772007000	2.031236000	-2.389844000
H	3.910521000	2.614617000	-3.310967000
H	4.564978000	1.265189000	-2.354542000
H	2.808966000	1.499829000	-2.453834000
C	2.946929000	2.694355000	2.565823000
H	1.847601000	2.602737000	2.556461000
H	3.336293000	1.684239000	2.768811000
H	3.233825000	3.354461000	3.396611000
C	-5.230760000	0.011455000	0.184650000
C	-5.961687000	-0.755498000	-0.742243000
C	-5.930613000	0.789934000	1.125441000
C	-7.357058000	-0.748004000	-0.721889000
C	-7.326189000	0.796723000	1.139345000
C	-8.043867000	0.027051000	0.218201000
H	-5.435078000	-1.346647000	-1.490017000
H	-5.381228000	1.385796000	1.853198000
H	-7.909769000	-1.347081000	-1.449440000
H	-7.854620000	1.404700000	1.877469000
H	-9.136537000	0.032768000	0.231600000
C	5.230806000	0.011477000	0.184572000
C	5.930636000	0.789963000	1.125374000
C	5.961757000	-0.755482000	-0.742300000

C	7.326213000	0.796750000	1.139314000
C	7.357127000	-0.747988000	-0.721910000
C	8.043913000	0.027072000	0.218193000
H	5.381236000	1.385831000	1.853113000
H	5.435172000	-1.346638000	-1.490084000
H	7.854624000	1.404730000	1.877448000
H	7.909856000	-1.347069000	-1.449444000
H	9.136583000	0.032788000	0.231620000
C	-0.000633000	0.557865000	-2.926615000
C	-0.000721000	-0.677384000	-2.896806000
H	-0.000844000	-1.697829000	-3.232530000
H	-0.000701000	1.547491000	-3.347614000

TS-SiGe$_{ace}$

***E* =** -4593.250926
***H* =** -4593.195478
***G* =** -4593.340086
N_{imag} = 1, 275.6 cm^{-1}
***SP* =** -4589.960479
G_{SP} = -4589.177738

Si	0.000000000	1.996306000	0.313227000
N	-2.754791000	1.166112000	-0.030425000
N	-2.778951000	-1.002974000	-0.098508000
C	-3.565193000	0.092659000	-0.114390000
C	-1.395051000	0.764864000	0.035049000
C	-1.418210000	-0.643104000	0.008286000
C	-3.208214000	2.525603000	-0.152519000
C	-3.361255000	3.311128000	1.003157000
C	-3.825358000	4.624397000	0.837359000
H	-3.958404000	5.255843000	1.719797000
C	-4.113187000	5.129270000	-0.430792000
H	-4.472526000	6.155825000	-0.539110000
C	-3.940183000	4.330095000	-1.563325000
H	-4.158870000	4.732213000	-2.555896000
C	-3.482264000	3.012289000	-1.444918000
C	-3.269513000	-2.352711000	-0.041083000
C	-3.758087000	-2.826331000	1.190138000
C	-4.240742000	-4.141372000	1.234842000
H	-4.626046000	-4.536023000	2.178524000
C	-4.223199000	-4.947683000	0.095339000
H	-4.602099000	-5.971556000	0.147769000
C	-3.715514000	-4.456142000	-1.109180000
H	-3.695585000	-5.095629000	-1.995450000
C	-3.222372000	-3.147699000	-1.200660000
Ge	0.000000000	-1.973206000	0.343755000
N	2.778952000	-1.002973000	-0.098506000
N	2.754792000	1.166113000	-0.030423000
C	3.565194000	0.092659000	-0.114388000
C	1.418211000	-0.643103000	0.008289000
C	1.395052000	0.764865000	0.035051000
C	3.269513000	-2.352711000	-0.041079000
C	3.222372000	-3.147700000	-1.200655000
C	3.715514000	-4.456144000	-1.109174000
H	3.695584000	-5.095631000	-1.995444000
C	4.223198000	-4.947683000	0.095345000
H	4.602099000	-5.971556000	0.147777000
C	4.240742000	-4.141371000	1.234847000
H	4.626046000	-4.536021000	2.178530000
C	3.758088000	-2.826330000	1.190142000
C	3.208215000	2.525603000	-0.152518000

C	3.482266000	3.012288000	-1.444917000
C	3.940185000	4.330093000	-1.563325000
H	4.158872000	4.732211000	-2.555897000
C	4.113187000	5.129270000	-0.430793000
H	4.472526000	6.155825000	-0.539113000
C	3.825357000	4.624399000	0.837358000
H	3.958402000	5.255846000	1.719795000
C	3.361254000	3.311130000	1.003157000
C	-3.013939000	2.784936000	2.371447000
H	-3.604714000	3.287492000	3.151413000
H	-3.161543000	1.698774000	2.456178000
H	-1.946273000	2.966242000	2.578134000
C	-3.285295000	2.131119000	-2.651511000
H	-3.370611000	2.709082000	-3.582347000
H	-2.294857000	1.649173000	-2.627436000
H	-4.039337000	1.326523000	-2.683399000
C	3.013936000	2.784938000	2.371447000
H	3.161592000	1.698785000	2.456194000
H	3.604669000	3.287533000	3.151419000
H	1.946256000	2.966190000	2.578109000
C	3.285298000	2.131116000	-2.651510000
H	3.370611000	2.709079000	-3.582346000
H	4.039342000	1.326523000	-2.683397000
H	2.294862000	1.649167000	-2.627434000
C	-3.752009000	-1.942512000	2.411665000
H	-3.956296000	-2.525503000	3.320728000
H	-2.780731000	-1.436218000	2.531378000
H	-4.522107000	-1.156154000	2.337050000
C	-2.637731000	-2.617812000	-2.483696000
H	-2.986617000	-1.597493000	-2.708321000
H	-1.539428000	-2.555044000	-2.399171000
H	-2.886081000	-3.268959000	-3.333772000
C	3.752010000	-1.942509000	2.411669000
H	3.956297000	-2.525499000	3.320732000
H	4.522107000	-1.156151000	2.337052000
H	2.780732000	-1.436216000	2.531382000
C	2.637732000	-2.617815000	-2.483692000
H	1.539428000	-2.555047000	-2.399168000
H	2.986617000	-1.597496000	-2.708318000
H	2.886082000	-3.268962000	-3.333767000
C	-5.032368000	0.109135000	-0.222067000
C	-5.805764000	0.898051000	0.650210000
C	-5.685884000	-0.669103000	-1.195958000
C	-7.197347000	0.909144000	0.546340000
C	-7.077911000	-0.656245000	-1.293905000
C	-7.837980000	0.132922000	-0.424801000
H	-5.317497000	1.494967000	1.419099000
H	-5.103047000	-1.279455000	-1.884499000
H	-7.783608000	1.524949000	1.232410000
H	-7.569826000	-1.264084000	-2.056910000
H	-8.927858000	0.142384000	-0.503557000
C	5.032369000	0.109135000	-0.222067000
C	5.685884000	-0.669106000	-1.195956000
C	5.805766000	0.898054000	0.650206000
C	7.077910000	-0.656249000	-1.293906000
C	7.197349000	0.909146000	0.546334000
C	7.837980000	0.132921000	-0.424806000
H	5.103045000	-1.279460000	-1.884495000
H	5.317501000	1.494973000	1.419093000
H	7.569824000	-1.264090000	-2.056910000
H	7.783612000	1.524954000	1.232400000

H	8.927858000	0.142383000	-0.503563000
C	-0.000004000	-0.610211000	2.894782000
C	-0.000007000	0.628893000	2.893687000
H	-0.000012000	1.624116000	3.302188000
H	-0.000002000	-1.599300000	3.320940000

TS-SiSn$_{ace}$

E **=** -2730.832783
H **=** -2730.776664
G **=** -2730.925252
$\boldsymbol{N_{imag}}$ **=** 1, 269.7 cm^{-1}
SP **=** -2728.017164
$\boldsymbol{G_{SP}}$ **=** -2727.237686

Si	-0.000082000	1.959119000	0.248768000
N	-2.766316000	1.225363000	-0.050934000
N	-2.901170000	-0.936023000	-0.147189000
C	-3.632680000	0.197268000	-0.132422000
C	-1.424919000	0.751255000	-0.010843000
C	-1.523701000	-0.652445000	-0.055156000
C	-3.153526000	2.606285000	-0.161217000
C	-3.256272000	3.396008000	0.996217000
C	-3.661607000	4.730039000	0.838041000
H	-3.754361000	5.364415000	1.723719000
C	-3.940362000	5.250916000	-0.424742000
H	-4.252789000	6.293313000	-0.527282000
C	-3.817721000	4.446612000	-1.560897000
H	-4.029259000	4.860638000	-2.550136000
C	-3.420421000	3.109347000	-1.450049000
C	-3.456277000	-2.260518000	-0.101651000
C	-3.929065000	-2.738183000	1.134540000
C	-4.460726000	-4.034668000	1.169363000
H	-4.834311000	-4.432278000	2.116482000
C	-4.507972000	-4.818540000	0.015179000
H	-4.924140000	-5.828170000	0.060304000
C	-4.019171000	-4.322707000	-1.195461000
H	-4.052704000	-4.944223000	-2.093975000
C	-3.478143000	-3.032553000	-1.277833000
Sn	-0.000005000	-2.197678000	0.304046000
N	2.901118000	-0.935931000	-0.146795000
N	2.765953000	1.225514000	-0.052549000
C	3.632437000	0.197482000	-0.133156000
C	1.523604000	-0.652465000	-0.055021000
C	1.424629000	0.751251000	-0.011676000
C	3.456658000	-2.260150000	-0.098643000
C	3.479504000	-3.034378000	-1.273347000
C	4.021193000	-4.324087000	-1.188272000
H	4.055502000	-4.947275000	-2.085597000
C	4.509657000	-4.817374000	0.023538000
H	4.926364000	-5.826686000	0.070764000
C	4.461365000	-4.031378000	1.176239000
H	4.834648000	-4.427032000	2.124297000
C	3.929032000	-2.735246000	1.138724000
C	3.153001000	2.606380000	-0.163998000
C	3.419004000	3.108661000	-1.453309000
C	3.816132000	4.445889000	-1.565210000
H	4.027003000	4.859342000	-2.554831000
C	3.939463000	5.250879000	-0.429608000

H	4.251743000	6.293238000	-0.532987000
C	3.661630000	4.730737000	0.833685000
H	3.754975000	5.365632000	1.718930000
C	3.256519000	3.396768000	0.992898000
C	-2.923496000	2.861307000	2.365456000
H	-3.626972000	3.245338000	3.120386000
H	-2.921663000	1.763718000	2.403341000
H	-1.910131000	3.186030000	2.653396000
C	-3.279935000	2.223270000	-2.660969000
H	-3.345177000	2.808016000	-3.589211000
H	-2.315135000	1.691637000	-2.648503000
H	-4.074282000	1.458181000	-2.687930000
C	2.924886000	2.862555000	2.362587000
H	2.922968000	1.764965000	2.400542000
H	3.629140000	3.246620000	3.116774000
H	1.911840000	3.187494000	2.651417000
C	3.277748000	2.221779000	-2.663545000
H	3.343191000	2.805773000	-3.592246000
H	4.071567000	1.456125000	-2.690056000
H	2.312606000	1.690772000	-2.650472000
C	-3.860583000	-1.876485000	2.370084000
H	-4.055100000	-2.469069000	3.275071000
H	-2.872319000	-1.399604000	2.469773000
H	-4.609993000	-1.067905000	2.330439000
C	-2.920003000	-2.497225000	-2.571009000
H	-3.251350000	-1.465690000	-2.769334000
H	-1.818519000	-2.461127000	-2.525045000
H	-3.212326000	-3.128759000	-3.421817000
C	3.859209000	-1.871400000	2.372719000
H	4.053477000	-2.462324000	3.278846000
H	4.608071000	-1.062328000	2.332356000
H	2.870556000	-1.395001000	2.470927000
C	2.921620000	-2.501878000	-2.567803000
H	1.820064000	-2.466893000	-2.522595000
H	3.251980000	-1.470338000	-2.767719000
H	3.215126000	-3.134596000	-3.417327000
C	-5.099542000	0.288521000	-0.190823000
C	-5.800699000	1.094083000	0.726500000
C	-5.825851000	-0.436538000	-1.153874000
C	-7.192802000	1.175054000	0.675600000
C	-7.218322000	-0.353939000	-1.198825000
C	-7.906104000	0.452526000	-0.286461000
H	-5.254650000	1.647049000	1.489538000
H	-5.299552000	-1.060393000	-1.875169000
H	-7.722546000	1.802806000	1.395990000
H	-7.767477000	-0.920748000	-1.954348000
H	-8.996339000	0.516628000	-0.324020000
C	5.099289000	0.288855000	-0.191955000
C	5.825361000	-0.436742000	-1.154775000
C	5.800661000	1.095016000	0.724670000
C	7.217811000	-0.354075000	-1.200209000
C	7.192745000	1.176056000	0.673293000
C	7.905807000	0.453002000	-0.288547000
H	5.298874000	-1.061067000	-1.875525000
H	5.254817000	1.648370000	1.487565000
H	7.766777000	-0.921309000	-1.955549000
H	7.722663000	1.804279000	1.393145000
H	8.996027000	0.517165000	-0.326478000

C	0.000726000	-0.569226000	2.957476000
C	0.000802000	0.665022000	2.880093000
H	0.001023000	1.693292000	3.191914000
H	0.000810000	-1.537988000	3.425900000

TS-GeSn$_{ace}$

E **=** -4518.217276
H **=** -4518.160671
G **=** -4518.310829
***N*$_{imag}$** **=** 1, -244.7 cm^{-1}
SP **=** -4514.586903
G$_{SP}$ **=** -4513.808748

Ge	-0.000431000	2.003133000	0.184661000
N	-2.818055000	1.151050000	-0.085933000
N	-2.917508000	-1.011535000	-0.167064000
C	-3.669202000	0.108057000	-0.147252000
C	-1.472116000	0.702060000	-0.066654000
C	-1.542399000	-0.700963000	-0.097212000
C	-3.225727000	2.526477000	-0.182284000
C	-3.314650000	3.310120000	0.980070000
C	-3.727331000	4.643725000	0.833458000
H	-3.808967000	5.273705000	1.723445000
C	-4.027245000	5.168745000	-0.422255000
H	-4.344792000	6.210455000	-0.515624000
C	-3.918745000	4.369746000	-1.564045000
H	-4.147108000	4.787289000	-2.548027000
C	-3.513274000	3.034302000	-1.465154000
C	-3.449405000	-2.344971000	-0.111347000
C	-3.897654000	-2.828781000	1.131574000
C	-4.408294000	-4.133431000	1.174856000
H	-4.762411000	-4.535880000	2.127405000
C	-4.459102000	-4.919198000	0.022115000
H	-4.858674000	-5.935203000	0.073834000
C	-3.994922000	-4.416997000	-1.195587000
H	-4.030860000	-5.039933000	-2.093024000
C	-3.475334000	-3.118649000	-1.286306000
Sn	-0.001077000	-2.230486000	0.274178000
N	2.917729000	-1.011882000	-0.160280000
N	2.816867000	1.151203000	-0.092718000
C	3.668556000	0.108519000	-0.149931000
C	1.542455000	-0.701572000	-0.091425000
C	1.471235000	0.701480000	-0.067450000
C	3.452512000	-2.343237000	-0.082282000
C	3.483947000	-3.137420000	-1.243136000
C	4.009355000	-4.431694000	-1.128679000
H	4.049903000	-5.069878000	-2.015147000
C	4.473082000	-4.910700000	0.098308000
H	4.877257000	-5.923780000	0.168411000
C	4.415979000	-4.105288000	1.237217000
H	4.769606000	-4.489569000	2.197424000
C	3.900082000	-2.803858000	1.170024000
C	3.224154000	2.526213000	-0.195655000
C	3.506011000	3.030239000	-1.481052000
C	3.911847000	4.365284000	-1.585254000
H	4.135782000	4.780180000	-2.571371000
C	4.026634000	5.166920000	-0.446018000
H	4.344727000	6.208084000	-0.543492000
C	3.733056000	4.645161000	0.812741000
H	3.820486000	5.277203000	1.700679000
C	3.320027000	3.312374000	0.964591000

C	-2.974254000	2.772045000	2.347223000
H	-3.758524000	3.031611000	3.076456000
H	-2.829286000	1.684167000	2.351673000
H	-2.032250000	3.219821000	2.703198000
C	-3.387476000	2.152916000	-2.681142000
H	-3.474261000	2.739572000	-3.606380000
H	-2.418559000	1.628703000	-2.687569000
H	-4.175976000	1.381408000	-2.695114000
C	2.982554000	2.776501000	2.333203000
H	2.890338000	1.682758000	2.350063000
H	3.739902000	3.083283000	3.071965000
H	2.012480000	3.180626000	2.665443000
C	3.373588000	2.145558000	-2.693904000
H	3.460728000	2.728786000	-3.621269000
H	4.158430000	1.370317000	-2.707144000
H	2.402267000	1.625742000	-2.696182000
C	-3.826101000	-1.964523000	2.365073000
H	-3.999722000	-2.558704000	3.273265000
H	-2.843962000	-1.472578000	2.451070000
H	-4.588104000	-1.167469000	2.333980000
C	-2.942819000	-2.575649000	-2.586967000
H	-3.296016000	-1.550923000	-2.783250000
H	-1.841716000	-2.518925000	-2.553883000
H	-3.233244000	-3.214339000	-3.433083000
C	3.822188000	-1.919101000	2.388573000
H	3.996121000	-2.497234000	3.306993000
H	4.581210000	-1.119746000	2.346023000
H	2.837936000	-1.429676000	2.464257000
C	2.947969000	-2.623177000	-2.553911000
H	1.845146000	-2.608183000	-2.532450000
H	3.264536000	-1.587564000	-2.754042000
H	3.270460000	-3.257452000	-3.391807000
C	-5.137924000	0.175110000	-0.181405000
C	-5.836087000	0.969717000	0.747625000
C	-5.868630000	-0.561670000	-1.132109000
C	-7.229967000	1.029302000	0.719796000
C	-7.262806000	-0.500812000	-1.153726000
C	-7.947762000	0.295411000	-0.230241000
H	-5.285564000	1.529817000	1.502245000
H	-5.344313000	-1.177837000	-1.861524000
H	-7.757468000	1.648725000	1.449007000
H	-7.815720000	-1.076642000	-1.899634000
H	-9.039345000	0.342435000	-0.249607000
C	5.137057000	0.176042000	-0.193917000
C	5.860239000	-0.566131000	-1.146249000
C	5.842508000	0.976736000	0.724229000
C	7.254129000	-0.505006000	-1.179847000
C	7.236126000	1.036559000	0.684451000
C	7.946358000	0.297051000	-0.266880000
H	5.329952000	-1.186336000	-1.867919000
H	5.298307000	1.542049000	1.479526000
H	7.801045000	-1.085030000	-1.926928000
H	7.769384000	1.660850000	1.405270000
H	9.037719000	0.344429000	-0.295604000
C	-0.009022000	-0.581857000	2.924557000
C	-0.008761000	0.652409000	2.850427000
H	-0.009907000	1.682927000	3.154020000
H	-0.010384000	-1.550434000	3.393324000

2-Si$_{ace}$

***E* =** -2805.930366
***H* =** -2805.876513
***G* =** -2806.018366
N_{imag} = 0
***SP* =** -2803.464345
G_{SP} = -2802.674189

Si	-0.119187000	1.802991000	1.778428000
N	-2.710701000	0.926933000	0.424407000
N	-2.486735000	-1.225913000	0.270226000
C	-3.326007000	-0.199625000	-0.022528000
C	-1.483423000	0.614379000	1.007699000
C	-1.352593000	-0.761418000	0.938378000
C	-3.192257000	2.267893000	0.221709000
C	-3.706316000	2.977290000	1.321695000
C	-4.166347000	4.281076000	1.092723000
H	-4.575107000	4.855151000	1.928132000
C	-4.101926000	4.850581000	-0.180394000
H	-4.463203000	5.869815000	-0.338919000
C	-3.568780000	4.128467000	-1.249497000
H	-3.506156000	4.584595000	-2.240822000
C	-3.098352000	2.821099000	-1.067495000
C	-2.666933000	-2.593346000	-0.134172000
C	-3.397563000	-3.460359000	0.691035000
C	-3.526200000	-4.792755000	0.274184000
H	-4.087305000	-5.494264000	0.896632000
C	-2.936235000	-5.230147000	-0.913298000
H	-3.040297000	-6.274437000	-1.218353000
C	-2.209152000	-4.343253000	-1.711764000
H	-1.747360000	-4.693206000	-2.638627000
C	-2.060981000	-3.001929000	-1.336202000
Si	0.088210000	-1.803027000	1.819562000
N	2.625471000	-0.957960000	0.331413000
N	2.428806000	1.203605000	0.209000000
C	3.234439000	0.166274000	-0.127884000
C	1.415065000	-0.633457000	0.949890000
C	1.294534000	0.744560000	0.882419000
C	3.285993000	-2.236193000	0.443343000
C	2.915416000	-3.296205000	-0.400541000
C	3.605941000	-4.508664000	-0.263542000
H	3.339479000	-5.347196000	-0.912125000
C	4.613884000	-4.656500000	0.689770000
H	5.140187000	-5.609843000	0.782730000
C	4.942067000	-3.595413000	1.535546000
H	5.717537000	-3.721154000	2.295425000
C	4.280725000	-2.364683000	1.432487000
C	2.633950000	2.567663000	-0.201625000
C	2.296426000	2.917203000	-1.520693000
C	2.503019000	4.246040000	-1.913764000
H	2.248313000	4.547098000	-2.933204000
C	3.020484000	5.182331000	-1.016421000
H	3.175236000	6.215410000	-1.337824000
C	3.332612000	4.808527000	0.292198000
H	3.727450000	5.548918000	0.992549000
C	3.140159000	3.490762000	0.729298000
C	-3.741681000	2.362934000	2.696775000
H	-4.394384000	2.935601000	3.370643000
H	-4.088302000	1.317786000	2.675605000
H	-2.729131000	2.344324000	3.134396000
C	-2.504299000	2.032780000	-2.207056000
H	-2.288028000	2.683243000	-3.066103000
H	-1.568164000	1.539654000	-1.901634000

H	-3.192855000	1.241190000	-2.547409000
C	3.435971000	3.080195000	2.147780000
H	3.962103000	2.114108000	2.199628000
H	4.042431000	3.837919000	2.663540000
H	2.493075000	2.954482000	2.707562000
C	1.731892000	1.888757000	-2.467723000
H	1.326527000	2.363412000	-3.372261000
H	2.506305000	1.168210000	-2.780835000
H	0.926817000	1.309360000	-1.988082000
C	-3.997454000	-2.958587000	1.977738000
H	-4.514395000	-3.763954000	2.517621000
H	-3.214317000	-2.546280000	2.634979000
H	-4.722187000	-2.148988000	1.790180000
C	-1.314481000	-2.011913000	-2.195815000
H	-2.002369000	-1.279253000	-2.651808000
H	-0.590085000	-1.432680000	-1.605195000
H	-0.776592000	-2.518567000	-3.008548000
C	4.612531000	-1.218469000	2.355018000
H	5.211921000	-1.563243000	3.209088000
H	5.192491000	-0.439564000	1.832212000
H	3.698846000	-0.741262000	2.743776000
C	1.783720000	-3.157380000	-1.380385000
H	0.825434000	-3.288674000	-0.849607000
H	1.756499000	-2.162189000	-1.848052000
H	1.843287000	-3.917601000	-2.172609000
C	-4.665683000	-0.284336000	-0.621741000
C	-5.726143000	0.467361000	-0.074485000
C	-4.932052000	-1.110263000	-1.732172000
C	-7.005408000	0.400942000	-0.626418000
C	-6.215753000	-1.177131000	-2.276114000
C	-7.256920000	-0.421076000	-1.729543000
H	-5.551936000	1.098997000	0.795070000
H	-4.136302000	-1.701761000	-2.179233000
H	-7.811984000	0.990831000	-0.184598000
H	-6.399428000	-1.823340000	-3.137799000
H	-8.260176000	-0.474041000	-2.159269000
C	4.497114000	0.231509000	-0.872148000
C	4.767530000	-0.705234000	-1.889544000
C	5.457274000	1.223366000	-0.591819000
C	5.965884000	-0.652058000	-2.601747000
C	6.651247000	1.275704000	-1.312177000
C	6.911409000	0.338827000	-2.317451000
H	4.031480000	-1.472079000	-2.128693000
H	5.274381000	1.950307000	0.198068000
H	6.158585000	-1.386277000	-3.387587000
H	7.385468000	2.050826000	-1.080274000
H	7.848613000	0.380591000	-2.877781000
C	0.046276000	-0.653974000	3.426814000
C	-0.020971000	0.693237000	3.413540000
H	-0.034535000	1.231961000	4.377872000
H	0.088009000	-1.172412000	4.401071000

2-Ge$_{ace}$

E **=** -6380.691050
H **=** -6380.636685
G **=** -6380.778842
***N*$_{imag}$** **=** 0
SP **=** -6376.594347
***G*$_{SP}$** **=** -6375.808194

Ge	0.127831000	1.890292000	1.663504000

N	-2.474861000	1.210395000	0.060536000
N	-2.674825000	-0.947687000	0.187606000
C	-3.281872000	0.174776000	-0.277622000
C	-1.342530000	0.751715000	0.738647000
C	-1.467171000	-0.623636000	0.813157000
C	-2.674618000	2.574593000	-0.349547000
C	-3.184644000	3.497170000	0.579942000
C	-3.365433000	4.817884000	0.146441000
H	-3.763332000	5.557703000	0.845679000
C	-3.038314000	5.195058000	-1.157536000
H	-3.184173000	6.230234000	-1.476324000
C	-2.517008000	4.259408000	-2.053394000
H	-2.250429000	4.563126000	-3.069000000
C	-2.321416000	2.927924000	-1.663525000
C	-3.329198000	-2.228572000	0.291164000
C	-4.327051000	-2.368220000	1.275781000
C	-4.976355000	-3.605621000	1.375137000
H	-5.753777000	-3.739808000	2.131581000
C	-4.634173000	-4.662659000	0.529690000
H	-5.151066000	-5.621399000	0.619878000
C	-3.625258000	-4.503269000	-0.420612000
H	-3.349449000	-5.337799000	-1.070503000
C	-2.946549000	-3.283566000	-0.553871000
Ge	-0.095986000	-1.873624000	1.723498000
N	2.524656000	-1.229494000	0.117224000
N	2.761160000	0.918784000	0.275056000
C	3.368360000	-0.208517000	-0.180260000
C	1.396005000	-0.760621000	0.792653000
C	1.535217000	0.612118000	0.865865000
C	2.694759000	-2.599499000	-0.280208000
C	2.067542000	-3.016217000	-1.468547000
C	2.200862000	-4.362267000	-1.832828000
H	1.722713000	-4.718235000	-2.749047000
C	2.934412000	-5.246103000	-1.036786000
H	3.026653000	-6.294106000	-1.332790000
C	3.545844000	-4.800878000	0.136871000
H	4.112044000	-5.499926000	0.757495000
C	3.432510000	-3.463399000	0.542105000
C	3.246066000	2.258244000	0.076539000
C	3.134772000	2.823719000	-1.205837000
C	3.603101000	4.132774000	-1.381820000
H	3.527405000	4.598315000	-2.367852000
C	4.150440000	4.844732000	-0.313052000
H	4.509599000	5.865463000	-0.466659000
C	4.231924000	4.263214000	0.953773000
H	4.652359000	4.829338000	1.788828000
C	3.775046000	2.957137000	1.176351000
C	-3.499453000	3.082431000	1.993680000
H	-4.107332000	3.842041000	2.504918000
H	-4.033059000	2.119998000	2.034627000
H	-2.567248000	2.947000000	2.567863000
C	-1.750306000	1.900050000	-2.607134000
H	-1.345588000	2.374303000	-3.512174000
H	-0.943522000	1.326081000	-2.123610000
H	-2.520584000	1.174769000	-2.919387000
C	3.833415000	2.327045000	2.544061000
H	4.196296000	1.287856000	2.505214000
H	4.485760000	2.900947000	3.217157000
H	2.828308000	2.284706000	2.995704000
C	2.524517000	2.045282000	-2.343653000
H	2.298924000	2.702433000	-3.195194000

H	3.207004000	1.254532000	-2.698040000
H	1.591447000	1.551625000	-2.029527000
C	-4.675593000	-1.225081000	2.195854000
H	-5.277435000	-1.574980000	3.046132000
H	-3.768966000	-0.738917000	2.589787000
H	-5.259108000	-0.452036000	1.668229000
C	-1.817609000	-3.132046000	-1.535930000
H	-1.783113000	-2.126491000	-1.979817000
H	-0.856403000	-3.285394000	-1.017593000
H	-1.889052000	-3.872835000	-2.345474000
C	4.059528000	-2.951898000	1.812249000
H	4.556324000	-3.760748000	2.365675000
H	4.808158000	-2.170770000	1.598647000
H	3.297995000	-2.498377000	2.467351000
C	1.313656000	-2.028923000	-2.324630000
H	0.592228000	-1.451042000	-1.728935000
H	1.997197000	-1.294569000	-2.784338000
H	0.772096000	-2.537208000	-3.133934000
C	-4.540709000	0.239878000	-1.028424000
C	-5.501521000	1.233116000	-0.754921000
C	-4.807271000	-0.698059000	-2.045870000
C	-6.691838000	1.285550000	-1.481345000
C	-6.002064000	-0.644934000	-2.764097000
C	-6.948130000	0.347258000	-2.486284000
H	-5.321977000	1.961267000	0.034595000
H	-4.070776000	-1.465843000	-2.280520000
H	-7.426331000	2.061925000	-1.254442000
H	-6.191466000	-1.380256000	-3.549739000
H	-7.882525000	0.388973000	-3.051291000
C	4.699528000	-0.299254000	-0.796595000
C	4.946475000	-1.129948000	-1.908196000
C	5.771044000	0.450307000	-0.268142000
C	6.221708000	-1.204075000	-2.470723000
C	7.041736000	0.376826000	-0.838787000
C	7.273918000	-0.450321000	-1.942318000
H	4.141479000	-1.719194000	-2.341689000
H	5.612342000	1.086240000	0.601199000
H	6.390065000	-1.853956000	-3.332802000
H	7.857006000	0.965356000	-0.411286000
H	8.270542000	-0.508882000	-2.386517000
C	-0.041318000	-0.634222000	3.370961000
C	0.023832000	0.708404000	3.352337000
H	0.037773000	1.256881000	4.311550000
H	-0.079880000	-1.154557000	4.344781000

2-Sn$_{ace}$

E **=** -2655.857733
H **=** -2655.801507
G **=** -2655.950957
$\boldsymbol{N_{imag}}$ **=** 0
SP **=** -2652.709137
$\boldsymbol{G_{SP}}$ **=** -2651.928105

Sn	0.177698000	2.068156000	-1.670547000
N	2.903838000	0.858342000	-0.173586000
N	2.585552000	-1.272644000	0.013994000
C	3.447784000	-0.276318000	0.335381000
C	1.694604000	0.584420000	-0.821407000
C	1.503001000	-0.782462000	-0.727100000
C	3.422025000	2.184981000	0.020669000
C	4.035037000	2.838180000	-1.063823000

C	4.513394000	4.138140000	-0.850420000
H	4.998282000	4.668780000	-1.673759000
C	4.372901000	4.758145000	0.392864000
H	4.749576000	5.773552000	0.539496000
C	3.744404000	4.091475000	1.446072000
H	3.623360000	4.587177000	2.412680000
C	3.251453000	2.790344000	1.278274000
C	2.681001000	-2.636074000	0.454259000
C	3.447327000	-3.546453000	-0.288292000
C	3.477747000	-4.877294000	0.152805000
H	4.063149000	-5.611125000	-0.406880000
C	2.762716000	-5.271350000	1.285331000
H	2.791722000	-6.314580000	1.609722000
C	2.007569000	-4.341336000	2.005160000
H	1.450488000	-4.656399000	2.891277000
C	1.953758000	-3.000724000	1.602786000
Sn	-0.137208000	-2.006422000	-1.794938000
N	-2.791363000	-0.898103000	-0.075089000
N	-2.538918000	1.247269000	0.091513000
C	-3.356343000	0.223926000	0.436917000
C	-1.598741000	-0.589055000	-0.744470000
C	-1.436935000	0.782652000	-0.639623000
C	-3.470140000	-2.165818000	-0.171135000
C	-3.089947000	-3.233031000	0.660032000
C	-3.785298000	-4.443610000	0.525448000
H	-3.511043000	-5.286928000	1.164631000
C	-4.809404000	-4.582522000	-0.411259000
H	-5.338469000	-5.534434000	-0.503040000
C	-5.154008000	-3.512244000	-1.239283000
H	-5.946861000	-3.628963000	-1.982490000
C	-4.488909000	-2.283715000	-1.137797000
C	-2.686890000	2.602550000	0.548112000
C	-2.251075000	2.910369000	1.849517000
C	-2.384317000	4.235254000	2.284495000
H	-2.052537000	4.502945000	3.290982000
C	-2.926788000	5.210115000	1.444372000
H	-3.023291000	6.239826000	1.797691000
C	-3.339257000	4.878068000	0.152756000
H	-3.755636000	5.647511000	-0.502397000
C	-3.222648000	3.564998000	-0.324897000
C	4.161891000	2.165100000	-2.406814000
H	4.846332000	2.719521000	-3.064097000
H	4.526819000	1.129745000	-2.315168000
H	3.183256000	2.099022000	-2.909810000
C	2.552942000	2.061009000	2.397620000
H	2.303598000	2.745842000	3.220199000
H	1.622140000	1.591056000	2.042981000
H	3.187326000	1.256841000	2.806846000
C	-3.632602000	3.204171000	-1.729654000
H	-4.139387000	2.228416000	-1.777968000
H	-4.298145000	3.967439000	-2.157089000
H	-2.746286000	3.125901000	-2.381816000
C	-1.659305000	1.840466000	2.731777000
H	-1.202861000	2.275890000	3.631701000
H	-2.430109000	1.120921000	3.055828000
H	-0.889210000	1.266088000	2.192098000
C	4.198166000	-3.088738000	-1.511099000
H	4.679571000	-3.933449000	-2.023012000
H	3.521670000	-2.589197000	-2.223118000
H	4.980048000	-2.358044000	-1.244058000
C	1.178087000	-1.964956000	2.378190000

H	1.851029000	-1.210637000	2.820388000
H	0.483454000	-1.414776000	1.726428000
H	0.604775000	-2.426070000	3.193735000
C	-4.844418000	-1.123678000	-2.033411000
H	-5.483473000	-1.450244000	-2.865580000
H	-5.391494000	-0.344814000	-1.476026000
H	-3.941779000	-0.651639000	-2.452758000
C	-1.951672000	-3.106642000	1.636120000
H	-0.997172000	-3.330318000	1.131001000
H	-1.863652000	-2.090197000	2.044689000
H	-2.062372000	-3.814215000	2.470885000
C	4.735740000	-0.397330000	1.033190000
C	5.864174000	0.301017000	0.556056000
C	4.882373000	-1.206748000	2.177806000
C	7.093535000	0.197919000	1.207241000
C	6.116450000	-1.310407000	2.821552000
C	7.226276000	-0.607912000	2.342524000
H	5.782755000	0.920177000	-0.335807000
H	4.030397000	-1.755863000	2.573084000
H	7.954722000	0.746662000	0.818594000
H	6.207206000	-1.942727000	3.708023000
H	8.190506000	-0.689422000	2.850089000
C	-4.587801000	0.300775000	1.232161000
C	-4.833687000	-0.640502000	2.251487000
C	-5.542618000	1.309724000	0.998682000
C	-6.002557000	-0.575413000	3.010419000
C	-6.707170000	1.373830000	1.764928000
C	-6.943040000	0.432028000	2.771543000
H	-4.101137000	-1.420339000	2.456123000
H	-5.379158000	2.040989000	0.208672000
H	-6.175951000	-1.313650000	3.797041000
H	-7.437506000	2.162296000	1.568264000
H	-7.857153000	0.482893000	3.368096000
C	-0.069098000	-0.578586000	-3.545537000
C	0.010855000	0.762855000	-3.509708000
H	0.016522000	1.313583000	-4.470342000
H	-0.125549000	-1.073351000	-4.534533000

2-SiGe$_{ace}$

***E* =** -4593.310960
***H* =** -4593.256309
***G* =** -4593.400808
N_{imag} = 0
***SP* =** -4590.029657
G_{SP} = -4589.242419

Si	-0.108223000	1.777866000	1.756552000
N	-2.699077000	0.994963000	0.366236000
N	-2.533315000	-1.155148000	0.138434000
C	-3.345860000	-0.097275000	-0.116763000
C	-1.476588000	0.629606000	0.934848000
C	-1.384966000	-0.744969000	0.814260000
C	-3.148501000	2.353326000	0.212499000
C	-3.652018000	3.032579000	1.336031000
C	-4.083288000	4.353414000	1.153568000
H	-4.482970000	4.905145000	2.008223000
C	-4.001537000	4.967817000	-0.097535000
H	-4.340231000	5.999764000	-0.219546000
C	-3.480091000	4.273965000	-1.190853000
H	-3.404151000	4.764462000	-2.164699000
C	-3.038808000	2.950900000	-1.055364000

C	-2.763078000	-2.510314000	-0.280096000
C	-3.530054000	-3.354196000	0.536945000
C	-3.703373000	-4.679836000	0.114744000
H	-4.293968000	-5.362742000	0.730595000
C	-3.120768000	-5.134129000	-1.070058000
H	-3.259654000	-6.173056000	-1.379527000
C	-2.356272000	-4.271244000	-1.859637000
H	-1.900325000	-4.634602000	-2.784251000
C	-2.162627000	-2.937051000	-1.478600000
Ge	0.087536000	-1.907727000	1.719762000
N	2.673050000	-0.903963000	0.222778000
N	2.443288000	1.255839000	0.188406000
C	3.262900000	0.247042000	-0.194620000
C	1.464424000	-0.624620000	0.862592000
C	1.318528000	0.751588000	0.850233000
C	3.339469000	-2.182072000	0.261144000
C	2.952487000	-3.200780000	-0.625615000
C	3.639865000	-4.420780000	-0.555238000
H	3.360940000	-5.227300000	-1.238271000
C	4.661985000	-4.615481000	0.374253000
H	5.185838000	-5.573803000	0.415088000
C	5.008744000	-3.594830000	1.261450000
H	5.796773000	-3.757387000	2.001163000
C	4.350429000	-2.358616000	1.226119000
C	2.626200000	2.638417000	-0.166905000
C	2.277389000	3.037263000	-1.468865000
C	2.461418000	4.384407000	-1.807059000
H	2.196637000	4.723746000	-2.811804000
C	2.968676000	5.290540000	-0.873718000
H	3.105435000	6.338502000	-1.152264000
C	3.293393000	4.867361000	0.416686000
H	3.680008000	5.584001000	1.145755000
C	3.123246000	3.529792000	0.799138000
C	-3.706119000	2.368976000	2.687337000
H	-4.347129000	2.931699000	3.380546000
H	-4.077290000	1.333805000	2.626678000
H	-2.695374000	2.310096000	3.125641000
C	-2.459145000	2.190771000	-2.221123000
H	-2.221352000	2.867069000	-3.054202000
H	-1.537945000	1.662205000	-1.929980000
H	-3.166110000	1.430569000	-2.593871000
C	3.431618000	3.065054000	2.197947000
H	3.978434000	2.109090000	2.207046000
H	4.023211000	3.812592000	2.744899000
H	2.492903000	2.894863000	2.752904000
C	1.723508000	2.040493000	-2.455119000
H	1.304863000	2.545888000	-3.336707000
H	2.507282000	1.346248000	-2.802465000
H	0.930964000	1.428820000	-1.995269000
C	-4.126141000	-2.834510000	1.818835000
H	-4.641026000	-3.632623000	2.371271000
H	-3.344416000	-2.409290000	2.469067000
H	-4.853468000	-2.029556000	1.620315000
C	-1.374338000	-1.973018000	-2.330271000
H	-2.034143000	-1.225178000	-2.803122000
H	-0.645406000	-1.409900000	-1.730177000
H	-0.836440000	-2.499045000	-3.130705000
C	4.703815000	-1.255954000	2.192508000
H	5.314844000	-1.640897000	3.020804000
H	5.279554000	-0.457939000	1.694408000
H	3.799456000	-0.791091000	2.616136000

C	1.811803000	-3.011011000	-1.587482000
H	0.856779000	-3.185173000	-1.064630000
H	1.772019000	-1.988415000	-1.990834000
H	1.873954000	-3.718786000	-2.426749000
C	-4.685439000	-0.128175000	-0.720512000
C	-5.730616000	0.629895000	-0.153292000
C	-4.965713000	-0.912626000	-1.857206000
C	-7.008897000	0.609170000	-0.711239000
C	-6.248382000	-0.934215000	-2.407106000
C	-7.274497000	-0.172538000	-1.840116000
H	-5.545305000	1.230666000	0.735615000
H	-4.180347000	-1.506102000	-2.320063000
H	-7.803878000	1.203137000	-0.254107000
H	-6.443145000	-1.548778000	-3.289274000
H	-8.277096000	-0.189688000	-2.274279000
C	4.519942000	0.361050000	-0.942616000
C	4.796864000	-0.527949000	-2.000308000
C	5.468668000	1.352782000	-0.625332000
C	5.990339000	-0.428504000	-2.715849000
C	6.657661000	1.451734000	-1.349045000
C	6.924365000	0.561989000	-2.394656000
H	4.069506000	-1.293593000	-2.268459000
H	5.280516000	2.042828000	0.195862000
H	6.188040000	-1.125895000	-3.533363000
H	7.382899000	2.226164000	-1.088293000
H	7.857618000	0.640165000	-2.957674000
C	0.023326000	-0.697971000	3.391781000
C	-0.036424000	0.647255000	3.374987000
H	-0.057180000	1.182856000	4.341704000
H	0.051092000	-1.225355000	4.361496000

2-SiSn$_{ace}$

E **=** -2730.896125
H **=** -2730.840897
G **=** -2730.987673
***N*$_{imag}$** **=** 0
SP **=** -2728.088537
***G*$_{SP}$** **=** -2727.303846

Si	0.073351000	1.664781000	-1.752889000
N	2.659182000	1.073973000	-0.313994000
N	2.652364000	-1.067116000	0.015356000
C	3.383907000	0.058111000	0.216305000
C	1.460387000	0.592884000	-0.857994000
C	1.468759000	-0.778091000	-0.665131000
C	3.020238000	2.464487000	-0.230320000
C	3.503580000	3.110214000	-1.381996000
C	3.851994000	4.462560000	-1.266947000
H	4.232829000	4.990258000	-2.145071000
C	3.711746000	5.138086000	-0.052961000
H	3.985839000	6.193840000	0.016406000
C	3.214312000	4.474307000	1.069800000
H	3.093271000	5.011146000	2.014220000
C	2.855109000	3.121614000	1.001149000
C	3.030113000	-2.392366000	0.420925000
C	3.912963000	-3.122092000	-0.391380000
C	4.238820000	-4.424385000	0.013332000
H	4.921699000	-5.017934000	-0.599753000
C	3.693197000	-4.968389000	1.177613000
H	3.952973000	-5.987625000	1.474392000
C	2.811535000	-4.220442000	1.962044000

H	2.385246000	-4.653672000	2.870525000
C	2.461955000	-2.913688000	1.597403000
Sn	-0.078733000	-2.179099000	-1.645050000
N	-2.792641000	-0.870645000	-0.102233000
N	-2.479676000	1.273204000	-0.205340000
C	-3.333571000	0.324957000	0.246209000
C	-1.582598000	-0.685918000	-0.774556000
C	-1.379225000	0.682508000	-0.844874000
C	-3.485550000	-2.129518000	-0.010902000
C	-3.098616000	-3.067330000	0.961400000
C	-3.802932000	-4.278785000	1.014251000
H	-3.524696000	-5.022772000	1.765231000
C	-4.842776000	-4.542838000	0.122367000
H	-5.379543000	-5.493239000	0.177680000
C	-5.193534000	-3.601300000	-0.847325000
H	-5.998586000	-3.818108000	-1.554067000
C	-4.518775000	-2.376749000	-0.935954000
C	-2.609428000	2.679702000	0.072330000
C	-2.241856000	3.136354000	1.349876000
C	-2.379147000	4.505085000	1.615713000
H	-2.097393000	4.888857000	2.599677000
C	-2.861967000	5.376015000	0.637147000
H	-2.962617000	6.441420000	0.859278000
C	-3.208609000	4.894761000	-0.627043000
H	-3.575817000	5.583508000	-1.392146000
C	-3.083477000	3.534114000	-0.937761000
C	3.621348000	2.376876000	-2.692461000
H	4.224848000	2.946045000	-3.413499000
H	4.069919000	1.378263000	-2.569729000
H	2.622280000	2.215145000	-3.131900000
C	2.304378000	2.389709000	2.198296000
H	2.020104000	3.092067000	2.994500000
H	1.416953000	1.798034000	1.925064000
H	3.047110000	1.688310000	2.614473000
C	-3.410035000	3.005616000	-2.309104000
H	-3.984469000	2.066827000	-2.267076000
H	-3.979947000	3.740378000	-2.895099000
H	-2.476575000	2.778228000	-2.852731000
C	-1.719770000	2.176260000	2.388348000
H	-1.250362000	2.713554000	3.224378000
H	-2.533068000	1.554117000	2.799110000
H	-0.976050000	1.490025000	1.953635000
C	4.478489000	-2.510474000	-1.647004000
H	5.036098000	-3.252833000	-2.234671000
H	3.678465000	-2.093609000	-2.279617000
H	5.164178000	-1.680369000	-1.407226000
C	1.537138000	-2.073459000	2.441765000
H	2.074344000	-1.227225000	2.904303000
H	0.732879000	-1.632726000	1.836073000
H	1.084758000	-2.667082000	3.248108000
C	-4.880005000	-1.356472000	-1.985985000
H	-5.517196000	-1.800785000	-2.763302000
H	-5.430876000	-0.509149000	-1.544224000
H	-3.979974000	-0.943390000	-2.468272000
C	-1.945008000	-2.805961000	1.892678000
H	-0.999612000	-3.094566000	1.404990000
H	-1.852916000	-1.742241000	2.157245000
H	-2.038395000	-3.393456000	2.817662000
C	4.711343000	0.152692000	0.839364000
C	5.718332000	0.944534000	0.251031000
C	5.012555000	-0.549130000	2.023340000

C	6.982387000	1.034985000	0.834358000
C	6.280756000	-0.459607000	2.599046000
C	7.269934000	0.333677000	2.009763000
H	5.514745000	1.484367000	-0.672366000
H	4.252250000	-1.163211000	2.502055000
H	7.749153000	1.653228000	0.361490000
H	6.493474000	-1.010859000	3.518117000
H	8.261423000	0.404006000	2.463669000
C	-4.581833000	0.536570000	0.989019000
C	-4.879253000	-0.255158000	2.115828000
C	-5.502015000	1.527403000	0.594868000
C	-6.065600000	-0.062247000	2.824369000
C	-6.684034000	1.720148000	1.311129000
C	-6.971501000	0.926327000	2.426212000
H	-4.173619000	-1.018096000	2.443230000
H	-5.296449000	2.142751000	-0.279850000
H	-6.279583000	-0.684832000	3.696357000
H	-7.387389000	2.492307000	0.990544000
H	-7.899152000	1.077611000	2.983576000
C	0.018645000	-0.804871000	-3.444210000
C	0.058040000	0.539391000	-3.371304000
H	0.090246000	1.106219000	-4.321742000
H	0.019266000	-1.284651000	-4.439616000

2-GeSn$_{ace}$

E **=** -4518.275167
H **=** -4518.219350
G **=** -4518.367552
***N*$_{imag}$** **=** 0
SP **=** -4514.653202
***G*$_{SP}$** **=** -4513.870514

Ge	0.089005000	1.783192000	-1.665699000
N	2.720650000	1.008184000	-0.216263000
N	2.648566000	-1.139626000	0.045748000
C	3.411247000	-0.044621000	0.289644000
C	1.515447000	0.580794000	-0.783785000
C	1.479475000	-0.794155000	-0.638050000
C	3.114664000	2.386060000	-0.093410000
C	3.623185000	3.048482000	-1.224698000
C	3.993063000	4.391926000	-1.075465000
H	4.394665000	4.932065000	-1.936602000
C	3.848828000	5.042759000	0.151488000
H	4.140116000	6.091762000	0.247722000
C	3.325426000	4.363386000	1.253031000
H	3.201227000	4.881639000	2.207354000
C	2.943623000	3.019073000	1.149849000
C	2.973881000	-2.485915000	0.426153000
C	3.836574000	-3.231498000	-0.392975000
C	4.107304000	-4.554043000	-0.014019000
H	4.773255000	-5.160311000	-0.633247000
C	3.527959000	-5.101652000	1.132109000
H	3.744206000	-6.136710000	1.408344000
C	2.668385000	-4.336797000	1.924790000
H	2.216289000	-4.772540000	2.819492000
C	2.374249000	-3.009611000	1.586007000
Sn	-0.085381000	-2.142152000	-1.661539000
N	-2.797056000	-0.918461000	-0.065425000
N	-2.517601000	1.231415000	-0.082238000
C	-3.356732000	0.252226000	0.332199000
C	-1.588807000	-0.686650000	-0.731198000

C	-1.409708000	0.685747000	-0.743714000
C	-3.476385000	-2.187914000	-0.035396000
C	-3.084870000	-3.166883000	0.893422000
C	-3.777640000	-4.386179000	0.885810000
H	-3.495209000	-5.161934000	1.602292000
C	-4.810948000	-4.618036000	-0.022296000
H	-5.338645000	-5.575075000	-0.014274000
C	-5.166383000	-3.635368000	-0.948478000
H	-5.966050000	-3.826327000	-1.668676000
C	-4.503144000	-2.401758000	-0.976116000
C	-2.665063000	2.624642000	0.244812000
C	-2.288035000	3.044311000	1.532397000
C	-2.432270000	4.403121000	1.841974000
H	-2.144503000	4.758098000	2.834942000
C	-2.929117000	5.300853000	0.895036000
H	-3.034644000	6.358126000	1.151064000
C	-3.283326000	4.856932000	-0.380539000
H	-3.662034000	5.566655000	-1.120457000
C	-3.152808000	3.507066000	-0.734280000
C	3.749537000	2.340323000	-2.548898000
H	4.366661000	2.918855000	-3.250617000
H	4.189951000	1.336473000	-2.440133000
H	2.757550000	2.191973000	-3.007141000
C	2.360953000	2.270282000	2.321214000
H	2.091440000	2.957485000	3.135503000
H	1.458352000	1.714073000	2.023247000
H	3.078178000	1.534240000	2.721849000
C	-3.493596000	3.019972000	-2.118319000
H	-4.057410000	2.074276000	-2.098614000
H	-4.081340000	3.767228000	-2.669806000
H	-2.570478000	2.820824000	-2.688723000
C	-1.746908000	2.055820000	2.533825000
H	-1.296943000	2.569694000	3.394865000
H	-2.545548000	1.395427000	2.912103000
H	-0.982668000	1.408358000	2.075417000
C	4.438211000	-2.614596000	-1.628965000
H	4.978469000	-3.363314000	-2.224725000
H	3.660204000	-2.159232000	-2.262522000
H	5.147372000	-1.812301000	-1.364058000
C	1.476812000	-2.150948000	2.441536000
H	2.045267000	-1.340202000	2.929543000
H	0.698019000	-1.661979000	1.839394000
H	0.991690000	-2.743760000	3.229185000
C	-4.869946000	-1.337088000	-1.979115000
H	-5.498784000	-1.750962000	-2.779696000
H	-5.432043000	-0.517380000	-1.500838000
H	-3.971892000	-0.892947000	-2.436886000
C	-1.937497000	-2.940102000	1.841072000
H	-0.988882000	-3.208528000	1.347999000
H	-1.848946000	-1.887386000	2.147061000
H	-2.035504000	-3.563230000	2.742045000
C	4.734207000	-0.006489000	0.927836000
C	5.766347000	0.780682000	0.377615000
C	5.007015000	-0.756590000	2.089133000
C	7.026400000	0.819451000	0.975145000
C	6.271339000	-0.718737000	2.678867000
C	7.285462000	0.070124000	2.127267000
H	5.585856000	1.357568000	-0.527929000
H	4.227854000	-1.368456000	2.539305000
H	7.812523000	1.434909000	0.531296000
H	6.461364000	-1.307395000	3.579542000

H	8.273891000	0.099742000	2.592172000
C	-4.604787000	0.414832000	1.087211000
C	-4.888042000	-0.429247000	2.179303000
C	-5.538476000	1.411253000	0.741469000
C	-6.073230000	-0.281892000	2.900537000
C	-6.719257000	1.558267000	1.470540000
C	-6.992456000	0.712613000	2.550516000
H	-4.172060000	-1.197371000	2.469987000
H	-5.344956000	2.067035000	-0.106163000
H	-6.275895000	-0.944937000	3.744987000
H	-7.432922000	2.335584000	1.187341000
H	-7.919166000	0.828229000	3.117887000
C	0.006567000	-0.733965000	-3.432324000
C	0.050666000	0.607455000	-3.357182000
H	0.077829000	1.188556000	-4.298688000
H	-0.001079000	-1.208046000	-4.431076000

TS-Si$_{H2}$

***E* =** -2729.773684
***H* =** -2729.721355
***G* =** -2729.861116
N_{imag} = 1, -387.5 cm^{-1}
***SP* =** -2727.364910
G_{SP} = -2726.593799

Si	0.000031000	1.891941000	1.048077000
N	-2.721147000	1.091386000	0.196308000
N	-2.703229000	-1.080016000	0.178094000
C	-3.496561000	0.001105000	0.007368000
C	-1.394546000	0.714590000	0.479538000
C	-1.387062000	-0.687198000	0.486044000
C	-3.165168000	2.446716000	0.007673000
C	-3.476457000	3.225957000	1.135704000
C	-3.913270000	4.539489000	0.913158000
H	-4.165434000	5.168451000	1.770787000
C	-4.024462000	5.048301000	-0.381953000
H	-4.365338000	6.075440000	-0.534868000
C	-3.697093000	4.253910000	-1.482851000
H	-3.776531000	4.660361000	-2.494390000
C	-3.257435000	2.935436000	-1.307774000
C	-3.170786000	-2.440233000	0.169162000
C	-3.866957000	-2.911716000	1.295926000
C	-4.327184000	-4.235395000	1.268168000
H	-4.869096000	-4.629209000	2.131801000
C	-4.090784000	-5.050934000	0.160311000
H	-4.454559000	-6.081549000	0.156147000
C	-3.384104000	-4.559306000	-0.939617000
H	-3.195112000	-5.204858000	-1.801187000
C	-2.906516000	-3.242631000	-0.955381000
Si	0.000025000	-1.843186000	1.127371000
N	2.703244000	-1.080027000	0.178005000
N	2.721170000	1.091374000	0.196214000
C	3.496578000	0.001090000	0.007265000
C	1.387083000	-0.687203000	0.485974000
C	1.394573000	0.714584000	0.479467000
C	3.170785000	-2.440251000	0.169120000
C	2.906522000	-3.242691000	-0.955393000
C	3.384043000	-4.559391000	-0.939542000
H	3.195052000	-5.204975000	-1.801089000
C	4.090648000	-5.051003000	0.160439000
H	4.454369000	-6.081637000	0.156341000

C	4.327041000	-4.235422000	1.268267000
H	4.868891000	-4.629222000	2.131945000
C	3.866881000	-2.911720000	1.295940000
C	3.165205000	2.446714000	0.007699000
C	3.257481000	2.935556000	-1.307704000
C	3.697099000	4.254058000	-1.482656000
H	3.776541000	4.660602000	-2.494157000
C	4.024422000	5.048363000	-0.381680000
H	4.365263000	6.075528000	-0.534498000
C	3.913240000	4.539428000	0.913381000
H	4.165384000	5.168316000	1.771069000
C	3.476474000	3.225857000	1.135803000
C	-3.325946000	2.677102000	2.530884000
H	-2.261560000	2.663748000	2.821257000
H	-3.873731000	3.290612000	3.260219000
H	-3.680766000	1.637587000	2.609158000
C	-2.887267000	2.059562000	-2.476991000
H	-2.859002000	2.636890000	-3.411672000
H	-1.899704000	1.595973000	-2.321528000
H	-3.614051000	1.240026000	-2.607090000
C	3.325982000	2.676894000	2.530945000
H	3.680225000	1.637169000	2.609000000
H	3.874281000	3.290001000	3.260235000
H	2.261655000	2.664100000	2.821564000
C	2.887355000	2.059772000	-2.477001000
H	2.859281000	2.637137000	-3.411666000
H	3.614051000	1.240148000	-2.607035000
H	1.899715000	1.596301000	-2.321682000
C	-4.099807000	-2.017487000	2.486908000
H	-4.453391000	-2.595874000	3.351829000
H	-3.174838000	-1.492797000	2.773978000
H	-4.857156000	-1.247005000	2.263824000
C	-2.121132000	-2.703055000	-2.120735000
H	-2.491351000	-1.718713000	-2.449645000
H	-1.069477000	-2.552995000	-1.827049000
H	-2.150183000	-3.391992000	-2.976500000
C	4.099698000	-2.017458000	2.486904000
H	4.453313000	-2.595813000	3.351833000
H	4.857008000	-1.246943000	2.263805000
H	3.174704000	-1.492807000	2.773971000
C	2.121170000	-2.703178000	-2.120798000
H	1.069429000	-2.553437000	-1.827253000
H	2.491170000	-1.718701000	-2.449543000
H	2.150525000	-3.392011000	-2.976638000
C	-4.924662000	-0.008960000	-0.345460000
C	-5.840355000	0.808327000	0.344504000
C	-5.400753000	-0.838019000	-1.379239000
C	-7.194055000	0.797092000	0.005566000
C	-6.755997000	-0.848323000	-1.711408000
C	-7.657149000	-0.030778000	-1.021916000
H	-5.495039000	1.447566000	1.155400000
H	-4.707951000	-1.471513000	-1.931057000
H	-7.890827000	1.436342000	0.552803000
H	-7.107635000	-1.496612000	-2.517458000
H	-8.717880000	-0.039323000	-1.284329000
C	4.924680000	-0.008983000	-0.345552000
C	5.400768000	-0.838039000	-1.379335000
C	5.840375000	0.808282000	0.344434000
C	6.756016000	-0.848358000	-1.711490000
C	7.194079000	0.797032000	0.005509000
C	7.657170000	-0.030832000	-1.021979000

H	4.707959000	-1.471520000	-1.931161000
H	5.495055000	1.447514000	1.155336000
H	7.107655000	-1.496643000	-2.517542000
H	7.890855000	1.436264000	0.552762000
H	8.717904000	-0.039388000	-1.284381000
H	0.000056000	0.546988000	2.443177000
H	0.000076000	-0.449019000	2.464937000

TS-Ge$_{H2}$

***E* =** -6304.543628
***H* =** -6304.490147
***G* =** -6304.632579
***N*$_{imag}$ =** 1, -824.2 cm^{-1}
***SP* =** -6300.502080
***G*$_{SP}$ =** -6299.735955

Ge	-0.000010000	-1.950377000	-1.126429000
N	-2.759848000	-1.087062000	-0.122715000
N	-2.741799000	1.081676000	-0.100059000
C	-3.530751000	0.001159000	0.103452000
C	-1.451116000	-0.707136000	-0.463999000
C	-1.443467000	0.688349000	-0.465955000
C	-3.190353000	-2.444947000	0.076273000
C	-3.544398000	-3.219164000	-1.042626000
C	-3.960525000	-4.537839000	-0.810686000
H	-4.245334000	-5.162945000	-1.660890000
C	-4.010755000	-5.056417000	0.484372000
H	-4.336224000	-6.087415000	0.644641000
C	-3.641852000	-4.266836000	1.575536000
H	-3.673612000	-4.680806000	2.586652000
C	-3.220951000	-2.943511000	1.390812000
C	-3.205886000	2.442741000	-0.076042000
C	-3.942076000	2.914886000	-1.176698000
C	-4.390380000	4.242532000	-1.136785000
H	-4.962719000	4.637019000	-1.980239000
C	-4.104349000	5.060843000	-0.042805000
H	-4.459052000	6.094529000	-0.029444000
C	-3.359894000	4.567919000	1.031381000
H	-3.132550000	5.215341000	1.882196000
C	-2.892919000	3.247368000	1.034128000
Ge	0.000009000	1.909941000	-1.202157000
N	2.741814000	1.081651000	-0.100067000
N	2.759807000	-1.087089000	-0.122654000
C	3.530736000	0.001118000	0.103483000
C	1.443470000	0.688345000	-0.465942000
C	1.451085000	-0.707140000	-0.463951000
C	3.205973000	2.442694000	-0.076174000
C	2.893010000	3.247461000	1.033896000
C	3.360054000	4.567988000	1.031018000
H	3.132716000	5.215518000	1.881753000
C	4.104579000	5.060753000	-0.043192000
H	4.459335000	6.094422000	-0.029931000
C	4.390616000	4.242304000	-1.137065000
H	4.963014000	4.636665000	-1.980538000
C	3.942242000	2.914676000	-1.176849000
C	3.190295000	-2.444988000	0.076280000
C	3.220844000	-2.943639000	1.390786000
C	3.641707000	-4.266987000	1.575431000
H	3.673430000	-4.681023000	2.586522000
C	4.010615000	-5.056507000	0.484225000
H	4.336048000	-6.087526000	0.644434000

C	3.960437000	-4.537841000	-0.810800000
H	4.245249000	-5.162898000	-1.661039000
C	3.544355000	-3.219138000	-1.042660000
C	-3.461850000	-2.659331000	-2.439592000
H	-2.414901000	-2.644287000	-2.786516000
H	-4.046075000	-3.266903000	-3.145210000
H	-3.819504000	-1.619313000	-2.491903000
C	-2.805804000	-2.072591000	2.548610000
H	-2.733135000	-2.655539000	3.477386000
H	-1.829016000	-1.601345000	2.353050000
H	-3.531302000	-1.258517000	2.715151000
C	3.461886000	-2.659183000	-2.439582000
H	3.819928000	-1.619295000	-2.491845000
H	4.045832000	-3.266916000	-3.145292000
H	2.414915000	-2.643718000	-2.786418000
C	2.805689000	-2.072790000	2.548633000
H	2.733168000	-2.655766000	3.477403000
H	3.531103000	-1.258633000	2.715130000
H	1.828829000	-1.601658000	2.353163000
C	-4.231501000	2.017084000	-2.352568000
H	-4.613571000	2.594580000	-3.205885000
H	-3.325236000	1.479766000	-2.673792000
H	-4.987329000	1.256170000	-2.094253000
C	-2.070094000	2.703395000	2.171384000
H	-2.455930000	1.737656000	2.535912000
H	-1.038994000	2.513543000	1.833495000
H	-2.037137000	3.407474000	3.014513000
C	4.231727000	2.016730000	-2.352596000
H	4.613519000	2.594170000	-3.206075000
H	4.987805000	1.256075000	-2.094245000
H	3.325569000	1.479102000	-2.673598000
C	2.070082000	2.703682000	2.171167000
H	1.038857000	2.514349000	1.833361000
H	2.455519000	1.737714000	2.535502000
H	2.037524000	3.407653000	3.014402000
C	-4.942928000	0.010239000	0.513069000
C	-5.884513000	-0.814163000	-0.132989000
C	-5.380338000	0.846599000	1.558404000
C	-7.223491000	-0.803287000	0.260025000
C	-6.721337000	0.857258000	1.944049000
C	-7.647550000	0.032094000	1.298259000
H	-5.571187000	-1.458815000	-0.952507000
H	-4.667919000	1.485967000	2.077357000
H	-7.939945000	-1.448483000	-0.253865000
H	-7.041896000	1.511754000	2.758050000
H	-8.696971000	0.040606000	1.602762000
C	4.942883000	0.010186000	0.513199000
C	5.380209000	0.846575000	1.558547000
C	5.884512000	-0.814250000	-0.132748000
C	6.721174000	0.857230000	1.944310000
C	7.223456000	-0.803378000	0.260384000
C	7.647433000	0.032035000	1.298626000
H	4.667747000	1.485964000	2.077417000
H	5.571250000	-1.458930000	-0.952267000
H	7.041671000	1.511748000	2.758318000
H	7.939947000	-1.448600000	-0.253420000
H	8.696828000	0.040545000	1.603220000
H	0.000011000	-0.578660000	-2.472685000
H	0.000013000	0.490299000	-2.491361000

TS-Sn$_{H2}$

***E* =** -2579.716046
***H* =** -2579.661480
***G* =** -2579.807910
N_{imag} = 1, -732.2 cm^{-1}
***SP* =** -2576.621148
G_{SP} = -2575.859857

Sn	0.000001000	-2.124926000	-1.227453000
N	-2.880970000	-1.082990000	-0.047764000
N	-2.851112000	1.081181000	-0.026160000
C	-3.633581000	0.006754000	0.227519000
C	-1.593882000	-0.710429000	-0.473901000
C	-1.579949000	0.683244000	-0.477936000
C	-3.303017000	-2.439000000	0.176427000
C	-3.730181000	-3.211766000	-0.918004000
C	-4.127062000	-4.532110000	-0.662509000
H	-4.467025000	-5.155636000	-1.493393000
C	-4.089474000	-5.054057000	0.631539000
H	-4.400395000	-6.086475000	0.810317000
C	-3.652234000	-4.265535000	1.697952000
H	-3.616880000	-4.681697000	2.708038000
C	-3.248337000	-2.940533000	1.489358000
C	-3.302302000	2.444729000	0.036619000
C	-4.095085000	2.934757000	-1.016057000
C	-4.522358000	4.267926000	-0.941634000
H	-5.136793000	4.676227000	-1.748092000
C	-4.164329000	5.073799000	0.140346000
H	-4.503296000	6.112036000	0.180734000
C	-3.369447000	4.562016000	1.168631000
H	-3.088003000	5.199055000	2.011037000
C	-2.920769000	3.235471000	1.135655000
Sn	-0.000005000	2.047800000	-1.366437000
N	2.851088000	1.081188000	-0.026129000
N	2.880974000	-1.082984000	-0.047776000
C	3.633573000	0.006764000	0.227523000
C	1.579930000	0.683243000	-0.477914000
C	1.593877000	-0.710430000	-0.473894000
C	3.302262000	2.444739000	0.036691000
C	2.920722000	3.235431000	1.135761000
C	3.369405000	4.561971000	1.168805000
H	3.087953000	5.198973000	2.011236000
C	4.164295000	5.073799000	0.140547000
H	4.503265000	6.112033000	0.180986000
C	4.522324000	4.267979000	-0.941471000
H	5.136762000	4.676317000	-1.747908000
C	4.095046000	2.934813000	-1.015960000
C	3.303032000	-2.438995000	0.176389000
C	3.248410000	-2.940542000	1.489318000
C	3.652299000	-4.265551000	1.697878000
H	3.616987000	-4.681723000	2.707962000
C	4.089483000	-5.054067000	0.631436000
H	4.400399000	-6.086491000	0.810190000
C	4.127029000	-4.532102000	-0.662605000
H	4.466957000	-5.155619000	-1.493509000
C	3.730150000	-3.211751000	-0.918067000
C	-3.745040000	-2.649940000	-2.317203000
H	-2.730468000	-2.653461000	-2.749735000
H	-4.393596000	-3.245379000	-2.975438000
H	-4.085708000	-1.603549000	-2.341212000
C	-2.764355000	-2.069933000	2.620301000
H	-2.620656000	-2.656188000	3.538672000
H	-1.809778000	-1.584647000	2.360378000

H	-3.487292000	-1.266283000	2.840408000
C	3.744974000	-2.649913000	-2.317262000
H	4.085461000	-1.603464000	-2.341251000
H	4.393661000	-3.245239000	-2.975471000
H	2.730422000	-2.653611000	-2.749841000
C	2.764514000	-2.069945000	2.620300000
H	2.620869000	-2.656206000	3.538675000
H	3.487483000	-1.266312000	2.840365000
H	1.809928000	-1.584639000	2.360447000
C	-4.468302000	2.048113000	-2.176778000
H	-4.890645000	2.635861000	-3.003716000
H	-3.593043000	1.495544000	-2.553264000
H	-5.220200000	1.298831000	-1.876216000
C	-2.049232000	2.669774000	2.225381000
H	-2.438259000	1.713303000	2.610453000
H	-1.042844000	2.453997000	1.833611000
H	-1.952990000	3.371524000	3.065583000
C	4.468269000	2.048221000	-2.176720000
H	4.890593000	2.636009000	-3.003639000
H	5.220183000	1.298941000	-1.876194000
H	3.593016000	1.495651000	-2.553219000
C	2.049211000	2.669654000	2.225467000
H	1.042992000	2.453333000	1.833568000
H	2.438584000	1.713453000	2.610871000
H	1.952517000	3.371580000	3.065470000
C	-5.020633000	0.022448000	0.715711000
C	-6.002251000	-0.793314000	0.119918000
C	-5.395119000	0.857029000	1.786744000
C	-7.317389000	-0.776262000	0.586386000
C	-6.712626000	0.873927000	2.246237000
C	-7.678383000	0.057055000	1.649808000
H	-5.739317000	-1.436235000	-0.718381000
H	-4.651743000	1.490487000	2.267962000
H	-8.065070000	-1.414991000	0.110227000
H	-6.983477000	1.527078000	3.079183000
H	-8.709247000	0.070479000	2.012047000
C	5.020639000	0.022464000	0.715674000
C	5.395157000	0.857047000	1.786694000
C	6.002242000	-0.793291000	0.119848000
C	6.712679000	0.873948000	2.246146000
C	7.317395000	-0.776236000	0.586274000
C	7.678420000	0.057080000	1.649687000
H	4.651795000	1.490502000	2.267936000
H	5.739286000	-1.436209000	-0.718447000
H	6.983554000	1.527100000	3.079084000
H	8.065062000	-1.414961000	0.110090000
H	8.709295000	0.070506000	2.011894000
H	0.000009000	-0.618830000	-2.614111000
H	0.000010000	0.449567000	-2.641412000

TS-SiGe$_{H2}$

***E* =** -4517.158770
***H* =** -4517.106044
***G* =** -4517.246267
***N*$_{imag}$ =** 1, -465.2 cm^{-1}
***SP* =** -4513.933452
***G*$_{SP}$ =** -4513.164150

Si	0.000022000	1.895043000	-1.118726000
N	2.714426000	1.155462000	-0.186534000
N	2.738720000	-1.013515000	-0.122321000

C	3.507370000	0.087173000	0.049241000
C	1.405007000	0.743616000	-0.500566000
C	1.428225000	-0.654721000	-0.476523000
C	3.124392000	2.524535000	-0.022628000
C	3.456462000	3.276932000	-1.162821000
C	3.858596000	4.605373000	-0.965802000
H	4.125436000	5.214537000	-1.833222000
C	3.916395000	5.153985000	0.316611000
H	4.230600000	6.192329000	0.449719000
C	3.568964000	4.385358000	1.429543000
H	3.606041000	4.822894000	2.430497000
C	3.162732000	3.053081000	1.279682000
C	3.228689000	-2.364777000	-0.077492000
C	3.966346000	-2.842131000	-1.175127000
C	4.439117000	-4.160503000	-1.116034000
H	5.013399000	-4.558620000	-1.956457000
C	4.174516000	-4.965492000	-0.006875000
H	4.548278000	-5.992132000	0.021554000
C	3.427142000	-4.468462000	1.063325000
H	3.216635000	-5.105764000	1.926034000
C	2.935875000	-3.156755000	1.047431000
Ge	0.000039000	-1.905020000	-1.169804000
N	-2.738665000	-1.013540000	-0.122342000
N	-2.714423000	1.155439000	-0.186611000
C	-3.507343000	0.087135000	0.049191000
C	-1.428182000	-0.654722000	-0.476569000
C	-1.404994000	0.743615000	-0.500630000
C	-3.228595000	-2.364814000	-0.077429000
C	-2.935777000	-3.156678000	1.047575000
C	-3.427044000	-4.468380000	1.063611000
H	-3.216527000	-5.105596000	1.926380000
C	-4.174425000	-4.965515000	-0.006540000
H	-4.548188000	-5.992151000	0.021995000
C	-4.439015000	-4.160644000	-1.115785000
H	-5.013292000	-4.558850000	-1.956169000
C	-3.966240000	-2.842277000	-1.175018000
C	-3.124412000	2.524499000	-0.022646000
C	-3.162956000	3.052922000	1.279707000
C	-3.569256000	4.385172000	1.429637000
H	-3.606483000	4.822614000	2.430627000
C	-3.916570000	5.153888000	0.316732000
H	-4.230830000	6.192208000	0.449893000
C	-3.858584000	4.605393000	-0.965724000
H	-4.125342000	5.214624000	-1.833122000
C	-3.456367000	3.276989000	-1.162815000
C	3.361481000	2.682761000	-2.544171000
H	2.306031000	2.620639000	-2.859739000
H	3.904330000	3.294879000	-3.278273000
H	3.757052000	1.655629000	-2.582415000
C	2.769772000	2.203628000	2.460778000
H	2.705261000	2.805130000	3.378270000
H	1.793568000	1.722243000	2.287749000
H	3.503053000	1.398033000	2.634380000
C	-3.361244000	2.682927000	-2.544202000
H	-3.757086000	1.655902000	-2.582623000
H	-3.903778000	3.295260000	-3.278357000
H	-2.305749000	2.620552000	-2.859566000
C	-2.770231000	2.203356000	2.460803000
H	-2.705284000	2.804895000	3.378241000
H	-3.503916000	1.398162000	2.634574000
H	-1.794304000	1.721450000	2.287682000

C	4.231448000	-1.959738000	-2.368378000
H	4.611313000	-2.546295000	-3.216494000
H	3.314915000	-1.439252000	-2.688095000
H	4.980669000	-1.185536000	-2.131157000
C	2.107738000	-2.611796000	2.180546000
H	2.458160000	-1.620401000	2.509773000
H	1.064574000	-2.474724000	1.853028000
H	2.115850000	-3.290870000	3.044581000
C	-4.231338000	-1.960004000	-2.368358000
H	-4.611197000	-2.546646000	-3.216417000
H	-4.980557000	-1.185776000	-2.131219000
H	-3.314802000	-1.439551000	-2.688122000
C	-2.107676000	-2.611532000	2.180627000
H	-1.064778000	-2.473302000	1.852757000
H	-2.458905000	-1.620625000	2.510491000
H	-2.114823000	-3.291032000	3.044332000
C	4.922999000	0.111945000	0.447007000
C	5.845385000	0.937749000	-0.224045000
C	5.381359000	-0.693905000	1.507121000
C	7.187158000	0.957639000	0.159040000
C	6.724982000	-0.673489000	1.883077000
C	7.632425000	0.152548000	1.212209000
H	5.515051000	1.559402000	-1.054564000
H	4.683172000	-1.333397000	2.045142000
H	7.889004000	1.603333000	-0.374000000
H	7.062367000	-1.304166000	2.708974000
H	8.683964000	0.168303000	1.509016000
C	-4.922999000	0.111893000	0.446868000
C	-5.381454000	-0.693994000	1.506913000
C	-5.845331000	0.937723000	-0.224228000
C	-6.725107000	-0.673584000	1.882761000
C	-7.187134000	0.957610000	0.158750000
C	-7.632492000	0.152484000	1.211855000
H	-4.683324000	-1.333516000	2.044969000
H	-5.514932000	1.559403000	-1.054700000
H	-7.062560000	-1.304292000	2.708606000
H	-7.888932000	1.603326000	-0.374325000
H	-8.684055000	0.168234000	1.508578000
H	0.000037000	0.584313000	-2.465377000
H	0.000043000	-0.440769000	-2.504284000

TS-SiSn$_{H2}$

E **=** -2654.744867
H **=** -2654.691754
G **=** -2654.833759
***N*$_{imag}$** **=** 1, 277.9 cm^{-1}
SP **=** -2651.993166
***G*$_{SP}$** **=** -2651.225701

Si	0.000001000	1.845397000	-1.178177000
N	2.705062000	1.209536000	-0.194268000
N	2.831593000	-0.950108000	-0.060099000
C	3.539873000	0.191218000	0.106397000
C	1.427986000	0.721523000	-0.543984000
C	1.521981000	-0.672421000	-0.479551000
C	3.047909000	2.600708000	-0.066486000
C	3.396671000	3.323668000	-1.220757000
C	3.739920000	4.673052000	-1.059261000
H	4.017286000	5.260404000	-1.938356000
C	3.724577000	5.270141000	0.202686000
H	3.993201000	6.324328000	0.308257000

C	3.361086000	4.529796000	1.329512000
H	3.339908000	5.004992000	2.313626000
C	3.013201000	3.177655000	1.214931000
C	3.379199000	-2.274390000	0.053131000
C	4.189516000	-2.752809000	-0.992178000
C	4.708911000	-4.049279000	-0.870841000
H	5.339318000	-4.447496000	-1.669986000
C	4.421490000	-4.832614000	0.248064000
H	4.832496000	-5.842355000	0.325236000
C	3.605596000	-4.334950000	1.266547000
H	3.379865000	-4.954747000	2.138106000
C	3.064822000	-3.044943000	1.187850000
Sn	0.000000000	-2.127723000	-1.268282000
N	-2.831594000	-0.950107000	-0.060101000
N	-2.705060000	1.209538000	-0.194266000
C	-3.539872000	0.191220000	0.106396000
C	-1.521982000	-0.672420000	-0.479552000
C	-1.427985000	0.721524000	-0.543984000
C	-3.379204000	-2.274388000	0.053123000
C	-3.064829000	-3.044946000	1.187839000
C	-3.605609000	-4.334952000	1.266531000
H	-3.379880000	-4.954754000	2.138087000
C	-4.421504000	-4.832608000	0.248046000
H	-4.832515000	-5.842348000	0.325213000
C	-4.708923000	-4.049267000	-0.870856000
H	-5.339330000	-4.447479000	-1.670003000
C	-4.189522000	-2.752799000	-0.992187000
C	-3.047904000	2.600710000	-0.066479000
C	-3.013195000	3.177650000	1.214941000
C	-3.361077000	4.529792000	1.329529000
H	-3.339897000	5.004983000	2.313646000
C	-3.724566000	5.270143000	0.202707000
H	-3.993188000	6.324330000	0.308283000
C	-3.739911000	4.673060000	-1.059243000
H	-4.017276000	5.260418000	-1.938335000
C	-3.396664000	3.323677000	-1.220746000
C	3.372967000	2.676409000	-2.581004000
H	2.331043000	2.548313000	-2.921282000
H	3.904459000	3.289347000	-3.322649000
H	3.821809000	1.670636000	-2.570264000
C	2.602107000	2.357542000	2.410270000
H	2.485611000	2.987796000	3.303013000
H	1.647154000	1.841504000	2.218230000
H	3.351590000	1.581068000	2.638469000
C	-3.372962000	2.676426000	-2.580996000
H	-3.821793000	1.670647000	-2.570260000
H	-3.904464000	3.289362000	-3.322635000
H	-2.331038000	2.548342000	-2.921281000
C	-2.602103000	2.357532000	2.410277000
H	-2.485603000	2.987781000	3.303022000
H	-3.351588000	1.581059000	2.638473000
H	-1.647151000	1.841490000	2.218233000
C	4.485361000	-1.893627000	-2.195301000
H	4.914269000	-2.491470000	-3.011598000
H	3.573437000	-1.400392000	-2.566888000
H	5.207008000	-1.097603000	-1.945191000
C	2.165894000	-2.503463000	2.268169000
H	2.441123000	-1.477408000	2.559590000
H	1.126062000	-2.449491000	1.906466000
H	2.189918000	-3.140824000	3.163222000
C	-4.485364000	-1.893611000	-2.195307000

H	-4.914272000	-2.491449000	-3.011607000
H	-5.207010000	-1.097587000	-1.945195000
H	-3.573438000	-1.400376000	-2.566890000
C	-2.165897000	-2.503477000	2.268160000
H	-1.126063000	-2.449528000	1.906462000
H	-2.441108000	-1.477414000	2.559574000
H	-2.189937000	-3.140832000	3.163217000
C	4.934741000	0.296666000	0.559840000
C	5.847488000	1.141747000	-0.100431000
C	5.382654000	-0.452484000	1.665170000
C	7.169417000	1.235229000	0.336939000
C	6.706684000	-0.358421000	2.095561000
C	7.604520000	0.485975000	1.434691000
H	5.525748000	1.720960000	-0.964402000
H	4.690614000	-1.105068000	2.195669000
H	7.864137000	1.894866000	-0.188344000
H	7.035991000	-0.945646000	2.956056000
H	8.640636000	0.559541000	1.773995000
C	-4.934741000	0.296670000	0.559840000
C	-5.382654000	-0.452480000	1.665169000
C	-5.847486000	1.141753000	-0.100430000
C	-6.706684000	-0.358416000	2.095561000
C	-7.169415000	1.235236000	0.336940000
C	-7.604519000	0.485982000	1.434692000
H	-4.690614000	-1.105066000	2.195668000
H	-5.525745000	1.720966000	-0.964401000
H	-7.035991000	-0.945642000	2.956055000
H	-7.864134000	1.894875000	-0.188342000
H	-8.640635000	0.559549000	1.773996000
H	0.000000000	0.556419000	-2.530906000
H	0.000000000	-0.444030000	-2.640914000

TS-GeSn$_{H2}$

***E* =** -4442.130542
***H* =** -4442.076479
***G* =** -4442.221535
N_{imag} = 1, 640.3 cm^{-1}
***SP* =** -4438.561972
G_{SP} = -4437.798610

Ge	-0.000010000	1.894018000	-1.218885000
N	2.744575000	1.141997000	-0.134665000
N	2.832691000	-1.020426000	-0.038015000
C	3.557107000	0.104283000	0.167970000
C	1.473534000	0.682561000	-0.526332000
C	1.538431000	-0.710419000	-0.484356000
C	3.098502000	2.525839000	0.031853000
C	3.475060000	3.273063000	-1.097950000
C	3.817463000	4.617885000	-0.898905000
H	4.117109000	5.223385000	-1.758159000
C	3.774138000	5.187366000	0.374954000
H	4.042247000	6.238345000	0.509675000
C	3.384127000	4.423251000	1.476824000
H	3.341891000	4.876822000	2.470418000
C	3.036064000	3.074879000	1.324698000
C	3.356116000	-2.355712000	0.059799000
C	4.177174000	-2.828404000	-0.979467000
C	4.673408000	-4.135210000	-0.872562000
H	5.311366000	-4.529419000	-1.667691000
C	4.353811000	-4.933448000	0.226920000
H	4.746883000	-5.951098000	0.292811000

C	3.528596000	-4.440474000	1.240231000
H	3.277703000	-5.071698000	2.096566000
C	3.010576000	-3.140484000	1.175218000
Sn	0.000004000	-2.130192000	-1.327595000
N	-2.832693000	-1.020442000	-0.037994000
N	-2.744597000	1.141980000	-0.134685000
C	-3.557116000	0.104265000	0.167986000
C	-1.538441000	-0.710431000	-0.484357000
C	-1.473556000	0.682549000	-0.526354000
C	-3.356100000	-2.355734000	0.059839000
C	-3.010492000	-3.140511000	1.175234000
C	-3.528495000	-4.440507000	1.240267000
H	-3.277549000	-5.071735000	2.096584000
C	-4.353760000	-4.933482000	0.226996000
H	-4.746818000	-5.951136000	0.292902000
C	-4.673424000	-4.135240000	-0.872462000
H	-5.311420000	-4.529450000	-1.667560000
C	-4.177209000	-2.828427000	-0.979386000
C	-3.098532000	2.525830000	0.031743000
C	-3.036094000	3.074961000	1.324547000
C	-3.384142000	4.423349000	1.476573000
H	-3.341903000	4.876992000	2.470134000
C	-3.774144000	5.187385000	0.374646000
H	-4.042239000	6.238378000	0.509288000
C	-3.817478000	4.617810000	-0.899171000
H	-4.117119000	5.223250000	-1.758468000
C	-3.475090000	3.272970000	-1.098117000
C	3.486381000	2.657403000	-2.473525000
H	2.457916000	2.571327000	-2.863063000
H	4.067626000	3.269674000	-3.177509000
H	3.901962000	1.637713000	-2.468776000
C	2.597390000	2.229661000	2.492609000
H	2.455346000	2.841679000	3.394281000
H	1.650248000	1.713323000	2.266243000
H	3.344310000	1.451769000	2.724513000
C	-3.486434000	2.657193000	-2.473641000
H	-3.902162000	1.637562000	-2.468823000
H	-4.067566000	3.269484000	-3.177700000
H	-2.457968000	2.570933000	-2.863132000
C	-2.597441000	2.229820000	2.492523000
H	-2.455394000	2.841903000	3.394151000
H	-3.344380000	1.451961000	2.724476000
H	-1.650307000	1.713447000	2.266206000
C	4.508565000	-1.952176000	-2.160745000
H	4.946302000	-2.540960000	-2.978931000
H	3.610885000	-1.440118000	-2.541478000
H	5.234969000	-1.170206000	-1.881638000
C	2.103916000	-2.600407000	2.249479000
H	2.409041000	-1.594954000	2.580824000
H	1.077144000	-2.495975000	1.863702000
H	2.080762000	-3.265615000	3.123994000
C	-4.508680000	-1.952196000	-2.160639000
H	-4.946401000	-2.540993000	-2.978826000
H	-5.235128000	-1.170278000	-1.881499000
H	-3.611042000	-1.440075000	-2.541386000
C	-2.103791000	-2.600420000	2.249452000
H	-1.077080000	-2.495792000	1.863568000
H	-2.409033000	-1.595048000	2.580938000
H	-2.080442000	-3.265717000	3.123894000
C	4.943820000	0.179525000	0.650260000
C	5.878877000	1.029650000	0.028314000

C	5.363279000	-0.602488000	1.744074000
C	7.193518000	1.096382000	0.491671000
C	6.680321000	-0.535478000	2.200215000
C	7.599960000	0.314482000	1.577578000
H	5.580206000	1.633622000	-0.826915000
H	4.654557000	-1.259987000	2.245468000
H	7.905308000	1.760475000	-0.004353000
H	6.986971000	-1.148466000	3.051081000
H	8.630512000	0.366881000	1.937154000
C	-4.943813000	0.179507000	0.650319000
C	-5.363254000	-0.602554000	1.744106000
C	-5.878877000	1.029686000	0.028453000
C	-6.680279000	-0.535545000	2.200293000
C	-7.193501000	1.096418000	0.491856000
C	-7.599924000	0.314465000	1.577732000
H	-4.654532000	-1.260092000	2.245446000
H	-5.580222000	1.633710000	-0.826745000
H	-6.986912000	-1.148573000	3.051137000
H	-7.905293000	1.760554000	-0.004108000
H	-8.630464000	0.366864000	1.937345000
H	0.000004000	0.538451000	-2.553370000
H	0.000003000	-0.507985000	-2.644659000

TS-Si$_{H2}$-alternative

E **=** -2729.712402
H **=** -2729.659397
G **=** -2729.800439
N**imag** **=** 1, 435.4 cm^{-1}
SP **=** -2727.294562
G**SP** **=** -2726.526801

Si	0.000009000	-1.986149000	0.288041000
N	-2.753624000	-1.099509000	0.095219000
N	-2.769416000	1.075088000	0.115945000
C	-3.556699000	-0.018387000	0.033536000
C	-1.389764000	-0.717189000	0.207286000
C	-1.423086000	0.697269000	0.197804000
C	-3.224160000	-2.459394000	0.133005000
C	-3.154071000	-3.241300000	-1.034848000
C	-3.632793000	-4.556834000	-0.958623000
H	-3.595563000	-5.187618000	-1.850424000
C	-4.151259000	-5.064631000	0.233592000
H	-4.518189000	-6.093398000	0.271795000
C	-4.201358000	-4.266667000	1.378264000
H	-4.602468000	-4.671317000	2.310922000
C	-3.735424000	-2.946026000	1.350010000
C	-3.207851000	2.441305000	-0.007349000
C	-3.381612000	2.965475000	-1.302599000
C	-3.791893000	4.305091000	-1.395385000
H	-3.937954000	4.745499000	-2.384700000
C	-3.998928000	5.075353000	-0.250805000
H	-4.317274000	6.116523000	-0.348251000
C	-3.793430000	4.530262000	1.019296000
H	-3.947035000	5.143392000	1.910915000
C	-3.386003000	3.197675000	1.166118000
Si	0.000016000	1.830464000	0.353476000
N	2.769436000	1.075090000	0.115884000
N	2.753653000	-1.099508000	0.095149000
C	3.556730000	-0.018381000	0.033475000
C	1.423110000	0.697264000	0.197756000
C	1.389791000	-0.717193000	0.207236000

C	3.207833000	2.441324000	-0.007405000
C	3.385945000	3.197702000	1.166063000
C	3.793309000	4.530309000	1.019244000
H	3.946882000	5.143444000	1.910866000
C	3.998783000	5.075413000	-0.250854000
H	4.317077000	6.116600000	-0.348297000
C	3.791787000	4.305144000	-1.395435000
H	3.937826000	4.745562000	-2.384749000
C	3.381570000	2.965508000	-1.302655000
C	3.224179000	-2.459398000	0.132943000
C	3.735405000	-2.946049000	1.349951000
C	4.201311000	-4.266704000	1.378200000
H	4.602393000	-4.671372000	2.310861000
C	4.151213000	-5.064655000	0.233521000
H	4.518113000	-6.093433000	0.271722000
C	3.632778000	-4.556835000	-0.958703000
H	3.595544000	-5.187612000	-1.850508000
C	3.154096000	-3.241291000	-1.034922000
C	-2.573918000	-2.699197000	-2.315297000
H	-1.471585000	-2.726942000	-2.274472000
H	-2.902150000	-3.291722000	-3.181060000
H	-2.844009000	-1.646152000	-2.486509000
C	-3.781580000	-2.064962000	2.572106000
H	-2.819904000	-1.550673000	2.727110000
H	-4.556334000	-1.286127000	2.470151000
H	-4.011368000	-2.650376000	3.473227000
C	2.574030000	-2.699079000	-2.315361000
H	2.845240000	-1.646397000	-2.487084000
H	2.901290000	-3.292287000	-3.181019000
H	1.471683000	-2.725557000	-2.274109000
C	3.781546000	-2.064997000	2.572055000
H	4.011368000	-2.650411000	3.473168000
H	4.556267000	-1.286129000	2.470096000
H	2.819851000	-1.550748000	2.727074000
C	-3.056080000	2.154334000	-2.522281000
H	-3.478304000	2.616015000	-3.426568000
H	-1.941196000	2.103580000	-2.644667000
H	-3.431849000	1.121812000	-2.453586000
C	-3.117692000	2.608152000	2.528890000
H	-3.544241000	1.599017000	2.640879000
H	-2.032188000	2.502999000	2.695967000
H	-3.526991000	3.246718000	3.324355000
C	3.056065000	2.154367000	-2.522342000
H	3.478265000	2.616072000	-3.426628000
H	3.431870000	1.121858000	-2.453659000
H	1.941177000	2.103577000	-2.644720000
C	3.117664000	2.608175000	2.528840000
H	3.544134000	1.599003000	2.640788000
H	3.527063000	3.246695000	3.324291000
H	2.032161000	2.503114000	2.695979000
C	-5.019946000	-0.023293000	-0.120389000
C	-5.631511000	-0.837710000	-1.092577000
C	-5.830408000	0.786816000	0.697389000
C	-7.018448000	-0.840806000	-1.239571000
C	-7.217531000	0.779461000	0.545214000
C	-7.816194000	-0.033683000	-0.421806000
H	-5.021576000	-1.457743000	-1.746886000
H	-5.379129000	1.417356000	1.460892000
H	-7.476779000	-1.473997000	-2.002653000
H	-7.832481000	1.412225000	1.189276000
H	-8.902519000	-0.037363000	-0.539518000

C	5.019986000	-0.023274000	-0.120371000
C	5.830379000	0.786951000	0.697365000
C	5.631648000	-0.837801000	-1.092408000
C	7.217512000	0.779615000	0.545286000
C	7.018596000	-0.840878000	-1.239305000
C	7.816266000	-0.033629000	-0.421592000
H	5.379041000	1.417568000	1.460767000
H	5.021790000	-1.457944000	-1.746680000
H	7.832398000	1.412475000	1.189315000
H	7.476993000	-1.474157000	-2.002274000
H	8.902599000	-0.037294000	-0.539231000
H	0.000002000	3.108198000	-0.385811000
H	0.000010000	2.252604000	-2.632340000

TS-Ge$_{H2}$-alternative

E **=** -6304.461496
H **=** -6304.408028
G **=** -6304.549717
***N*$_{imag}$** **=** 1, 398.3 cm^{-1}
SP **=** -6300.416390
***G*$_{SP}$** **=** -6299.650023

Ge	0.000042000	-2.070446000	-0.199632000
N	2.817353000	-1.092494000	-0.056799000
N	2.822407000	1.079900000	-0.090201000
C	3.622132000	-0.007007000	-0.017839000
C	1.453264000	-0.716943000	-0.144482000
C	1.481927000	0.688671000	-0.138110000
C	3.286220000	-2.451908000	-0.100104000
C	3.240770000	-3.227906000	1.073141000
C	3.706085000	-4.547843000	0.990580000
H	3.687185000	-5.174351000	1.885963000
C	4.188521000	-5.065381000	-0.212783000
H	4.545164000	-6.097594000	-0.255907000
C	4.215598000	-4.272890000	-1.361966000
H	4.588713000	-4.685013000	-2.302961000
C	3.761135000	-2.948159000	-1.327482000
C	3.243078000	2.450859000	0.034642000
C	3.405467000	2.970312000	1.333058000
C	3.792100000	4.315061000	1.438115000
H	3.930785000	4.750545000	2.430735000
C	3.986652000	5.097075000	0.298382000
H	4.287284000	6.142774000	0.403064000
C	3.790269000	4.557368000	-0.975075000
H	3.932000000	5.179998000	-1.862090000
C	3.405424000	3.218700000	-1.132721000
Ge	0.000016000	1.876019000	-0.260387000
N	-2.822377000	1.079865000	-0.090182000
N	-2.817285000	-1.092533000	-0.056788000
C	-3.622078000	-0.007060000	-0.017806000
C	-1.481890000	0.688659000	-0.138123000
C	-1.453204000	-0.716955000	-0.144488000
C	-3.243116000	2.450811000	0.034590000
C	-3.405428000	3.218595000	-1.132817000
C	-3.790419000	4.557230000	-0.975254000
H	-3.932118000	5.179817000	-1.862304000
C	-3.987005000	5.096957000	0.298163000
H	-4.287760000	6.142627000	0.402778000
C	-3.792506000	4.314995000	1.437941000
H	-3.931355000	4.750492000	2.430532000
C	-3.405710000	2.970288000	1.332967000

C	-3.286145000	-2.451952000	-0.100154000
C	-3.761083000	-2.948130000	-1.327555000
C	-4.215530000	-4.272863000	-1.362123000
H	-4.588659000	-4.684926000	-2.303139000
C	-4.188425000	-5.065433000	-0.212995000
H	-4.545057000	-6.097647000	-0.256182000
C	-3.705973000	-4.547969000	0.990392000
H	-3.687050000	-5.174536000	1.885733000
C	-3.240665000	-3.228033000	1.073035000
C	2.705063000	-2.669605000	2.366324000
H	1.602805000	-2.630797000	2.340411000
H	3.009887000	-3.289311000	3.221462000
H	3.037953000	-1.635615000	2.544935000
C	3.780437000	-2.071702000	-2.553555000
H	2.812484000	-1.564483000	-2.692383000
H	4.551002000	-1.286783000	-2.467490000
H	3.997991000	-2.658819000	-3.456619000
C	-2.704924000	-2.669844000	2.366250000
H	-3.037688000	-1.635820000	2.544895000
H	-3.009839000	-3.289550000	3.221356000
H	-1.602661000	-2.631183000	2.340356000
C	-3.780424000	-2.071597000	-2.553573000
H	-3.998060000	-2.658649000	-3.456660000
H	-4.550955000	-1.286656000	-2.467408000
H	-2.812459000	-1.564407000	-2.692430000
C	3.107221000	2.128585000	2.541551000
H	3.413943000	2.641536000	3.464269000
H	2.010083000	1.924581000	2.600130000
H	3.621088000	1.154786000	2.502076000
C	3.140176000	2.637055000	-2.499255000
H	3.562806000	1.626854000	-2.614189000
H	2.054492000	2.536960000	-2.668118000
H	3.553768000	3.278274000	-3.290417000
C	-3.107587000	2.128596000	2.541523000
H	-3.414043000	2.641762000	3.464209000
H	-3.621812000	1.154975000	2.502207000
H	-2.010525000	1.924259000	2.600030000
C	-3.140064000	2.636887000	-2.499300000
H	-3.563051000	1.626844000	-2.614348000
H	-3.553243000	3.278268000	-3.290544000
H	-2.054380000	2.536386000	-2.667916000
C	5.087295000	-0.003117000	0.104940000
C	5.729267000	-0.835256000	1.043169000
C	5.875514000	0.832148000	-0.710622000
C	7.119127000	-0.830789000	1.158810000
C	7.265540000	0.834025000	-0.588319000
C	7.892880000	0.002958000	0.344672000
H	5.140568000	-1.476415000	1.696092000
H	5.404266000	1.475075000	-1.451036000
H	7.598826000	-1.479147000	1.895673000
H	7.860527000	1.487306000	-1.230766000
H	8.981553000	0.005585000	0.438263000
C	-5.087237000	-0.003198000	0.105008000
C	-5.875498000	0.832041000	-0.710538000
C	-5.729159000	-0.835343000	1.043267000
C	-7.265521000	0.833881000	-0.588196000
C	-7.119016000	-0.830912000	1.158946000
C	-7.892812000	0.002805000	0.344819000
H	-5.404287000	1.474984000	-1.450964000
H	-5.140419000	-1.476476000	1.696181000
H	-7.860544000	1.487141000	-1.230632000

H	-7.598678000	-1.479275000	1.895829000
H	-8.981483000	0.005403000	0.438439000
H	0.000004000	3.195382000	0.497955000
H	-0.000044000	1.752719000	2.445004000

2-Si$_{H2}$

***E* =** -2729.821932
***H* =** -2729.768984
***G* =** -2729.909069
***N*$_{imag}$ =** 0
***SP* =** -2727.413111
***G*$_{SP}$ =** -2726.639147

Si	0.000000000	1.857481000	1.111476000
N	2.715604000	1.075041000	0.204936000
N	2.742203000	-1.090491000	0.216871000
C	3.529682000	0.001068000	0.041276000
C	1.406434000	0.675707000	0.492089000
C	1.418188000	-0.718943000	0.471742000
C	3.136876000	2.448938000	0.159183000
C	2.889114000	3.190732000	-1.010049000
C	3.297602000	4.530849000	-1.024216000
H	3.121506000	5.132260000	-1.919696000
C	3.922187000	5.099698000	0.088194000
H	4.232779000	6.147203000	0.059842000
C	4.147267000	4.340227000	1.237588000
H	4.629168000	4.793272000	2.107581000
C	3.754101000	2.995880000	1.296936000
C	3.163736000	-2.451826000	0.019629000
C	3.252926000	-2.931630000	-1.299022000
C	3.654510000	-4.261438000	-1.480899000
H	3.731673000	-4.661827000	-2.494955000
C	3.945956000	-5.074322000	-0.383504000
H	4.256281000	-6.110279000	-0.541898000
C	3.838044000	-4.573432000	0.915108000
H	4.063395000	-5.216608000	1.769559000
C	3.440086000	-3.248844000	1.145165000
Si	0.000000000	-1.982413000	0.853822000
N	-2.742204000	-1.090490000	0.216871000
N	-2.715604000	1.075042000	0.204936000
C	-3.529682000	0.001069000	0.041276000
C	-1.418188000	-0.718942000	0.471742000
C	-1.406434000	0.675707000	0.492089000
C	-3.163738000	-2.451825000	0.019632000
C	-3.440087000	-3.248841000	1.145170000
C	-3.838047000	-4.573428000	0.915115000
H	-4.063397000	-5.216603000	1.769568000
C	-3.945960000	-5.074321000	-0.383496000
H	-4.256287000	-6.110278000	-0.541888000
C	-3.654516000	-4.261439000	-1.480892000
H	-3.731680000	-4.661830000	-2.494947000
C	-3.252930000	-2.931632000	-1.299018000
C	-3.136874000	2.448939000	0.159181000
C	-3.754098000	2.995883000	1.296933000
C	-4.147263000	4.340231000	1.237584000
H	-4.629163000	4.793278000	2.107575000
C	-3.922183000	5.099700000	0.088187000
H	-4.232774000	6.147205000	0.059834000
C	-3.297599000	4.530849000	-1.024222000
H	-3.121503000	5.132258000	-1.919703000
C	-2.889112000	3.190732000	-1.010052000

C	2.208500000	2.558879000	-2.196422000
H	1.161175000	2.323564000	-1.950514000
H	2.221392000	3.230180000	-3.066477000
H	2.683766000	1.607177000	-2.484135000
C	3.974395000	2.154564000	2.527315000
H	4.364259000	2.760425000	3.356987000
H	3.031896000	1.685088000	2.852004000
H	4.694087000	1.342439000	2.329878000
C	-2.208498000	2.558876000	-2.196424000
H	-2.683761000	1.607172000	-2.484134000
H	-2.221393000	3.230174000	-3.066481000
H	-1.161172000	2.323564000	-1.950516000
C	-3.974392000	2.154570000	2.527313000
H	-4.364256000	2.760433000	3.356985000
H	-4.694086000	1.342445000	2.329879000
H	-3.031894000	1.685094000	2.852003000
C	2.921112000	-2.034428000	-2.463283000
H	2.893929000	-2.600495000	-3.404801000
H	1.941997000	-1.551136000	-2.316970000
H	3.669194000	-1.230948000	-2.571188000
C	3.298000000	-2.702862000	2.542247000
H	3.729940000	-1.694091000	2.638413000
H	2.228936000	-2.612347000	2.801935000
H	3.781292000	-3.362087000	3.277116000
C	-2.921116000	-2.034432000	-2.463282000
H	-2.893937000	-2.600501000	-3.404798000
H	-3.669198000	-1.230951000	-2.571187000
H	-1.942001000	-1.551141000	-2.316971000
C	-3.297999000	-2.702856000	2.542251000
H	-2.228935000	-2.612343000	2.801937000
H	-3.729937000	-1.694084000	2.638415000
H	-3.781291000	-3.362079000	3.277121000
C	4.964390000	0.014374000	-0.263587000
C	5.495058000	0.902054000	-1.222975000
C	5.848395000	-0.864384000	0.397144000
C	6.860849000	0.909999000	-1.506591000
C	7.211825000	-0.857282000	0.102520000
C	7.726081000	0.030022000	-0.848430000
H	4.837031000	1.583261000	-1.758615000
H	5.470853000	-1.548181000	1.154765000
H	7.249049000	1.605660000	-2.254459000
H	7.877541000	-1.545955000	0.628305000
H	8.795049000	0.036273000	-1.074734000
C	-4.964390000	0.014376000	-0.263587000
C	-5.848395000	-0.864381000	0.397145000
C	-5.495057000	0.902055000	-1.222976000
C	-7.211825000	-0.857279000	0.102520000
C	-6.860848000	0.910000000	-1.506593000
C	-7.726081000	0.030024000	-0.848431000
H	-5.470854000	-1.548178000	1.154767000
H	-4.837030000	1.583260000	-1.758617000
H	-7.877541000	-1.545951000	0.628307000
H	-7.249047000	1.605660000	-2.254461000
H	-8.795049000	0.036275000	-1.074735000
H	0.000000000	2.849533000	-0.048494000
H	-0.000001000	-2.713352000	-0.492801000

2-Ge$_{H2}$

E **=** -6304.578921
H **=** -6304.524896

***G* =** -6304.668642
***N*$_{imag}$ =** 0
***SP* =** -6300.541906
***G*$_{SP}$ =** -6299.772643

Ge	0.000003000	1.935291000	-1.118156000
N	-2.761866000	1.080552000	-0.120741000
N	-2.790316000	-1.082838000	-0.132086000
C	-3.572907000	0.008219000	0.055272000
C	-1.456623000	0.678231000	-0.427903000
C	-1.470162000	-0.710737000	-0.409767000
C	-3.188217000	2.452821000	-0.070493000
C	-2.925550000	3.198390000	1.092975000
C	-3.340424000	4.536580000	1.110562000
H	-3.152422000	5.140871000	2.001673000
C	-3.985409000	5.100249000	0.007295000
H	-4.299987000	6.146492000	0.038260000
C	-4.226229000	4.336866000	-1.136330000
H	-4.724874000	4.785418000	-1.999212000
C	-3.828195000	2.994107000	-1.198490000
C	-3.218831000	-2.443914000	0.051025000
C	-3.292061000	-2.944372000	1.362895000
C	-3.701430000	-4.273812000	1.529228000
H	-3.766360000	-4.689916000	2.537777000
C	-4.015219000	-5.066907000	0.423539000
H	-4.331315000	-6.102864000	0.570007000
C	-3.921811000	-4.546285000	-0.868387000
H	-4.163961000	-5.174177000	-1.729604000
C	-3.516752000	-3.221194000	-1.082697000
Ge	-0.000002000	-2.075722000	-0.799787000
N	2.790316000	-1.082846000	-0.132097000
N	2.761872000	1.080544000	-0.120750000
C	3.572911000	0.008208000	0.055257000
C	1.470160000	-0.710741000	-0.409768000
C	1.456627000	0.678226000	-0.427908000
C	3.218824000	-2.443925000	0.051010000
C	3.516673000	-3.221224000	-1.082716000
C	3.921727000	-4.546317000	-0.868410000
H	4.163821000	-5.174225000	-1.729631000
C	4.015195000	-5.066923000	0.423518000
H	4.331285000	-6.102883000	0.569984000
C	3.701472000	-4.273810000	1.529213000
H	3.766447000	-4.689902000	2.537765000
C	3.292111000	-2.944368000	1.362884000
C	3.188227000	2.452812000	-0.070502000
C	3.828209000	2.994096000	-1.198498000
C	4.226246000	4.336853000	-1.136337000
H	4.724894000	4.785404000	-1.999218000
C	3.985427000	5.100237000	0.007287000
H	4.300008000	6.146479000	0.038253000
C	3.340439000	4.536570000	1.110554000
H	3.152437000	5.140862000	2.001665000
C	2.925561000	3.198381000	1.092967000
C	-2.219941000	2.574293000	2.268504000
H	-1.171806000	2.360199000	2.006496000
H	-2.233910000	3.242212000	3.141195000
H	-2.673248000	1.612629000	2.558177000
C	-4.071150000	2.147939000	-2.421529000
H	-4.453636000	2.754990000	-3.253767000
H	-3.141871000	1.654926000	-2.748798000
H	-4.806874000	1.352813000	-2.213582000
C	2.219947000	2.574287000	2.268494000

H	2.673251000	1.612622000	2.558169000
H	2.233915000	3.242206000	3.141185000
H	1.171813000	2.360195000	2.006483000
C	4.071163000	2.147926000	-2.421536000
H	4.453653000	2.754976000	-3.253773000
H	4.806884000	1.352797000	-2.213586000
H	3.141883000	1.654916000	-2.748807000
C	-2.930412000	-2.069814000	2.535205000
H	-2.916069000	-2.646580000	3.470479000
H	-1.936991000	-1.616730000	2.387891000
H	-3.652911000	-1.245029000	2.654957000
C	-3.388746000	-2.655501000	-2.473788000
H	-3.798197000	-1.635604000	-2.545962000
H	-2.324862000	-2.586145000	-2.757339000
H	-3.901998000	-3.291217000	-3.209067000
C	2.930534000	-2.069787000	2.535199000
H	2.916233000	-2.646539000	3.470483000
H	3.653050000	-1.245010000	2.654901000
H	1.937112000	-1.616693000	2.387931000
C	3.388588000	-2.655552000	-2.473808000
H	2.324688000	-2.586192000	-2.757297000
H	3.798043000	-1.635659000	-2.546024000
H	3.901790000	-3.291284000	-3.209109000
C	-5.004640000	0.023790000	0.381626000
C	-5.511376000	0.889473000	1.372115000
C	-5.904472000	-0.828573000	-0.290196000
C	-6.872973000	0.902155000	1.676171000
C	-7.263868000	-0.816720000	0.023580000
C	-7.755259000	0.048803000	1.005992000
H	-4.837667000	1.549519000	1.915133000
H	-5.542591000	-1.496129000	-1.069865000
H	-7.244205000	1.580320000	2.448268000
H	-7.943811000	-1.484692000	-0.510569000
H	-8.820813000	0.058575000	1.247855000
C	5.004645000	0.023779000	0.381613000
C	5.904481000	-0.828579000	-0.290212000
C	5.511378000	0.889460000	1.372104000
C	7.263876000	-0.816724000	0.023566000
C	6.872975000	0.902144000	1.676162000
C	7.755264000	0.048797000	1.005982000
H	5.542603000	-1.496133000	-1.069884000
H	4.837667000	1.549503000	1.915123000
H	7.943821000	-1.484692000	-0.510585000
H	7.244203000	1.580308000	2.448262000
H	8.820817000	0.058572000	1.247846000
H	0.000007000	2.903023000	0.152828000
H	-0.000003000	-2.684093000	0.685402000

2-Sn$_{H2}$

***E* =** -2579.743115
***H* =** -2579.688230
***G* =** -2579.834781
***N*$_{imag}$ =** 0
***SP* =** -2576.655873
***G*$_{SP}$ =** -2575.891388

Sn	-0.000006000	2.078957000	-1.269862000
N	-2.880650000	1.086766000	-0.043286000
N	-2.922602000	-1.073627000	-0.063761000
C	-3.687195000	0.019600000	0.168158000
C	-1.591704000	0.679250000	-0.419899000

C	-1.613794000	-0.708542000	-0.411316000
C	-3.303516000	2.458838000	0.030353000
C	-2.980298000	3.204038000	1.178894000
C	-3.389740000	4.543531000	1.216732000
H	-3.153828000	5.147589000	2.096505000
C	-4.088141000	5.109346000	0.147752000
H	-4.396332000	6.156928000	0.193945000
C	-4.391665000	4.345890000	-0.980880000
H	-4.934159000	4.795324000	-1.816461000
C	-4.002605000	3.001493000	-1.062043000
C	-3.359376000	-2.432528000	0.113046000
C	-3.377922000	-2.960736000	1.416372000
C	-3.791905000	-4.289911000	1.572717000
H	-3.813619000	-4.727242000	2.574075000
C	-4.164694000	-5.056387000	0.466529000
H	-4.483402000	-6.092519000	0.605789000
C	-4.127532000	-4.508612000	-0.816867000
H	-4.417249000	-5.115142000	-1.678730000
C	-3.720226000	-3.182520000	-1.021311000
Sn	0.000001000	-2.269833000	-0.833489000
N	2.922606000	-1.073617000	-0.063781000
N	2.880642000	1.086775000	-0.043289000
C	3.687195000	0.019613000	0.168137000
C	1.613792000	-0.708538000	-0.411321000
C	1.591699000	0.679255000	-0.419908000
C	3.359390000	-2.432517000	0.113010000
C	3.720232000	-3.182498000	-1.021358000
C	4.127547000	-4.508589000	-0.816929000
H	4.417258000	-5.115110000	-1.678800000
C	4.164728000	-5.056373000	0.466463000
H	4.483444000	-6.092505000	0.605711000
C	3.791948000	-4.289908000	1.572661000
H	3.813676000	-4.727247000	2.574016000
C	3.377955000	-2.960734000	1.416331000
C	3.303499000	2.458849000	0.030357000
C	4.002609000	3.001504000	-1.062024000
C	4.391662000	4.345903000	-0.980857000
H	4.934174000	4.795336000	-1.816427000
C	4.088109000	5.109362000	0.147766000
H	4.396295000	6.156945000	0.193961000
C	3.389686000	4.543547000	1.216731000
H	3.153750000	5.147608000	2.096496000
C	2.980250000	3.204052000	1.178889000
C	-2.215133000	2.580000000	2.316161000
H	-1.176272000	2.387470000	2.005350000
H	-2.200868000	3.239989000	3.194917000
H	-2.639003000	1.608065000	2.615409000
C	-4.322737000	2.154213000	-2.266881000
H	-4.716959000	2.767414000	-3.089108000
H	-3.428086000	1.622356000	-2.627613000
H	-5.078221000	1.388714000	-2.021403000
C	2.215054000	2.580019000	2.316137000
H	2.638910000	1.608081000	2.615395000
H	2.200773000	3.240008000	3.194893000
H	1.176199000	2.387495000	2.005302000
C	4.322775000	2.154220000	-2.266850000
H	4.717014000	2.767417000	-3.089070000
H	5.078255000	1.388725000	-2.021349000
H	3.428135000	1.622357000	-2.627601000
C	-2.950706000	-2.116614000	2.588554000
H	-2.925348000	-2.708267000	3.514212000

H	-1.946811000	-1.697491000	2.413990000
H	-3.640222000	-1.269980000	2.743535000
C	-3.656096000	-2.589717000	-2.406241000
H	-4.021561000	-1.551446000	-2.431065000
H	-2.613404000	-2.561063000	-2.764694000
H	-4.244656000	-3.185117000	-3.118597000
C	2.950749000	-2.116624000	2.588525000
H	2.925405000	-2.708284000	3.514178000
H	3.640263000	-1.269987000	2.743504000
H	1.946849000	-1.697504000	2.413976000
C	3.656081000	-2.589684000	-2.406281000
H	2.613384000	-2.561034000	-2.764721000
H	4.021539000	-1.551410000	-2.431102000
H	4.244636000	-3.185074000	-3.118650000
C	-5.102430000	0.043442000	0.564920000
C	-5.546182000	0.888185000	1.601663000
C	-6.045451000	-0.776935000	-0.085888000
C	-6.891241000	0.911522000	1.972206000
C	-7.388188000	-0.754121000	0.293115000
C	-7.817580000	0.090238000	1.321828000
H	-4.835761000	1.523441000	2.127555000
H	-5.730804000	-1.428298000	-0.899236000
H	-7.214596000	1.572816000	2.779628000
H	-8.103643000	-1.396978000	-0.225186000
H	-8.870003000	0.108363000	1.615285000
C	5.102436000	0.043457000	0.564882000
C	6.045453000	-0.776905000	-0.085950000
C	5.546197000	0.888186000	1.601633000
C	7.388193000	-0.754092000	0.293035000
C	6.891262000	0.911523000	1.972158000
C	7.817595000	0.090254000	1.321755000
H	5.730797000	-1.428258000	-0.899304000
H	4.835781000	1.523430000	2.127544000
H	8.103646000	-1.396937000	-0.225285000
H	7.214624000	1.572806000	2.779586000
H	8.870023000	0.108379000	1.615199000
H	-0.000003000	3.127624000	0.185746000
H	0.000003000	-2.775534000	0.897049000

2-SiGe$_{H2}$

E **=** -4517.200329
H **=** -4517.146767
G **=** -4517.289147
***N*$_{imag}$** **=** 0
SP **=** -4513.977795
***G*$_{SP}$** **=** -4513.206467

Si	0.000004000	1.873465000	-1.101293000
N	-2.722186000	1.148572000	-0.183653000
N	-2.790321000	-1.014687000	-0.158798000
C	-3.556733000	0.093833000	-0.004596000
C	-1.418350000	0.716165000	-0.461465000
C	-1.460357000	-0.674481000	-0.417239000
C	-3.118050000	2.530267000	-0.157990000
C	-2.860955000	3.282775000	1.002418000
C	-3.244564000	4.630235000	0.997637000
H	-3.060373000	5.240025000	1.885801000
C	-3.854419000	5.195996000	-0.124504000
H	-4.145475000	6.249398000	-0.111044000
C	-4.089566000	4.425788000	-1.264683000
H	-4.559963000	4.876202000	-2.142317000

C	-3.721163000	3.073750000	-1.304955000
C	-3.242894000	-2.364399000	0.050825000
C	-3.350720000	-2.828126000	1.373765000
C	-3.782461000	-4.146945000	1.566565000
H	-3.874378000	-4.534889000	2.584203000
C	-4.085059000	-4.964827000	0.475979000
H	-4.419077000	-5.991977000	0.643194000
C	-3.958012000	-4.480216000	-0.827078000
H	-4.192059000	-5.127309000	-1.676222000
C	-3.529281000	-3.167224000	-1.067964000
Ge	-0.000003000	-2.061441000	-0.761514000
N	2.790320000	-1.014698000	-0.158802000
N	2.722195000	1.148562000	-0.183672000
C	3.556738000	0.093820000	-0.004611000
C	1.460356000	-0.674487000	-0.417240000
C	1.418356000	0.716159000	-0.461476000
C	3.242881000	-2.364411000	0.050840000
C	3.529184000	-3.167282000	-1.067937000
C	3.957903000	-4.480275000	-0.827028000
H	4.191884000	-5.127405000	-1.676163000
C	4.085020000	-4.964840000	0.476039000
H	4.419026000	-5.991991000	0.643271000
C	3.782502000	-4.146913000	1.566613000
H	3.874472000	-4.534821000	2.584259000
C	3.350774000	-2.828093000	1.373790000
C	3.118065000	2.530254000	-0.158014000
C	3.721198000	3.073727000	-1.304974000
C	4.089607000	4.425763000	-1.264705000
H	4.560021000	4.876168000	-2.142335000
C	3.854447000	5.195980000	-0.124536000
H	4.145508000	6.249380000	-0.111079000
C	3.244568000	4.630231000	0.997599000
H	3.060363000	5.240029000	1.885754000
C	2.860952000	3.282773000	1.002383000
C	-2.195689000	2.653961000	2.198995000
H	-1.154137000	2.390505000	1.957407000
H	-2.193337000	3.338761000	3.058557000
H	-2.692656000	1.717951000	2.501296000
C	-3.953411000	2.220460000	-2.524882000
H	-4.329744000	2.822378000	-3.363638000
H	-3.018770000	1.729553000	-2.840522000
H	-4.688715000	1.424575000	-2.318943000
C	2.195655000	2.653971000	2.198949000
H	2.692627000	1.717977000	2.501288000
H	2.193260000	3.338789000	3.058497000
H	1.154116000	2.390492000	1.957330000
C	3.953463000	2.220427000	-2.524890000
H	4.329818000	2.822335000	-3.363642000
H	4.688755000	1.424536000	-2.318932000
H	3.018823000	1.729526000	-2.840546000
C	-3.005888000	-1.926539000	2.530741000
H	-2.999634000	-2.482925000	3.478362000
H	-2.013647000	-1.470258000	2.386216000
H	-3.734714000	-1.103827000	2.624256000
C	-3.365877000	-2.640382000	-2.470448000
H	-3.759266000	-1.617226000	-2.577369000
H	-2.295638000	-2.593317000	-2.733982000
H	-3.873495000	-3.288308000	-3.198941000
C	3.006037000	-1.926454000	2.530755000
H	2.999799000	-2.482811000	3.478392000
H	3.734909000	-1.103775000	2.624211000

H	2.013813000	-1.470127000	2.386262000
C	3.365689000	-2.640498000	-2.470432000
H	2.295434000	-2.593488000	-2.733913000
H	3.759027000	-1.617329000	-2.577410000
H	3.873297000	-3.288429000	-3.198927000
C	-4.993010000	0.140082000	0.293852000
C	-5.507751000	1.043552000	1.246469000
C	-5.892250000	-0.722098000	-0.367182000
C	-6.874330000	1.082193000	1.524357000
C	-7.256660000	-0.684089000	-0.078968000
C	-7.755297000	0.218205000	0.866054000
H	-4.837021000	1.713364000	1.780846000
H	-5.525708000	-1.417648000	-1.119622000
H	-7.250767000	1.789504000	2.267265000
H	-7.934962000	-1.360464000	-0.604594000
H	-8.824837000	0.248492000	1.087709000
C	4.993018000	0.140066000	0.293828000
C	5.892254000	-0.722112000	-0.367212000
C	5.507766000	1.043537000	1.246442000
C	7.256666000	-0.684104000	-0.079006000
C	6.874346000	1.082177000	1.524321000
C	7.755309000	0.218189000	0.866013000
H	5.525708000	-1.417661000	-1.119651000
H	4.837039000	1.713350000	1.780822000
H	7.934965000	-1.360478000	-0.604638000
H	7.250788000	1.789488000	2.267227000
H	8.824850000	0.248476000	1.087661000
H	0.000013000	2.890128000	0.036832000
H	-0.000005000	-2.624788000	0.741262000

2-SiSn$_{H2}$

***E* =** -2654.782422
***H* =** -2654.728308
***G* =** -2654.873237
N_{imag} = 0
***SP* =** -2652.034541
G_{SP} = -2651.266145

Si	0.000006000	1.807021000	-1.108770000
N	-2.732024000	1.216039000	-0.173220000
N	-2.914904000	-0.937778000	-0.109183000
C	-3.622183000	0.210654000	0.018404000
C	-1.448951000	0.706742000	-0.435883000
C	-1.566510000	-0.678364000	-0.367959000
C	-3.058963000	2.615790000	-0.169191000
C	-2.773668000	3.369742000	0.983703000
C	-3.091670000	4.733811000	0.960108000
H	-2.883746000	5.345063000	1.842029000
C	-3.665783000	5.314481000	-0.173326000
H	-3.905468000	6.380842000	-0.174825000
C	-3.930523000	4.542289000	-1.305568000
H	-4.373053000	5.003843000	-2.191890000
C	-3.627755000	3.173537000	-1.326716000
C	-3.444800000	-2.258557000	0.101969000
C	-3.599844000	-2.708694000	1.425271000
C	-4.103774000	-4.002101000	1.615996000
H	-4.232883000	-4.379195000	2.633629000
C	-4.430649000	-4.809113000	0.524350000
H	-4.820788000	-5.816389000	0.690732000
C	-4.256924000	-4.338951000	-0.778634000
H	-4.511164000	-4.977260000	-1.628612000

C	-3.756017000	-3.051454000	-1.017807000
Sn	-0.000005000	-2.322522000	-0.580898000
N	2.914899000	-0.937797000	-0.109188000
N	2.732039000	1.216021000	-0.173251000
C	3.622188000	0.210631000	0.018395000
C	1.566509000	-0.678372000	-0.367968000
C	1.448962000	0.706734000	-0.435899000
C	3.444780000	-2.258579000	0.101981000
C	3.755937000	-3.051517000	-1.017784000
C	4.256830000	-4.339015000	-0.778591000
H	4.511023000	-4.977356000	-1.628560000
C	4.430594000	-4.809142000	0.524401000
H	4.820719000	-5.816421000	0.690798000
C	4.103769000	-4.002093000	1.616035000
H	4.232905000	-4.379162000	2.633674000
C	3.599856000	-2.708683000	1.425290000
C	3.058998000	2.615768000	-0.169251000
C	3.627853000	3.173469000	-1.326768000
C	3.930643000	4.542216000	-1.305649000
H	4.373225000	5.003734000	-2.191965000
C	3.665863000	5.314448000	-0.173444000
H	3.905566000	6.380805000	-0.174965000
C	3.091685000	4.733825000	0.959980000
H	2.883726000	5.345109000	1.841871000
C	2.773657000	3.369762000	0.983603000
C	-2.146888000	2.723926000	2.191719000
H	-1.124832000	2.390513000	1.954715000
H	-2.099933000	3.423414000	3.038054000
H	-2.700303000	1.826541000	2.512720000
C	-3.895282000	2.317214000	-2.537314000
H	-4.236066000	2.926399000	-3.385993000
H	-2.984414000	1.776421000	-2.840694000
H	-4.671204000	1.562326000	-2.326032000
C	2.146769000	2.724009000	2.191597000
H	2.699987000	1.826480000	2.512528000
H	2.099986000	3.423457000	3.037975000
H	1.124632000	2.390841000	1.954592000
C	3.895433000	2.317103000	-2.537324000
H	4.236245000	2.926259000	-3.386012000
H	4.671354000	1.562230000	-2.325983000
H	2.984583000	1.776291000	-2.840720000
C	-3.224002000	-1.822068000	2.583907000
H	-3.280980000	-2.368916000	3.535314000
H	-2.197897000	-1.440047000	2.461656000
H	-3.895468000	-0.949683000	2.651521000
C	-3.544651000	-2.542196000	-2.420819000
H	-3.862293000	-1.494040000	-2.533989000
H	-2.473889000	-2.572679000	-2.683418000
H	-4.092554000	-3.154963000	-3.150573000
C	3.224068000	-1.822017000	2.583913000
H	3.281064000	-2.368839000	3.535334000
H	3.895555000	-0.949644000	2.651481000
H	2.197967000	-1.439978000	2.461683000
C	3.544512000	-2.542301000	-2.420802000
H	2.473734000	-2.572762000	-2.683343000
H	3.862177000	-1.494159000	-2.534027000
H	4.092358000	-3.155110000	-3.150565000
C	-5.057510000	0.338386000	0.301972000
C	-5.530785000	1.273139000	1.245287000
C	-5.995394000	-0.476234000	-0.364886000
C	-6.895934000	1.387436000	1.509584000

C	-7.358642000	-0.362317000	-0.090967000
C	-7.816088000	0.569904000	0.845692000
H	-4.829363000	1.908301000	1.782587000
H	-5.659559000	-1.194408000	-1.110552000
H	-7.240574000	2.117666000	2.245694000
H	-8.067849000	-1.002821000	-0.620687000
H	-8.884497000	0.659541000	1.056557000
C	5.057511000	0.338361000	0.301988000
C	5.995414000	-0.476238000	-0.364868000
C	5.530762000	1.273100000	1.245331000
C	7.358656000	-0.362317000	-0.090920000
C	6.895905000	1.387401000	1.509657000
C	7.816077000	0.569888000	0.845766000
H	5.659600000	-1.194397000	-1.110557000
H	4.829324000	1.908246000	1.782628000
H	8.067879000	-1.002804000	-0.620640000
H	7.240526000	2.117619000	2.245787000
H	8.884481000	0.659529000	1.056654000
H	0.000017000	2.876783000	-0.023023000
H	0.000000000	-2.605458000	1.203592000

2-GeSn$_{H2}$

***E* =** -4442.160958
***H* =** -4442.106406
***G* =** -4442.252332
***N*$_{imag}$ =** 0
***SP* =** -4438.599031
***G*$_{SP}$ =** -4437.832574

Ge	-0.000042000	1.861053000	-1.143979000
N	-2.772968000	1.150549000	-0.113484000
N	-2.920102000	-1.005915000	-0.078954000
C	-3.642013000	0.128529000	0.081351000
C	-1.488693000	0.667576000	-0.408632000
C	-1.580008000	-0.717566000	-0.362944000
C	-3.127307000	2.543688000	-0.086491000
C	-2.831578000	3.291343000	1.067698000
C	-3.177166000	4.648963000	1.065176000
H	-2.961267000	5.255392000	1.948490000
C	-3.788368000	5.229396000	-0.048723000
H	-4.048733000	6.290792000	-0.033807000
C	-4.064453000	4.463135000	-1.182319000
H	-4.537084000	4.924254000	-2.053206000
C	-3.735559000	3.100912000	-1.224122000
C	-3.427968000	-2.337594000	0.115335000
C	-3.552359000	-2.816228000	1.431854000
C	-4.035599000	-4.119794000	1.605786000
H	-4.140407000	-4.518917000	2.617803000
C	-4.372474000	-4.909159000	0.504282000
H	-4.745957000	-5.924816000	0.657457000
C	-4.230083000	-4.410522000	-0.791892000
H	-4.492446000	-5.034949000	-1.649695000
C	-3.750861000	-3.111770000	-1.014256000
Sn	-0.000067000	-2.340647000	-0.637938000
N	2.920013000	-1.005863000	-0.079092000
N	2.772815000	1.150613000	-0.113308000
C	3.641909000	0.128602000	0.081329000
C	1.579868000	-0.717515000	-0.362843000
C	1.488518000	0.667631000	-0.408384000
C	3.428050000	-2.337537000	0.114812000
C	3.751059000	-3.111250000	-1.015085000

C	4.230708000	-4.409904000	-0.793207000
H	4.493165000	-5.033983000	-1.651231000
C	4.373341000	-4.908932000	0.502815000
H	4.747171000	-5.924518000	0.655614000
C	4.036266000	-4.120067000	1.604591000
H	4.141219000	-4.519509000	2.616467000
C	3.552621000	-2.816562000	1.431144000
C	3.127228000	2.543738000	-0.086696000
C	3.735732000	3.100525000	-1.224422000
C	4.064746000	4.462730000	-1.183039000
H	4.537558000	4.923500000	-2.054012000
C	3.788546000	5.229406000	-0.049755000
H	4.049017000	6.290780000	-0.035150000
C	3.177124000	4.649408000	1.064247000
H	2.961184000	5.256159000	1.947332000
C	2.831411000	3.291820000	1.067196000
C	-2.164194000	2.646987000	2.254341000
H	-1.136097000	2.352238000	1.992789000
H	-2.125122000	3.334302000	3.111040000
H	-2.682469000	1.726848000	2.569372000
C	-4.021020000	2.250603000	-2.435115000
H	-4.366733000	2.865000000	-3.278019000
H	-3.119725000	1.701739000	-2.751403000
H	-4.800830000	1.501311000	-2.217695000
C	2.163720000	2.648031000	2.253970000
H	2.680417000	1.726705000	2.568067000
H	2.126545000	3.334942000	3.111082000
H	1.134867000	2.355417000	1.992946000
C	4.021503000	2.249765000	-2.435030000
H	4.366076000	2.864025000	-3.278502000
H	4.802327000	1.501545000	-2.217508000
H	3.120721000	1.699628000	-2.750525000
C	-3.166877000	-1.947369000	2.600659000
H	-3.194485000	-2.514460000	3.541489000
H	-2.150261000	-1.545369000	2.464670000
H	-3.851253000	-1.088158000	2.700430000
C	-3.574583000	-2.571394000	-2.410585000
H	-3.906417000	-1.524935000	-2.495391000
H	-2.509377000	-2.584012000	-2.696291000
H	-4.130939000	-3.175412000	-3.141276000
C	3.167011000	-1.948186000	2.600278000
H	3.194864000	-2.515608000	3.540907000
H	3.851204000	-1.088864000	2.700269000
H	2.150268000	-1.546435000	2.464550000
C	3.574609000	-2.570153000	-2.411108000
H	2.508891000	-2.578165000	-2.694995000
H	3.910813000	-1.525092000	-2.496445000
H	4.127176000	-3.176448000	-3.142769000
C	-5.073653000	0.229358000	0.396319000
C	-5.538036000	1.133204000	1.372920000
C	-6.014154000	-0.578842000	-0.273776000
C	-6.899091000	1.224856000	1.666189000
C	-7.373215000	-0.487673000	0.028596000
C	-7.822451000	0.414559000	0.997996000
H	-4.832461000	1.761524000	1.913024000
H	-5.683877000	-1.274448000	-1.043054000
H	-7.237791000	1.931322000	2.427776000
H	-8.085544000	-1.122445000	-0.503798000
H	-8.887558000	0.486309000	1.231309000
C	5.073436000	0.229489000	0.396773000
C	6.014270000	-0.578752000	-0.272803000

C	5.537385000	1.133403000	1.373528000
C	7.373195000	-0.487518000	0.030157000
C	6.898306000	1.225125000	1.667387000
C	7.821995000	0.414812000	0.999666000
H	5.684393000	-1.274488000	-1.042125000
H	4.831568000	1.761721000	1.913315000
H	8.085757000	-1.122341000	-0.501864000
H	7.236630000	1.931656000	2.429080000
H	8.886995000	0.486603000	1.233450000
H	-0.000267000	2.892206000	0.072951000
H	-0.000077000	-2.689378000	1.134193000
